# A Hexadecacationic Metal–Organic Cage for Anchoring Sulfonates: High‐Order Binding of Di‐ and Trisulfonates and High‐Performance Removal of Perfluorosulfonates from Water

**DOI:** 10.1002/advs.202515258

**Published:** 2025-10-15

**Authors:** Yawei Liu, Gen Li, Xiang Zhang, Zelin Du, Tingting Zhang, Yongya Zhang, Roy Lavendomme, En‐Qing Gao, Dawei Zhang

**Affiliations:** ^1^ State Key Laboratory of Petroleum Molecular & Process Engineering, Shanghai Key Laboratory of Green Chemistry and Chemical Processes, School of Chemistry and Molecular Engineering East China Normal University Shanghai 200062 China; ^2^ College of Chemistry Zhengzhou University Zhengzhou Henan 450001 China; ^3^ State Key Laboratory of Organometallic Chemistry, Shanghai Institute of Organic Chemistry, University of Chinese Academy of Sciences Chinese Academy of Sciences Shanghai 200032 China; ^4^ College of Chemistry and Chemical Engineering Shangqiu Normal University Shangqiu 476000 China; ^5^ Laboratoire de Chimie Organique & Laboratoire de Résonance Magnétique Nucléaire Haute Résolution Université libre de Bruxelles (ULB) Avenue F. D. Roosevelt 50, CP160/06 Brussels B‐1050 Belgium

**Keywords:** metal–organic cages, perfluoroalkyl substance removal, perfluorosulfonate adsorption, sulfonate receptors, supramolecular chemistry

## Abstract

Developing synthetic hosts capable of binding sulfonate anions or sequestering perfluorosulfonates is highly important. In this work, the self‐assembly of a tripyridinium‐tricarboxylate ligand with Cp_2_ZrCl_2_ (Cp = *η* ^5^‐C_5_H_5_) is reported, yielding a highly cationic tetrahedral cage (**1**) engineered with acidic C−H protons. The hexadecacationic cage binds a variety of sulfonate anions with unprecedented charge‐discriminative phenomena: the cage binds monosulfonates in fast exchange on the NMR timescale, while di‐ and multisulfonates exhibit slow‐exchange binding. In particular, high‐capacity complexation of di‐ and trisulfonates by **1** is achieved, with the pronounced case of **1** accommodating three dianions. Molecular modeling suggests the existence of a “Y”‐shaped binding pocket on each edge of tetrahedron **1** (six in total), with each pocket capable of anchoring a guest bearing at least two sulfonate headgroups through electrostatic and hydrogen bonding interactions. Further investigations demonstrate that **1** in solid state is an exceptional adsorbent for perfluorooctane sulfonate (PFOS), with a maximum adsorption capacity of up to 1603 mg g^−1^ positioning it among the top PFOS adsorbents. Moreover, cage **1** exhibits rapid kinetics, high selectivity, and long‐life cycles in PFOS removal, and also effectively adsorbs shorter perfluorosulfonates.

## Graphical abstract

Developing synthetic hosts that can bind sulfonate anions or sequester perfluorosulfonates is crucial in biological and environmental sciences. Here, a Zr‐based metal–organic cage (**1**) integrating electron‐deficient tripyridinium panels is designed and synthesized. The hexadecacationic (16+) cage, precisely engineered with acidic protons, demonstrates unprecedented sulfonate recognition and perfluorosulfonate remediation.

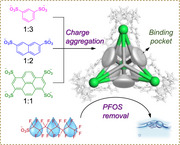

## 1 Introduction

Sulfonic acids and salts are a class of important chemical species widely found in both synthetic and natural compounds,^[^ ^1^, ^2^, ^3^, ^4^ ^]^ with applications ranging from detergents, pharmaceuticals, and biofunctional roles. The introduction of sulfonate groups can alter the solubility and reactivity of organic compounds,^[^ ^4^ ^]^ which is particularly useful in the synthesis of pharmaceuticals,^[^ ^5^ ^]^ dyes,^[^ ^6^ ^]^ and other industrial chemicals such as detergents and surfactants.^[^ ^7^ ^]^ Sulfonates are also crucial components of various biomolecules, contributing to the function and stability of proteins, enzymes, and metabolites.^[^ ^8^, ^9^ ^]^ For instance, 2‐mercaptoethanesulfonate is a crucial coenzyme in methanogenic archaea, serving as a key intermediate in the metabolic pathway of methanogenesis.^[^ ^8^ ^]^ Developing synthetic receptors or hosts capable of binding sulfonate anions is thus of significant importance, enabling to broaden the scope of sulfonate chemistry in both scientific research and practical applications.

Among various sulfonate anions, perfluorosulfonates represent a prominent subclass of per‐ and polyfluoroalkyl substances (PFAS).^[^ ^10^ ^]^ These compounds are characterized by hydrophobic C‐F chains and a hydrophilic headgroup within a single molecule and are soluble in water. PFAS, often referred to as “forever chemicals”, are notorious environmental contaminants linked to serious health issues, including liver cancer and immune system suppression.^[^ ^11^ ^]^ They are also notable for their bioaccumulation in both the environment and human tissues, as well as their potential for long‐range environmental transport.^[^ ^12^ ^]^ Unfortunately, their widespread use and persistent presence have resulted in contamination of various water sources.^[^ ^13^ ^]^ Adsorption is the most employed method for the removal of PFAS from water.^[^ ^14^, ^15^, ^16^, ^17^ ^]^ Various adsorbents have been investigated, including activated carbon,^[^ ^18^, ^19^, ^20^ ^]^ ion‐exchange resins,^[^ ^21^, ^22^, ^23^ ^]^ metal–organic frameworks (MOFs),^[^ ^24^, ^25^ ^]^ covalent organic frameworks (COFs),^[^ ^26^, ^27^, ^28^ ^]^ metal–organic cages (MOCs),^[^ ^29^, ^30^, ^31^, ^32^, ^33^, ^34^, ^35^ ^]^ and macrocycle‐based supramolecular materials.^[^ ^36^, ^37^, ^38^, ^39^, ^40^ ^]^ However, these adsorbents exhibit varying degrees of limitations, such as slow kinetics, low capacity, inefficiency for short‐chain PFAS, poor stability, or limited reusability. The development of new adsorbents that can simultaneously overcome these limitations are of urgent need.

Metal–organic cages (MOCs),^[^ ^41^, ^42^, ^43^ ^]^ a class of discrete metallosupramolecular capsules, are assembled from organic ligands with either metal ions or metal clusters. The host‐guest chemistry of their well‐defined cavities enables them to have wide applications ranging from molecular recognition,^[^ ^44^ ^]^ separation,^[^ ^45^, ^46^, ^47^, ^48^, ^49^ ^]^ stabilization of reactive species,^[^ ^50^ ^]^ and catalysis.^[^ ^51^, ^52^, ^53^, ^54^, ^55^, ^56^, ^57^, ^58^, ^59^ ^]^ MOCs have been sporadically reported as receptors for sulfonate guests,^[^ ^60^, ^61^, ^62^, ^63^, ^64^ ^]^ with the quadruply‐stranded Pd_2_L_4_ helicates being the most fruitful.^[^ ^65^, ^66^, ^67^, ^68^, ^69^, ^70^, ^71^ ^]^ Through ligand alternation, various Pd_2_L_4_ cages have been constructed and demonstrated to be capable of binding mono‐ and disulfonate anions via hydrogen bonding interactions along with hydrophobic and/or *π*–*π* interactions. Nevertheless, these cages typically exhibit simple binding modes with sulfonate guests. High‐order complexation of a wide spectrum of sulfonates, with rich variations in affinity and binding stoichiometry, particularly for di‐ and trisulfonates, has yet to be reported. Additionally, compared to MOFs and COFs, the use of solid‐state MOCs as adsorbents for sequestering PFAS from water represents a recent development, with only a limited number of studies available.^[^ ^29^, ^30^, ^31^, ^32^, ^33^, ^34^, ^35^ ^]^ A notable advantage of MOCs for this purpose is their solubility, which allows for the investigation of host‐guest interactions at the molecular level. However, the adsorption performance of MOCs for PFAS is significantly inferior to that of MOFs and COFs, particularly in adsorption capacity and recyclability.

In this work, we report a Zr‐based tetrahedral coordination cage (**1**) featuring a highly positive charge (16+) along with cavity enclosure and structural stability. The cage incorporates four tripyridinium units as faces, thus providing an inner cavity precisely engineered with acidic C−H protons for hydrogen bonding interactions. The hexadecacationic cage exhibits high‐order complexation with a variety of sulfonate anions, differing in binding affinity, stoichiometry, and guest exchange kinetics. In particular, it can aggregate two or three di‐/trisulfonates within its confined cavity. Further investigations also demonstrate that cage **1** possesses strong electrostatic and hydrogen bonding interactions with perfluoroalkyl sulfonates, with the solid form of **1** serving as a rapid, selective, and high‐capacity adsorbent for the removal of perfluorosulfonates from water.

## 2 Results and Discussion

### 2.1 Design, Synthesis, and Characterization

Owing to the high oxophilicity of Zr(IV) (Zr−O bond energy up to 766 kJ mol^−1^), the Zr‐MOCs built from Cp_3_Zr_3_O(OH)_3_ vertices (Cp = *η* ^5^‐C_5_H_5_) and multicarboxylic linkers feature high chemical stability.^[^ ^61^, ^72^, ^73^, ^74^, ^75^, ^76^, ^77^, ^78^ ^]^ Most Zr‐MOCs have poor solubility due to strong interactions between the cationic cages and counterions (generally Cl^−^), and thus they have been most often investigated for solid‐state or multiphase applications.^[^ ^73^, ^79^, ^80^, ^81^, ^82^ ^]^ Recently, we demonstrated an anion‐solubilizing strategy for self‐assembly of soluble Zr‐MOCs.^[^ ^83^ ^]^ The use of tetrakis(3,5‐bis(trifluoromethyl)phenyl)borate (BAr_F_^−^) can minimize cation‐anion interactions and thereby renders good solubility to cationic species (pyridinium‐derived carboxylic ligands, the target MOCs, or any intermediates), which is beneficial for the reversibility and the error‐correction during the self‐assembly processes.

**Figure 1:**
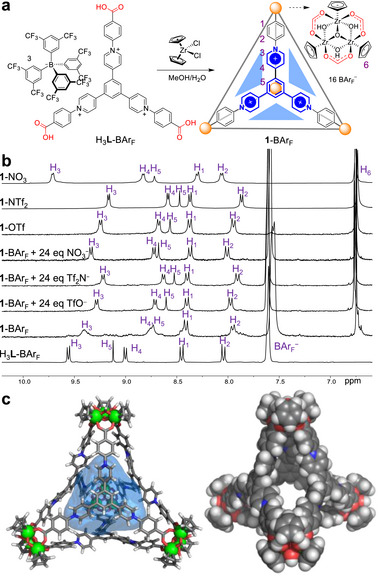
a) Self‐assembly of tetrahedron **1**. b) ^1^H NMR spectra (400 MHz, 298 K) of H_3_ **L**‐BAr_F_, **1**‐BAr_F_ in the absence or presence of TfO^−^, Tf_2_N^−^, or NO_3_^−^, **1**‐OTf, and **1**‐NTf_2_ in CD_3_OD, and **1**‐NO_3_ in DMSO‐*d*_6_. c) GFN2‐xTB‐optimized model of *T*‐symmetric **1**. The 1069 Å^3^ cavity defined by a probe of radius 2.7 Å is shown in translucent blue. External stabilizing anions are omitted for clarity (see Section S5.1, Supporting Information).

Following this method, we investigated the assembly of a new Zr‐MOC (**1**) from a tripyridinium‐tricarboxylic ligand (**Figure**  **1**a). The BAr_F_^−^ salt of the ligand (H_3_ **L**‐BAr_F_) was prepared in four steps (Figures S1–S8, Supporting Information). The stoichiometric reaction of H_3_ **L**‐BAr_F_ (1 equiv) with Cp_2_ZrCl_2_ (3 equiv) in CH_3_OH/H_2_O at 65 °C for 12 h led to a colorless solution. **1**‐BAr_F_ precipitated upon addition of water into the solution (Figures S9–S14, Supporting Information). The counteranion can be exchanged to trifluoromethanesulfonate (TfO^−^), bis(trifluoromethanesulfonyl)imide (Tf_2_N^−^), or nitrate (NO_3_^−^) by adding the tetrabutylammonium (TBA^+^) salts to **1**‐BAr_F_ in methanol. **1**‐OTf and **1**‐NTf_2_ were precipitated after addition of diethyl ether, whereas **1**‐NO_3_ precipitated directly from the methanol solution. Note that **1**‐BAr_F_, **1**‐OTf, and **1**‐NTf_2_ are soluble in various organic solvents, such as methanol, acetonitrile, acetone, and DMSO (Figures S10, S18, S23, Supporting Information). Despite the hydrophilic nature of the anion, **1**‐NO_3_ is insoluble in water and common organic solvents, except for DMSO (Figure S27, Supporting Information). We have attempted the cage assembly from H_3_ **L**‐Cl and Cp_2_ZrCl_2_ in various solvents, which led to insoluble amorphous solids that are difficult to characterize. This underscores the advantage of the anion‐solubilizing strategy.^[^ ^83^ ^]^

The ^1^H NMR spectra of **1**‐X (X = BAr_F_^−^, TfO^−^, Tf_2_N^−^, and NO_3_^−^) all exhibit a single set of five signals arising from the ligand with a *D*_3h_ axis (Figure 1b), consistent with the expected tetrahedral geometry of cage **1**. The proton signals for these compounds have been assigned by 2D NMR experiments (Figure 1b; Figures S10–S14, S18, S19, S23, S24, S27, Supporting Information). While the Cp protons (H_6_) resonate at similar fields in these compounds, the protons of the tripyridinium ligand (H_1_−H_5_) show distinct anion‐dependent chemical shifts, indicating interactions of the cage with anions through the tripyridinium moieties. The integral ratios between the signals of L and Cp (for instance, H_6_/H_5_ ≈ 5) are consistent with the C_4_L_4_ composition of the cage (C stands for the vertex cluster [Cp_3_Zr_3_O(OH)_3_]). While other compounds show sharp and well resolved resonance peaks, the peaks of **1**‐BAr_F_ are relatively broad, with some weak signals unassignable to the cage. Upon titrating TfO^−^, Tf_2_N^−^, or NO_3_^−^ to **1**‐BAr_F_, the cage signals gradually became sharp and well defined, and meanwhile the weak signals disappeared (Figure 1b; Figures S15–S17, Supporting Information). The phenomena suggest that **1**‐BAr_F_ alone suffers a certain degree of partial dissociation in solution. It can be inferred that the bulky and weakly‐interacting counteranion of BAr_F_^−^ cannot act as an anionic buffer to adequately stabilize the highly charged cationic cage, whereas the smaller anions have a stabilizing template effect. The IR spectra of the **1**‐X compounds share the common characteristic absorption bands of the carboxylate, benzene, pyridyl, and Cp groups from the cage, with the different bonds characteristic of the corresponding anions (Figure S28, Supporting Information). The low‐resolution ESI‐MS spectrum of **1**‐OTf shows a series of peaks corresponding to the cationic species [C_4_L_4_^16+^ + xTfO^−^]^(16‐x)+^ (x = 4–8) (Figure S21, Supporting Information), evidencing the successful assemly of the hexadecacationic cage. The identity of the C_4_L_4_ tetrahedral cage in **1**‐OTf and **1**‐NTf_2_ was further confirmed by high‐resolution ESI‐MS (Figures S22 and S26, Supporting Information). The homogeneity of the cage in solution was confirmed by ^1^H DOSY NMR spectra (Figures S20 and S25, Supporting Information). The cage compounds are stable in air under ambient conditions, as indicated by NMR measurements (Figures S29–S32, Supporting Information).

The structure of tetrahedron **1** was optimized by a semiempirical extended tight‐binding method^[^ ^84^ ^]^ at the GFN2‐xTB level of theory (for details, see Section S5.1, Supporting Information).^[^ ^85^ ^]^ As shown in Figure 1c and **1** has a tetrahedral framework with approximate *T*_d_‐symmetry. Four tripyridinium‐based ligands bridge four Cp_3_Zr_3_(µ_3_‐O)(µ_2_‐OH)_3_ units, with each ligand occupying a triangular face of the tetrahedron. The average distance of *µ*_3_‐O···*µ*_3_‐O is ≈23.3 Å, and the inner cavity volume is ≈1069 Å^3^ (Section S5.2, Supporting Information), calculated using the MoloVol program we developed.^[^ ^86^ ^]^ The tetrahedral cage exhibits open gaps along the edges (Figure 1c), with an average H_5_···H_5_ distance of 8.8 Å between adjacent cationic ligands around an edge. The wide gaps should allow a smooth passage of small guests without disrupting the cage's integrity. The large open cavity of **1**, surrounded by cationic charges and acidic C–H protons from pyridiniums and phenylenes, should thus provide suitable binding sites for anions.

### 2.2 High‐Order Complexation of Sulfonate Anions

**Figure 2:**
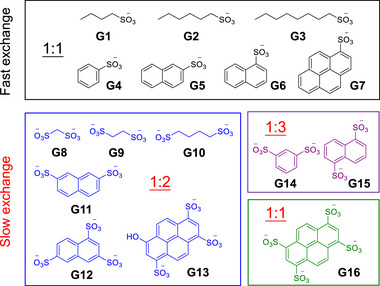
Sulfonate guests investigated in this work. The guests containing monosulfonates in the black box bind with cage **1** in fast exchange on the NMR timescale (^1^H, 400 MHz, CD_3_OD, 298 K), while the guests containing di‐, tri‐, or tetrasulfonates in the other colored boxes bind with cage **1** in slow exchange. The binding stoichiometry between the host and guest is indicated in each box.

A series of aliphatic and aromatic mono‐/multisulfonate anions (**Figure** **2**, as sodium salts) were investigated as the potential guests for **1**‐BAr_F_.

We first explored the complexation of three alkyl monosulfonates (**G1**‐**G3**). Upon titrating **G1** (butane‐1‐sulfonate) into a solution of **1**‐BAr_F_ in CD_3_OD, the ^1^H NMR spectra showed gradual shifts of the cage signals, particularly for protons H_2_–H_5_, indicating fast‐exchange binding on the NMR timescale at 400 MHz and 298 K (Figure S33, Supporting Information). The peaks of **G1** at low concentrations exhibited a significant upfield shift compared to those of free **G1**. As the concentration increased, the peaks experienced gradual downfield shifts. These observations are consistent with the shielding effect of the polyaromatic cage on the bound guest. Importantly, the initial broad peaks of **1**‐BAr_F_ became sharp and well‐defined upon titrating **G1**, demonstrating the stabilizing template role of the guest. Analysis of the titration data with BindFit ^[^ ^87^ ^]^ suggests that the binding most likely occurs in a 1:1 stoichiometry, with a binding constant of 8.7 × 10^2^ M^−1^ (Figure S33, Supporting Information; **Table** **1**). However, other binding stoichiometries due to the peripheral/surface interactions of **1** with **G1** cannot be fully ruled out. Titrations with **G2** (hexane‐1‐sulfonate) and **G3** (octane‐1‐sulfonate) into **1**‐BAr_F_ revealed similar binding phenomena (Figures S34 and S35, Supporting Information) with comparable binding constants (Table 1). The results indicate that the host‐guest complexation is almost independent of the chain size but dominated by the sulfonate headgroup, which affords electrostatic and hydrogen bonding interactions of the guests with the cage.

**Table 1:** Volumes and charges of **G1**‐**G7** and perfluoroalkyl sulfonates, and their binding behavior with cage **1**.

Guest	G1	G2	G3	G4	G5	G6	G7	PFBS	PFHxS	PFOS
**Charge**	1−	1−	1−	1−	1−	1−	1−	1−	1−	1−
**V_vdw_ (Å^3^)** ^a^	118	152	187	125	170	170	232	158	209	261
**Binding kinetics**	Fast	Fast	Fast	Fast	Fast	Fast	Fast	Fast	Fast	Fast
**Binding stoichiometry** ^c^	1:1	1:1	1:1	1:1	1:1	1:1	1:1	1:1	1:1	1:1
***K*_a_ (×10^2^ M^−1^)** ^b^	8.7	9.2	8.2	2.4	4.7	2.3	1.1	3.5	3.8	5.7

^a)^ Calculated with MoloVol based on GFN2‐xTB‐optimized structure of each guest.

^b)^ Calculated from ^1^H NMR (400 MHz, CD_3_OD, 298 K) titration data with BindFit. All errors are below 10%.

^c)^ Titration data analysis with BindFit suggests that the binding most likely occurs in a 1:1 stoichiometry, but other binding ratios due to the surface interactions with **1** cannot be ruled out.

Fast‐exchange binding phenomena with the 1:1 binding stoichiometry were also observed for **1** with a series of aryl monosulfonate guests (**G4**–**G7** in Figure 2; Figures S36–S39, Supporting Information), while the binding constants are obviously smaller than those for alkyl monosulfonates (Table 1). The weaker binding could be attributed to the delocalization of charge density from sulfonate groups to aryl skeletons and/or the steric mismatch caused by the aryl skeletons, which results in weaker electrostatic and hydrogen bonding interactions.

**Figure 3:**
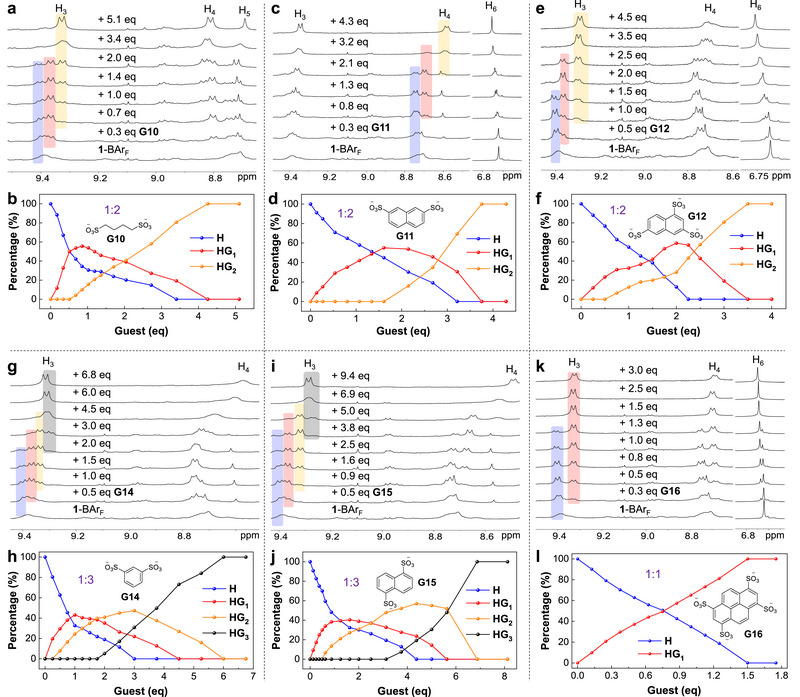
Partial ^1^H NMR (CD_3_OD, 400 MHz, 298 K) titration spectra and the corresponding species distributions of **1**‐BAr_F_ (0.25 mm) with a,b) **G10**, c,d) **G11**, e,f) **G12**, g,h) **G14**, i,j) **G15**, and k,l) **G16**. In the figures showing species distributions, **H**, **HG_1_**, **HG_2_**, and **HG_3_** represent species of free **1**, the 1:1, 1:2, and 1:3 host‐guest complexes, respectively.

Interestingly, cage **1** exhibits multiple binding events in slow exchange on the NMR timescale with guests having multisulfonate groups, in contrast to the fast‐exchange 1:1 binding with monosulfonates. As shown in **Figures** **3**a and S42 (Supporting Information), upon adding **G10** (butane‐1,4‐disulfonate) to a CD_3_OD solution of **1**‐BAr_F_, a new set of cage signals appeared, attributable to the 1:1 host‐guest complex, indicating slow exchange between bound and unbound states. The 1:1 species increased rapidly with incremental addition of **G10** and reached ≈50% of the total cage species when 0.5 equiv. **G10** was added (Figure 3b). Further addition of **G10** resulted in the appearance of another set of signals, indicating the formation of a 1:2 host‐guest complex. As the titrations progressed, the signals of the free host and the 1:1 complex gradually gave way to the signals of the 1:2 complex. Complete disappearance of the free host and the 1:1 complex occurred at 3.4 and 4.3 equiv. guest, respectively, giving rise to the sharp signals of the 1:2 species at the end. Based on the species analysis, the binding constants *K*_a1_ and *K*_a2_ were determined to be 1.4 × 10^4^ and 5.3 × 10^3^ M^−1^, indicating mild positive cooperativity for the two‐guest binding event (*K*_a1_/*K*_a2_ < 4).^[^ ^88^, ^89^ ^]^ Cage **1** also exhibits stepwise 1:1 amd 1:2 binding with smaller aliphatic disulfonate guests, **G8** (methanedisulfonate) and **G9** (ethane‐1,2‐disulfonate), showing some degree of positive cooperativity (Figures S40 and S41, Supporting Information). The binding constants correspondingly decrease with the reduction in chain length of the guests (**Table** **2**). This size effect could reflect the degree of matching between host and guest.

**Table 2:** Volumes and charges of **G8**‐**G16** and their binding behavior with cage **1**.

Guest		G8	G9	G10	G11	G12	G13	G14	G15	G16
**Charge**		2−	2−	2−	2−	3−	3−	2−	2−	4−
**V_vdw_ (Å^3^)** ^a^		100	121	156	208	245	313	162	206	343
**Binding kinetics**		Slow	Slow	Slow	Slow	Slow	Slow	Slow	Slow	Slow
**Binding stoichiometry**		1:2	1:2	1:2	1:2	1:2	1:2	1:3	1:3	1:1
**Total V_vdw_ of bound guests (Å^3^)**		200	242	312	416	490	626	486	618	343
***K*_a_ (×10^2^ M^−1^)** ^b^	*K*_a1_	4.8	9.6	140	460	62	470	180	48	250
	*K*_a2_	2.0	3.8	53	400	48	180	93	33	
	*K*_a3_							11	6.0	

^a)^ Calculated with MoloVol based on GFN2‐xTB‐optimized structure of each guest.

^b)^ Calculated from ^1^H NMR (400 MHz, CD_3_OD, 298 K) titration data with signal integrations. All errors are below 10%.

Aryl di‐/trisulfonate guests **G11** (naphthalene‐2,7‐disulfonate), **G12** (naphthalene‐1,3,6‐trisulfonate) and **G13** (8‐hydroxypyrene‐1,3,6‐trisulfonate) also form 1:1 and 1:2 host‐guest complexes with host **1** with slow‐exchange kinetics (Figure 3c,d; Figure S45, Supporting Information, for **G11**; Figure 3e,f; Figure S48, Supporting Information, for **G12**; Figure S49, Supporting Information, for **G13**). The stepwise binding constants show *K*_a1_/*K*_a2_ < 4 (Table 2), indicating positive cooperativity for the double binding events. The cooperativity is the most obvious for **G11**, which shows similar *K*_a1_ and *K*_a2_ (4.0 vs 4.6 × 10^4^ M^−1^).

Remarkably, up to three dianionic guests can be accommodated within **1**, as confirmed by the stepwise formation of 1:1, 1:2, and 1:3 host–guest complexes observed for **G14** (benzene‐1,3‐disulfonate) and **G15** (naphthalene‐1,5‐disulfonate) (Figure 3g–j; Figures S43 and S46, Supporting Information). The DOSY NMR spectra indicated that the host‐guest complexes, with one, two, or three guests bound inside the cage cavity, exhibits the same diffusion coefficient (Figures S44 and S47, Supporting Information). The 1:3 complexation corresponds to the aggregation of six negative charges in the cavity. The total volumes of three **G14** or **G15** guests were estimated to be 486 or 618 Å^3^, occupying 45% or 58%, respectively, of the cavity volume of **1**. Comparing the five naphthalene‐based guests, **G5** and **G6** (isomeric monosulfonates), **G11** and **G15** (isomeric disulfonates), and **G12** (trisulfonate), the guest binding behaviors (kinetics, affinity, and stoichiometry) of **1** are sensitive to the number of sulfonate groups and the geometry of the guests.

**Figure 4:**
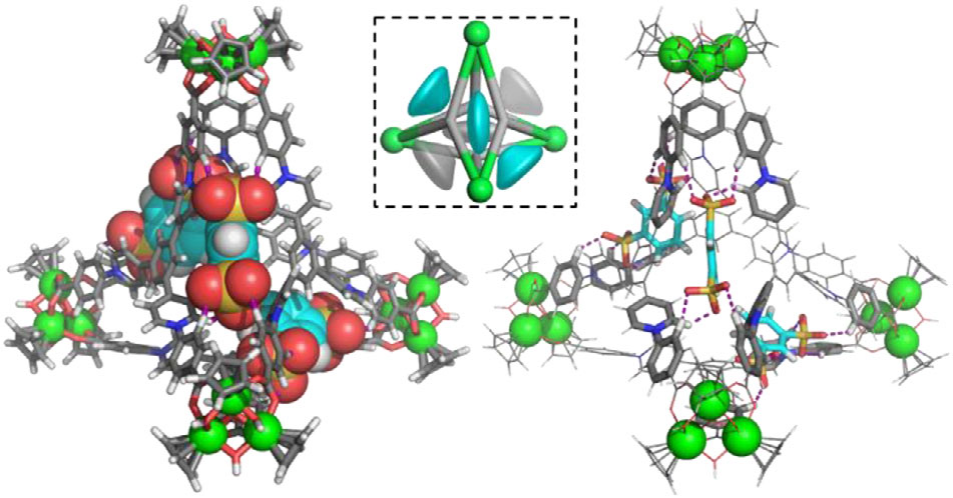
GFN2‐xTB‐optimized model of the 1:3 host–guest complex between tetrahedron **1** and **G14**, and the corresponding scheme illustrating the unoccupied (grey) and occupied (cyan) binding pockets at the edges of **1**. Hydrogen bonds are shown in purple. External stabilizing anions are omitted for clarity (see Section S5.1, Supporting Information).

To gain insight into the binding interactions, the 1:1, 1:2, and 1:3 host–guest complexes of **1** with a representative guest, **G14**, were modeled at the GFN2‐xTB level of theory. Computational results suggest that, in all of these models (**Figure** **4**; Figure S91, Supporting Information), the disulfonate guest binds within the open edges of the cage via ionic and multiple hydrogen bonding interactions. Each sulfonate headgroup forms three C─H···O hydrogen bonds (H···O distances in the range of 2.0–2.4 Å) with the acidic protons of pyridiniums or neighboring phenylenes, and the aryl core of the guest is embedded inside the cavity of **1**. The “Y”‐shaped binding pocket on each edge of the tetrahedron thus allows for versatile anchoring of a guest molecule bearing at least two sulfonate groups. The binding at the edge significantly shortens the gap between the adjacent tripyridinium ligands around the edge, with H_5_···H_5_ distances of 6.1–6.4 Å (8.8 Å for free cage). **G14** thus acts as an anionic buffer at the edge, minimizing the repulsion between cationic faces and stabilizing the highly cationic cage. The edge binding model would allow for the accommodation of up to six **G14** anions per tetrahedral cage. However, experimental results suggest the binding of up to three dianions of **G14**. We infer that the steric hindrance and electrostatic repulsion between bound anions within the cavity are unfavorable for the accommodation of more than three guests.

Finally, for the largest tetra‐anionic guest, **G16** (pyrene‐1,3,6,8‐tetrasulfonate), only slow‐exchange 1:1 binding with **1** was observed (Figure 3k,l; Figure S50, Supporting Information), in contrast with the two‐guest binding of pyrenetrisulfonate **G13**. The binding of two **G16** guests may be prevented by the stronger repulsion between highly charged guests and the larger guest volume (343 Å^3^ for **G16** vs 313 Å^3^ for **G13**).

The above results demonstrate the excellent binding properties of **1** toward sulfonate guests, with the binding kinetics, affinities, and stoichiometries being strongly dependent on the number of sulfonate groups (the charge), as well as the geometry and size of the guests. The cavity of **1** is covered by four cationic panels bearing electron‐deficient acidic protons (Figure 1a). The sulfonate headgroups behave as interacting sites. For guests possessing more than one sulfonate, abundant hydrogen bonds and strong electrostatic interactions with the cationic panels of the cage, as observed in the optimized host‐guest models (Figure 4; Figure S91, Supporting Information), result in strong binding affinities and slow guest‐exchange kinetics.

### 2.3 Complexation and Removal of Perfluorosulfonates

**Figure 5:**
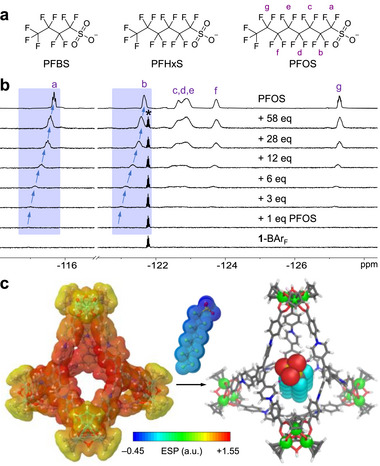
a) Perfluorosulfonate guests investigated. b) ^19^F NMR (CD_3_OD, 376 MHz, 298 K) titrations of **1**‐BAr_F_ with PFOS. The peak of internal standard 1,4‐difluorobenzene is indicated by an asterisk. c) GFN2‐xTB‐optimized model of the 1:1 host–guest complex between **1** and PFOS (right), and electrostatic potential (ESP) maps of **1** and PFOS (left).

The versatile binding of **1** with sulfonate guests inspired us to extend the studies to perfluorosulfonates in hope of developing efficient adsorbents for the persistent environmental pollutants. Three targets with different chain sizes were investigated, including perfluorooctylsulfonate (PFOS), perfluorohexylsulfonate (PFHxS), and perfluorobutylsulfonate (PFBS) (**Figure** **5**a, potassium salts).

We first explored the host–guest interactions of **1**‐BAr_F_ with these perfluorosulfonates in CD_3_OD. Titrations of **1**‐BAr_F_ with PFOS, PFHxS, and PFBS resulted in gradual upfield shifts of the ^1^H signals of **1** with concomitant peak sharpening (Figures  S51, S53, S55, Supporting Information). The phenomena are similar to those observed with non‐fluorinated monosulfonates and demonstrate the fast‐exchange binding nature and the template role of these perfluorosulfonate guests. Fast‐exchange binding was also observed by ^19^F NMR spectroscopy (Figure 5b; Figures S52, S54, S56, Supporting Information). The ^19^F signals of the fluorinated guests at low concentrations showed significant downfield shifts upon mixing with **1**. As the guest concentration increased, the signals gradually shifted upfield and finally approached those for the guests in cage‐free solutions. Analysis of the ^1^H NMR data with BindFit suggests that the binding most likely occurs in a 1:1 stoichiometry, although exterior interactions cannot be ruled out. Binding constants for the three perfluorosulfonates are similar (Table 1), but are 2–3 fold smaller than those for their non‐fluorinated analogs. This can be attributed to the electron‐withdrawing effect of the fluorine atoms, which reduces the electron density of the sulfonate groups and thus their hydrogen bonding ability.

To gain further understanding of the molecular recognition, we calculated and mapped the molecular electrostatic potential (ESP) on the van der Waals surfaces of cage **1** and PFOS (see Section S5.3, Supporting Information). As shown in Figure 5c (left), tetrahedron **1** is electron‐deficient, especially at the aromatic rings surrounding the edge pockets. PFOS, in contrast, is electron‐rich, demonstrating electrostatic complementarity with **1**. Note that the sulfonate headgroup in PFOS exhibits highly negative ESP, while its C‐F chain ends show lower negative ESP, indicating that the sulfonate group is the primary site for complexation with **1**. The GFN2‐xTB‐optimized 1:1 host–guest structure is shown in Figure 5c (right) and Figure S92 (Supporting Information) (left). PFOS binds at an open edge of the tetrahedral cage, with the linear perfluoroalkyl tail embedded into the cavity. The sulfonate head forms two C─H···O hydrogen bonds (H···O distance, 2.0 Å) with the cationic pyridinium moieties around the edge. Nevertheless, considering the significant electron‐deficiency of the cage framework (Figure 5c left), we infer that the binding of additional PFOS guests is possible through surface/peripheral interactions with **1**, as revealed by an optimized 1:2 host–guest structure (Figure S92, Supporting Information, (right)).

**Figure 6:**
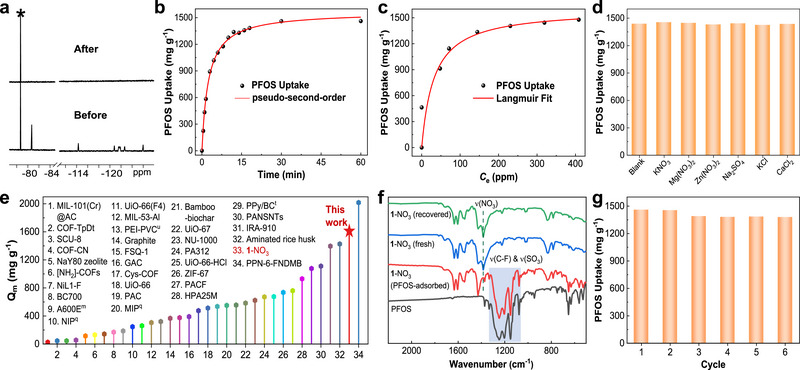
a) ^19^F NMR spectra of the PFOS solution with an initial concentration of 670 ppm (1.0 mL) before and after being treated with **1**‐NO_3_ (3.0 mg). The peak of triflate, a post‐added standard, is indicated by an asterisk. b) Sorption kinetics of **1**‐NO_3_ (3.0 mg) for PFOS with an initial concentration of 670 ppm (10 mL), fitted with a pseudo‐second‐order model. c) PFOS adsorption isotherm of **1**‐NO_3_ fitted with the Langmuir model. d) Adsorption amounts of PFOS (initial concentration 670 ppm,10 mL) by **1**‐NO_3_ (3.0 mg) in the absence or presence of various ionic species (20 equiv. relative to PFOS). e) Maximum PFOS adsorption capacity of **1**‐NO_3_ compared with those of reported adsorbents. f) IR spectra of KPFOS, **1**‐NO_3_ (fresh and recovered after six cycles), and the sample after PFOS adsorption. g) Adsorption amounts of PFOS in six consecutive cycles of adsorption with **1**‐NO_3_.

Considering the interactions of these perfluorosulfonates with **1** along with their hydrophobicity, we envisioned that a water‐insoluble solid compound of **1** might be capable of adsorbing these ionic pollutants from water. We chose water‐insoluble **1**‐NO_3_, instead of **1**‐BAr_F_, because the highly hydrophilic nitrate ion is prone to dissolution in water, which thus could provide a driving force for the adsorption of hydrophobic perfluorosulfonates through anion exchange. To evaluate the adsorption performance, a mixture of 3.0 mg of **1**‐NO_3_ and a PFOS aqueous solution (670 ppm, 1.0 mL) was stirred at room temperature. After 10 min, the aqueous solution was analyzed using ^19^F NMR, which showed the absence of fluorine signals belonging to PFOS (**Figure** **6**a; Figure S57, Supporting Information). The concentration of PFOS in the solution was then determined to be 7.1 ppm using UHPLC‐ESI‐MS (ultrahigh‐performance liquid chromatography‐electrospray ionization mass spectrometry), suggesting a removal efficiency of up to 99% (Figure S58, Supporting Information). This outstanding performance encouraged us to further investigate the adsorption kinetics of **1**‐NO_3_ in the presence of an excess of PFOS (3.0 mg **1**‐NO_3_, 670 ppm PFOS in water, 10 mL). As shown in Figure 6b and Figures S59 and S60 (Supporting Information), the adsorption proceeds rapidly, reaching an equilibrium adsorption amount of 1460 mg g^−1^ (corresponding to 17.0 PFOS per cage) within 30 min. The time to reach 80% of the equilibrium requires only 8 min. Fitting the data to a second‐order kinetics model gave a high adsorption rate constant (*k_2_*) (0.015 g mg^−1^ h^−1^) with a high correlation coefficient (*R* ^2^ = 0.99).

The equilibrium adsorption isotherm was measured at room temperature with the initial PFOS concentrations ranging from 50 ppm to saturation (670 ppm) (Figures S61 and S62, Supporting Information). Data analysis using the Langmuir model proved an excellent fit with a high correlation coefficient (0.98) (Figure 6c). The fit led to a maximum adsorption capacity of 1603 mg g^−1^ (18.7 mol mol^−1^). To our knowledge, **1**‐NO_3_ exhibits a superior adsorption capacity for PFOS compared to all previously reported metal–organic adsorbents (Figure 6e), including MOCs and MOFs.^[^ ^90^, ^91^, ^92^, ^93^, ^94^, ^95^, ^96^, ^97^, ^98^ ^]^ The capacity is also higher than those of activated carbons and purely organic materials, including porous polymers and covalent‐organic frameworks (COFs),^[^ ^18^, ^23^, ^26^, ^27^, ^28^, ^99^, ^100^, ^101^, ^102^, ^103^, ^104^, ^105^, ^106^, ^107^, ^108^ ^]^ with one exceptional case of PPN‐6‐FNDMB (reported during our manuscript preparation by the Long group (Figure 6e).^[^ ^109^ ^]^ Notably, while **1**‐NO_3_ is a cage‐based nonporous molecular material (see below), PPN‐6‐FNDMB is a highly porous polymer network functionalized with fluorinated alkylammonium groups, which may raise concerns about environmental friendliness.

The anti‐interference ability of **1**‐NO_3_ for PFOS removal was evaluated in the presence of various cationic and anionic species. The experiments were performed by stirring the mixture of **1**‐NO_3_ and an aqueous solution of PFOS in the excessive presence of a potentially interfering inorganic salt (KNO_3_, KCl, Mg(NO_3_)_2_, CaCl_2_, Na_2_SO_4_, or Zn(NO_3_)_2_, 20 equiv. relative to PFOS. For details, see Section S4.4, Supporting Information). The ^19^F NMR analysis after equilibrium adsorption demonstrates that the PFOS adsorption amounts are almost identical to those in the absence of these salts (Figure 6d; Figure S63, Supporting Information). The results indicate that common inorganic species have no significant interference with the PFOS adsorption.

The adsorption mechanism was investigated. The ^1^H and ^19^F NMR spectra after PFOS adsorption demonstrated the presence of PFOS and the structural integrity of the cage (Figures S64 and S65, Supporting Information). The IR spectrum after PFOS adsorption shows the characteristic ν(C‐F) and ν(SO_3_) adsorption of PFOS in the region of 1300−1065 cm^−1^, while the band (1384 cm^−1^) characteristic of NO_3_^−^ in **1**‐NO_3_ disappeared (Figure 6f). X‐ray photoelectronic spectroscopy (XPS) also revealed the disappearance of the N 1*s* line of NO_3_^−^ and the appearance of F 1*s* and S 2*p* signals of PFOS after adsorption (Figure S66, Supporting Information). The IR and XPS analyses suggest the occurrence of complete exchange of NO_3_^−^ by anionic PFOS. In addition, compared with the potassium salt of PFOS (KPFOS), the F 1*s* and S 2*p* signals for the PFOS‐adsorbed material shift toward lower binding energy, consistent with the changed chemical environment around PFOS.^[^ ^95^ ^]^ N_2_ adsorption measurements indicated that **1**‐NO_3_ is nonporous with a low BET surface area of 14 m^2^ g^−1^ (Figure S67, Supporting Information). Therefore, the high‐capacity uptake of PFOS cannot be attributed to adsorption into pores or onto surfaces. X‐ray diffraction (XRD) measurements revealed that both **1**‐NO_3_ and the material after PFOS adsorption are amorphous (Figure S68, Supporting Information). The two diffractograms show obviously different profiles, having broad and weak humps at different positions. This confirms that the amorphous adsorbent underwent some kind of anion‐adaptive structural transformation during adsorption. For the nonporous and non‐surficial adsorbent, the packing mode between cages should be changed to accommodate the incoming PFOS anion, which is quite different from nitrate in size, shape, and interaction properties.

We infer that the anion exchange is facilitated by the electrostatic interactions and the distinct difference between NO_3_^−^ and PFOS in hydrophilicity. The highly hydrophobic PFOS ion in water tends to enter the solid phase of **1** with hydrophobic microenvironments, while the hydrophilic NO_3_^−^ ion is prone to solvation in water. Moreover, the experimental adsorption capacity of 1460 mg g^−1^ (17.0 PFOS per cage) is larger than that expected on the basis of charge balance (16 PFOS per cage), indicating extra adsorption of PFOS as potassium salt. The analysis using ICP‐AES (inductively coupled plasma atomic emission spectrometry) confirmed the amount of K^+^ adsorbed by the adsorbent to be 1.1 K^+^ per cage, in good agreement with the extra PFOS adsorption beyond anion exchange. Zeta potential tests with the aqueous dispersions of 1‐NO_3_ and the sample after PFOS adsorption revealed that the potential changes across the zero point from +16.5 to −14.1 mV (Figure S69, Supporting Information). The positive surface charge of 1‐NO_3_ could be due to partial dissociation of the nitrate anions from the surface to water. The charge provides an electrostatic force, in collaboration with hydrophobic interactions, to drive anion exchange with PFOS. The negative surface charge after PFOS adsorption is consistent with the extra adsorption beyond anion exchange. The extra adsorption can be attributed to fluorophilicity, which refers to the specific affinity and aggregation between highly fluorinated compounds.^[^ ^28^, ^36^, ^109^ ^]^ The hexadecacationic cage adsorbent undergoes hydrophobicity‐assisted anion exchange with PFOS, and the sequestered PFOS create a fluorophilic environment to promote further adsorption, allowing the adsorption of more PFOS than dictated by the cage charge.

Adsorbent **1**‐NO_3_ can be easily recovered by stirring the PFOS‐adsorbed solid with a methanol/water (10:1, v/v) solution of KNO_3_ (see Section S4.6, Supporting Information). The adsorbent was used for six consecutive cycles of adsorption and regeneration. Notably, the adsorption capacity exhibited no significant decay (Figure 6g; Figure S70, Supporting Information). The results underscore the reusability and durability of **1**‐NO_3_ as a potential adsorbent for PFOS. The material regenerated after the repeated use was characterized to examine the structural intactness. The IR, ^1^H and ^19^F NMR spectra of the recovered sample are almost identical to those of the initial adsorbent (Figure 6f; Figures S71–S73, Supporting Information), confirming the structural integrity of the cage and the complete exchange of adsorbed PFOS by nitrate ions. The recovered sample remains amorphous. The main hump in the XRD pattern appears at the same position as observed for fresh **1**‐NO_3_, though the overall XRD profile is somewhat different (Figure S68, Supporting Information). The comparison suggests that the main feature of the amorphous solid‐state structure is regenerable, with some minor changes that do not significantly affect the adsorption performance.

The versatility of **1**‐NO_3_ as a perfluorosulfonate absorbent was demonstrated with PFBS and PFHxS. Under experimental conditions similar to those used for PFOS, the concentrations of both PFBS and PFHxS were reduced from 800 ppm to values below the detection limit of ^19^F NMR (Figures S74 and S75, Supporting Information). Further analysis using UHPLC‐ESI‐MS demonstrated residual concentrations of 7.2 and 6.0 ppm, respectively, for PFBS and PFHxS, with removal efficiencies > 99% in both cases (Figures S76 and S77, Supporting Information). The presence of the perfluoroalkyl species and the structural integrity of the cage after PFBS and PFHxS adsorption were confirmed by ^1^H and ^19^F NMR spectroscopy (Figures S78–S81, Supporting Information). Kinetics studies revealed that the material exhibits even faster adsorption. It required only 5 and 7 min to reach 80% of the equilibrium adsorption of PFBS and PFHxS, respectively (Figures S82–S85, Supporting Information). There is a trend where the shorter the perfluoroalkyl chain, the more rapidly the perfluorosulfonate is adsorbed, with rate constants (*k*_2_) of 0.068, 0.042, and 0.015 g mg^−1^ h^−1^ for PFBS, PFHxS, and PFOS, respectively. The Langmuir fit of the adsorption isotherms (Figures S86–S89, Supporting Information) suggested that the maximum adsorption capacities are 658 mg g^−1^ (12.8 mol mol^−1^) for PFBS and 1011 mg g^−1^ (14.7 mol mol^−1^) for PFHxS. Taking into account the data obtained for PFOS, both the weight and molar capacities increase with molecular weight (or chain size) of the adsorbate. This trend is consistent with the adsorption driven by hydrophobicity. Note that the saturation capacities for PFBS and PFHxS are less than expected for complete anion exchange of **1**, which also reflects the low fluorophilic and hydrophobic character of these short‐chain perfluorosulfonates.^[^ ^110^ ^]^ Nevertheless, cage **1**‐NO_3_ still shows fast kinetics and high capacities for the adsorption of PFBS and PFHxS, outperforming activated carbon^[^ ^17^, ^18^, ^20^ ^]^ and some other materials^[^ ^23^, ^25^, ^28^, ^31^, ^32^ ^]^ that are generally efficient only for long‐chain PFAS pollutants.

## 3 Conclusion

In summary, we have designed and synthesized a hexadecacationic zirconium‐based metal–organic cage (**1**). The integration of electron‐deficient tripyridinium panels and stable Cp_3_Zr_3_O(OH)_3_ vertices endowed **1** with a highly cationic, stable framework, capable of engaging sulfonate guests through synergistic electrostatic and hydrogen bonding interactions. The cage exhibited charge‐selective binding behavior: monosulfonates bind via fast‐exchange kinetics on the NMR chemical shift timescale, while di‐ and trisulfonates form high‐order complexes with slow‐exchange dynamics, achieving aggregation of up to six negative charges within the cavity. Notably, solid‐state **1**‐NO_3_ emerged as a superior adsorbent for sulfonic PFAS remediation, displaying a remarkable adsorption capacity of 1603 mg g^−1^ for PFOS. Molecular modeling revealed the importance of the “Y”‐shaped binding pockets at the edges of the tetrahedron, which can anchor sulfonate headgroups. The rapid kinetics, exceptional selectivity, high efficiency for sulfonic PFAS with both short and long chains, and reusability positioned **1** as a promising material for water treatment.

This work demonstrates how precisely engineered, highly charged MOCs can achieve unprecedented control over sulfonate recognition and environmental remediation. By enabling complexation of multiple anions in a single cavity, this study uncovers new principles for charge aggregation and cooperative binding in confined spaces. It also strengthens the capability of MOCs to tackle persistent environmental pollutants, paving the way for next‐generation materials to address pollution challenges.

## Conflict of Interest

The authors declare no conflict of interest.

## Supporting information



Supporting Information

## Data Availability

The data that support the findings of this study are available from the corresponding author upon reasonable request.
