# Supplementary material for: A Hexadecacationic Metal–Organic Cage for Anchoring Sulfonates: High‐Order Binding of Di‐ and Trisulfonates and High‐Performance Removal of Perfluorosulfonates from Water
Source: Adv Sci (Weinh). 2025 Oct 15;13(1):e15258. doi: 10.1002/advs.202515258 (PMC12767059; doi:10.1002/advs.202515258)
Supplement: Supplementary file 1 — Supporting Information [file ADVS-13-e15258-s001.pdf]

Supporting Information for

A Hexadecacationic Metal-Organic Cage for Anchoring Sulfonates: High-Order Binding of Di- and Trisulfonates and High-Performance Removal of Perfluorosulfonates from Water

Yawei Liu, Gen Li, Xiang Zhang, Zelin Du, Tingting Zhang, Yongya Zhang,* Roy Lavendomme,* En-Qing Gao,* Dawei Zhang*

Table of Contents

1. Materials and instrumentation 2
2. Synthesis and characterization 3
    2.1 Synthesis and characterization of H_3**L**-BAr_F 3
    2.2 Synthesis and characterization of **1**-BAr_F 7
    2.3 Synthesis and characterization of **1**-X (X = OTf, NTf_2, NO_3) 12
3. Guest binding studies 19
    3.1 ^{1}H NMR titrations of monosulfonates 20
    3.2 ^{1}H NMR titrations of disulfonates 27
    3.3 ^{1}H NMR titrations of trisulfonates 35
    3.4 ^{1}H NMR titrations of tetrasulfonates 37
    3.5 NMR titrations of perfluorosulfonates 38
4. Adsorption of perfluorosulfonates from water with **1**-NO_3 44
    4.1 Removal efficiency of PFOS 44
    4.2 Kinetic studies for adsorption of PFOS 45
    4.3 Isotherm adsorption experiments for PFOS 47
    4.4 Interference experiments for adsorption of PFOS 49
    4.5 Mechanism Investigation 50
    4.6 Regeneration and reuse of **1**-NO_3 53
    4.7 Removal efficiency of PFBS and PFHxS 55
    4.8 Kinetic studies for adsorption of PFBS and PFHxS 58
    4.9 Isotherm adsorption experiments for PFBS and PFHxS 60
5. Computational calculations 62
    5.1. Structure optimization 62
    5.2. Volume calculations 64
    5.3. Molecular electrostatic potential calculations 64
6. References 65

1. Materials and instrumentation

Unless otherwise specified, all reagents were purchased from commercial sources and used as received.

NMR spectra were recorded using a Bruker 400 MHz Avance III HD Smart Probe (^{1}H, ^{13}C, ^{19}F and 2D experiments). Chemical shifts for ^{1}H, ^{13}C and ^{19}F are reported in ppm on the δ scale; ^{1}H, ^{13}C and ^{19}F were referenced to the residual solvent peak. Coupling constants (J) are reported in Hz. High-resolution mass spectra were collected on an ESI-Q-TOF MS spectrometer in methanol solution. Low resolution ESI mass spectrometry was performed with a Thermo Scientific LCQ Fleet Ion Trap Mass Spectrometer.

To analyze the adsorption efficiency of the adsorbent for perfluorooctane sulfonate (PFOS), perfluorohexane sulfonate (PFHxS), and perfluorobutane sulfonate (PFBS) (potassium salts), a HSS T3 C18 column (2.1 mm x 50 mm) was coupled to a 1290 Infinity ultra-performance liquid chromatography system with a Q-Exactive quadrupole orbitrap high-resolution mass spectrometer (UHPLC-ESI-MS). The mobile phase consisted of 2 mM ammonium acetate in both water (mobile phase A) and methanol (mobile phase B) and the flow rate was 0.30 $mL{\cdot}min^{-1}$. Isocratic elution with 45% of mobile phase B was used. The column temperature was set at 50 °C and the injection volume was 15 μL.

Powder X-ray diffraction (PXRD) patterns were recorded on a Rigaku D/Max-2500 diffractometer equipped with a Cu-target tube and a graphite monochromator at 40 kV, 30 mA. Fourier transform infrared spectra (FTIR) were recorded on a Nicolet NEXUS 670 spectrophotometer using KBr pellets in the 4000-500 cm^{-1} regions. K contents were measured by inductively coupled plasma atomic emission spectroscopy (ICP-AES) using an IRIS Intrepid II XPS spectrometer.

Nitrogen adsorption-desorption isotherm measurements were done using a Micromeritics ASAP 2460 surface area analyzer. Before measurement, the samples were degassed under vacuum at 60 °C for 8 h. By using the non-local density functional theory (NLDFT) model, the pore size was derived from the sorption curve.X-ray photoelectron spectroscopy (XPS) studies were performed on AXIS SUPRA spectrometer. Zeta-potential of samples were measured in Litesizer DLS 500 (Anton Paar, Austria).

2. Synthesis and characterization

2.1 Synthesis and characterization of H_3L-BAr$_F$

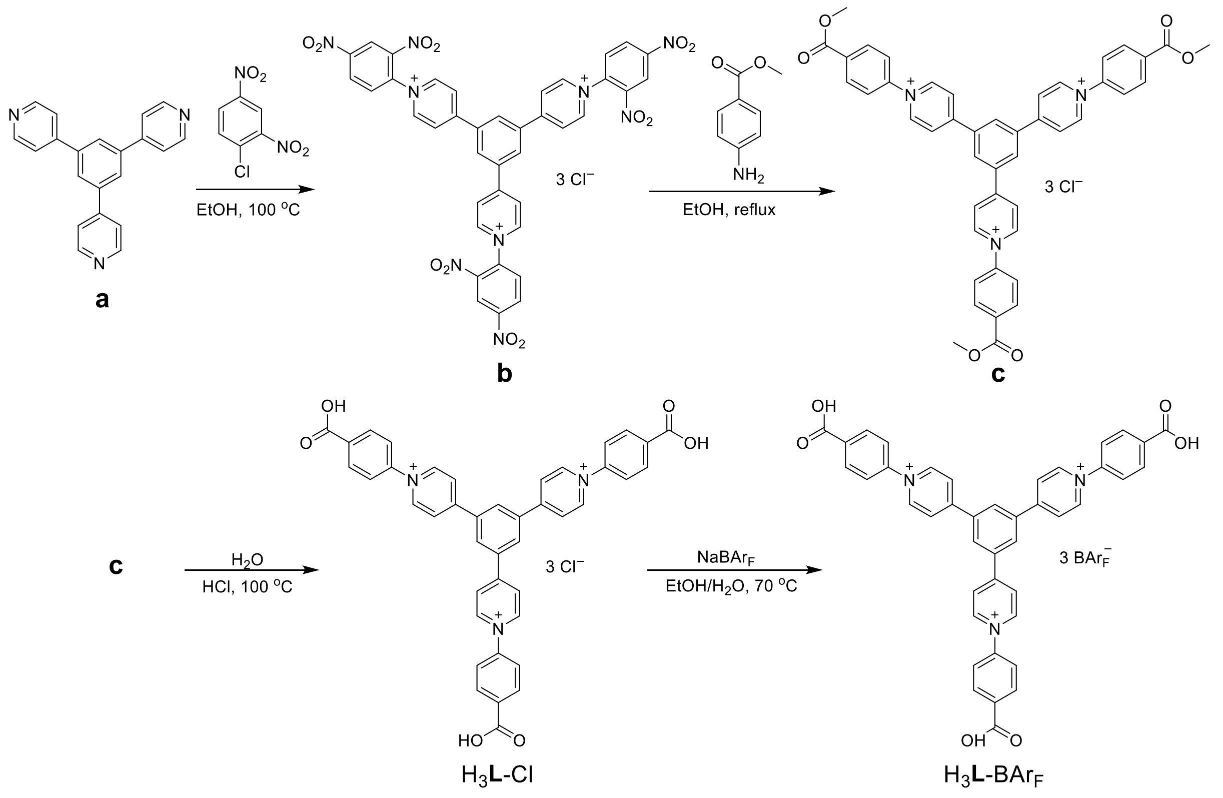

Figure S1. Synthesis of H_3**L**-BAr$_F$.

Compound b was synthesized according to the reported procedures.[1]

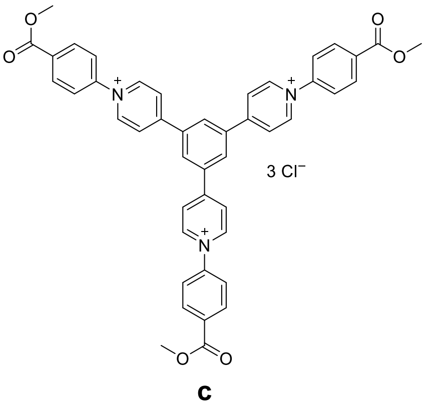

c

Compound c: In a 250 mL round-bottom flask, compound **b** (917 mg, 1.00 mmol) and methyl 4-aminobenzoate (2.16 g, 14.3 mmol) were dissolved in 72 mL of ethyl alcohol. The reaction mixture was refluxed for 5 days under a nitrogen atmosphere. The solvent was removed under reduced pressure, and the resulting solid was washed sequentially with acetone (100 mL) three times, followed by a wash with methanol (3 mL). The precipitate was collected and dried under vacuum to yield a white solid product. (610 mg, 74% yield). **^{1}H NMR** (CD_3OD, 298 K, 400 MHz): δ 9.49 (d, J = 7.0 Hz, 6H), 9.13 (s, 3H), 9.05 (d, J = 7.1 Hz, 6H), 8.42 (d, J = 8.7 Hz, 6H), 8.07 (d, J = 8.7 Hz, 6H), 4.01 (s, 9H). **^{13}C NMR** (CD_3OD, 298 K, 100 MHz): δ 165.4, 155.7, 145.6, 144.9, 137.0, 133.1, 131.7, 131.4, 126.1, 124.7, 51.9 ppm.

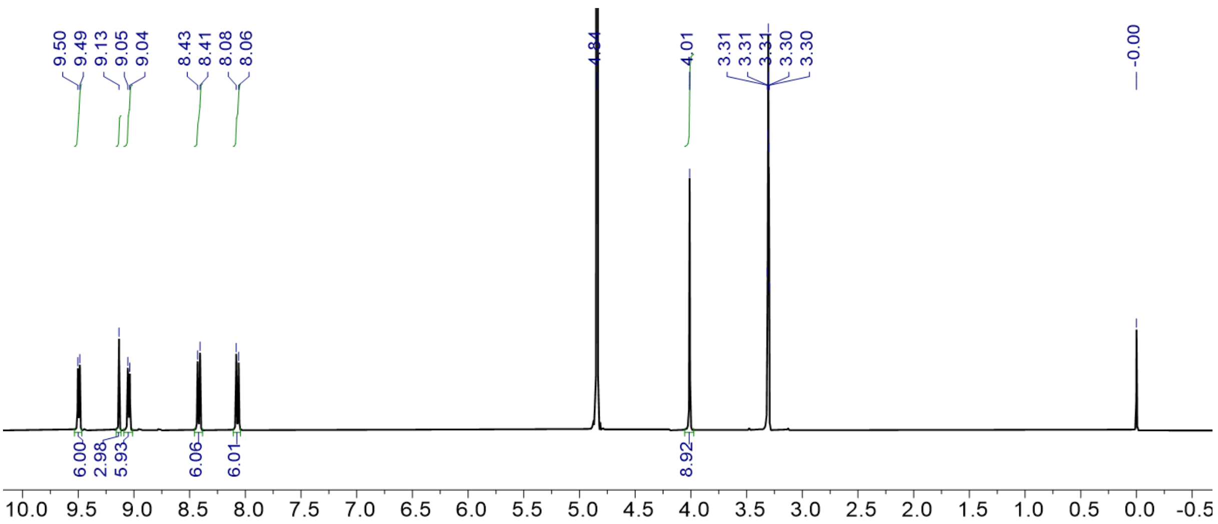

Figure S2. ^{1}H NMR spectrum (CD_3OD, 298 K, 400 MHz) of compound **c**.

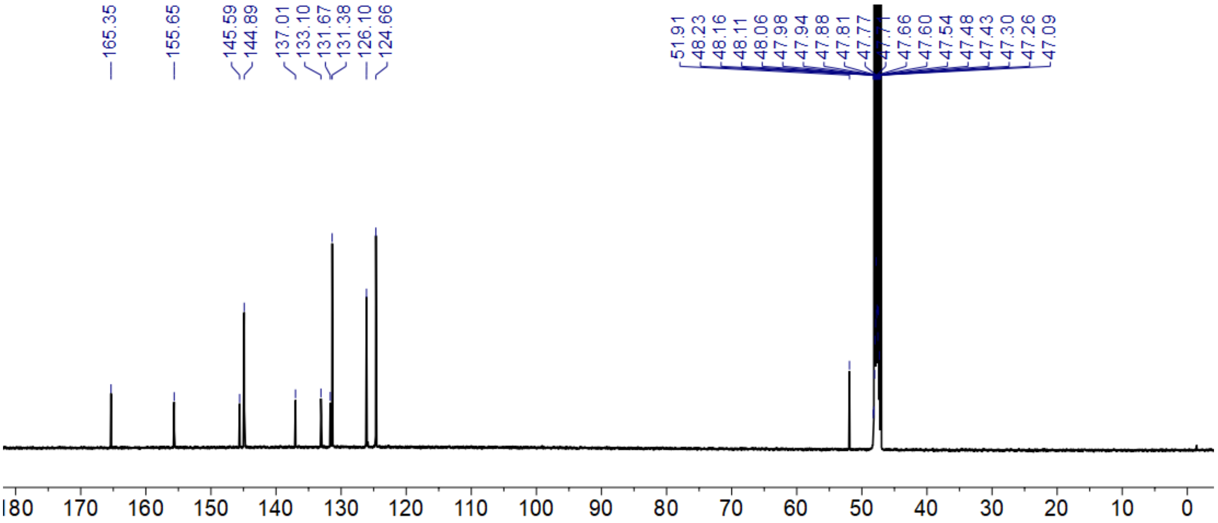

Figure S3. ^{13}C NMR spectrum (CD_3OD, 298 K, 100 MHz) of compound **c**.

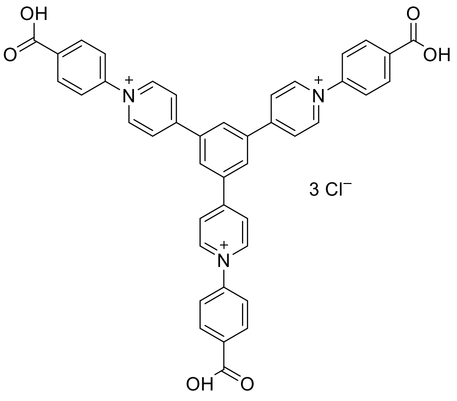

H_3**L**-Cl

H_3**L**-Cl**:** In a 250 mL round-bottom flask, compound **c** (400 mg, 0.487 mmol) was dissolved in 60 mL of water. A solution of HCl (36%, 60 mL) was then added to the flask. The reaction mixture was gradually heated to 100 °C and maintained overnight. Afterward, the solvent was removed under reduced pressure, yielding a crude product. The crude material was washed with 5 mL of methanol, resulting in the formation of a white solid (260 mg, 69% yield). **^{1}H NMR** (CD_3OD, 298 K, 300 MHz): δ 9.53 (d, J = 6.8 Hz, 6H), 9.15 (s, 3H), 9.06 (d, J = 6.8 Hz, 6H), 8.45 (d, J = 8.6 Hz, 6H), 8.08 (d, J = 8.6 Hz, 6H) ppm. **^{13}C NMR** (d_6-DMSO, 298 K, 100 MHz): δ 166.6, 154.6, 145.5, 145.5, 136.5, 133.7, 132.4, 131.4, 126.3, 125.9 ppm.

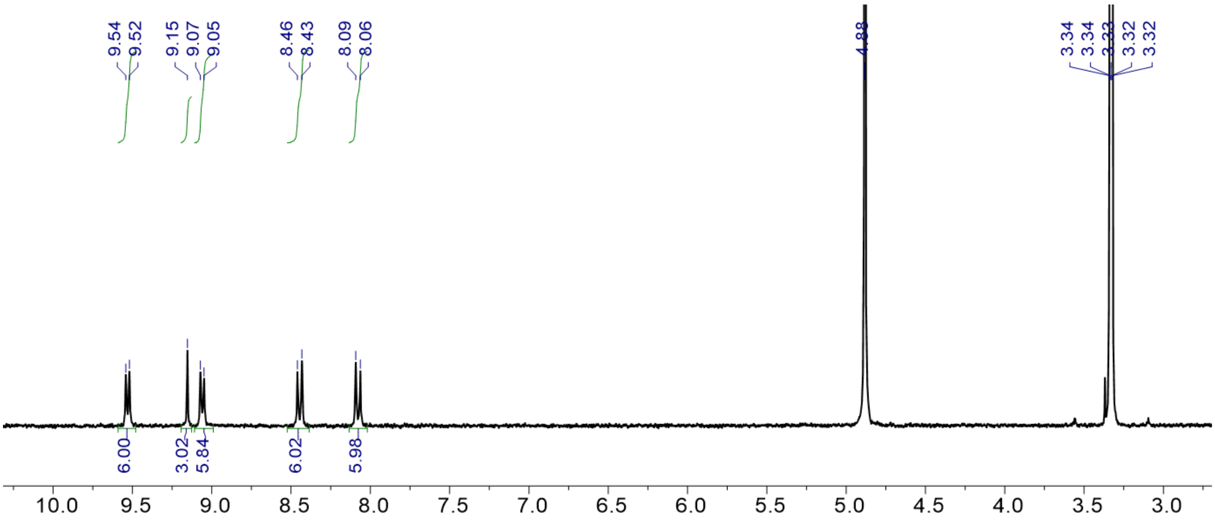

Figure S4. ^{1}H NMR spectrum (CD_3OD, 298 K, 300 MHz) of H_3**L**-Cl.

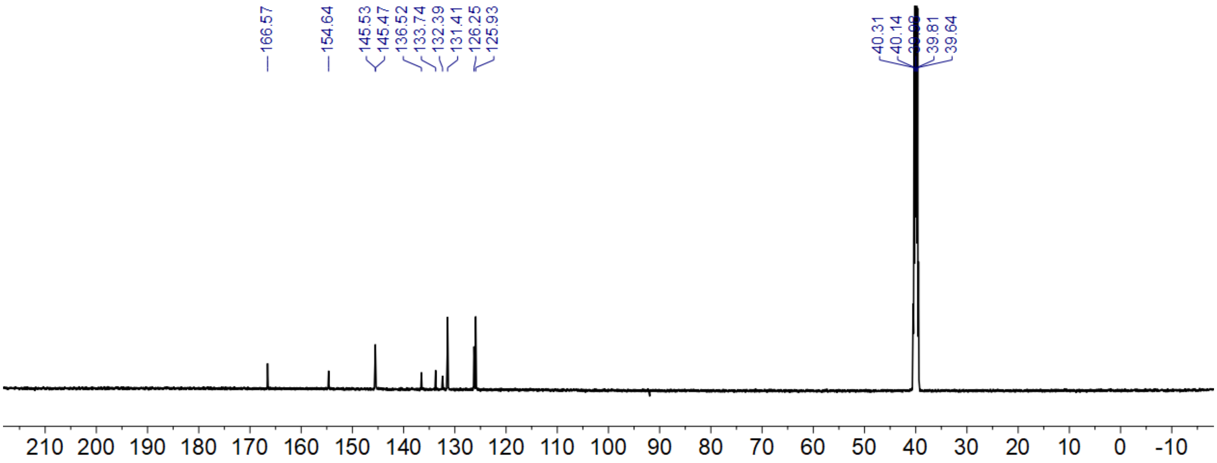

Figure S5. ^{13}C NMR spectrum (d_6-DMSO, 298 K, 100 MHz) of H_3**L**-Cl.

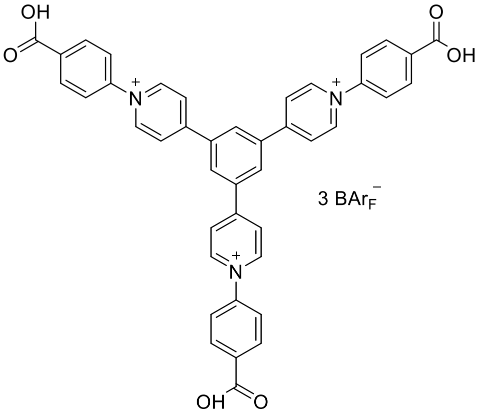

H_3**L**-BAr_F

H_3**L**-BAr_F was synthesized via an anion exchange of H_3**L**-Cl with NaBArF. H_3**L**-Cl (150 mg, 0.193 mmol) was dissolved in 150 mL of water and slowly heated to 70 °C to ensure complete dissolution. Subsequently, $NaBAr_F$ (512 mg, 0.578 mmol) was dissolved in 1 mL of ethanol and slowly added to the reaction mixture, resulting in the formation of a significant amount of white precipitate. After filtration, the precipitate was washed with water (3 × 15 mL), collected, and dried under vacuum to obtain a white solid. (500 mg, 80% yield). ^{1}**H NMR** (CD_3OD, 298 K, 400 MHz): δ 9.54 (d, J = 7.0 Hz, 6H), 9.10 (s, 4H), 8.98 (d, J = 7.0 Hz, 6H), 8.43 (d, J = 8.7 Hz, 7H), 8.02 (d, J = 8.7 Hz, 7H), 7.66–7.52 (m, 36H) ppm. ^{13}**C NMR** (CD_3OD, 298 K, 100 MHz): δ 166.5, 162.1, 161.7, 161.3, 160.9, 155.7, 145.2, 145.0, 137.1, 134.4, 131.7, 131.5, 129.4(3), 129.4(1), 129.2, 129.1(8), 129.1(6), 129.1(4), 128.9(5), 128.9(3), 128.9(1), 128.9, 128.7, 128.6, 127.6, 125.9, 125.5, 124.4, 123.3, 121.1, 117.2, 117.1, 117.0(8), 117.0(5), 117.0(2) ppm. ^{19}**F NMR** (CD_3OD, 298 K, 376 MHz): δ -64.3 ppm.

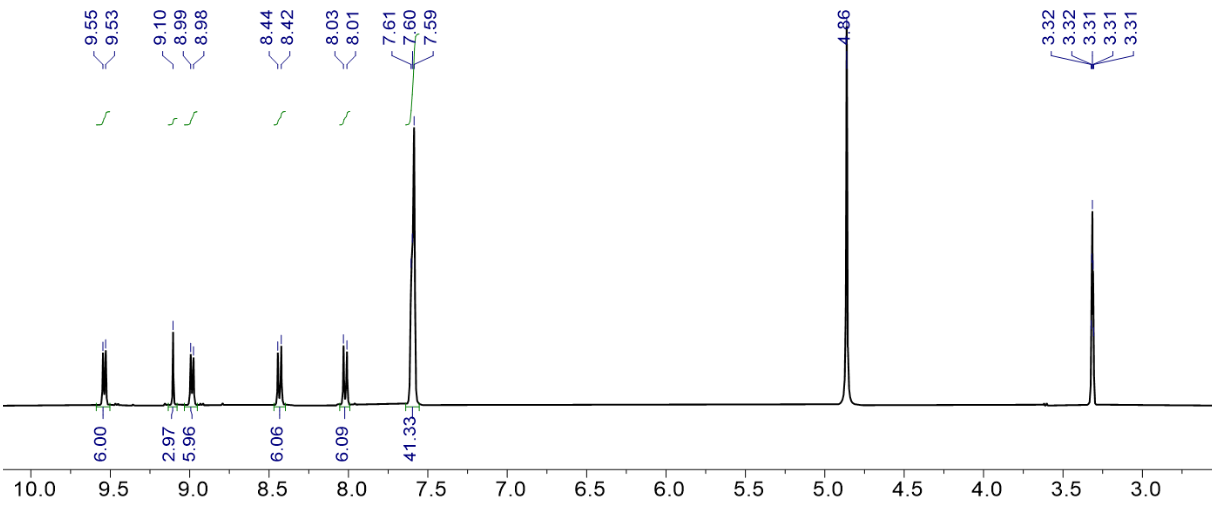

Figure S6. ^{1}H NMR spectrum (CD_3OD, 298 K, 400 MHz) of H_3**L**-BAr$_F$.

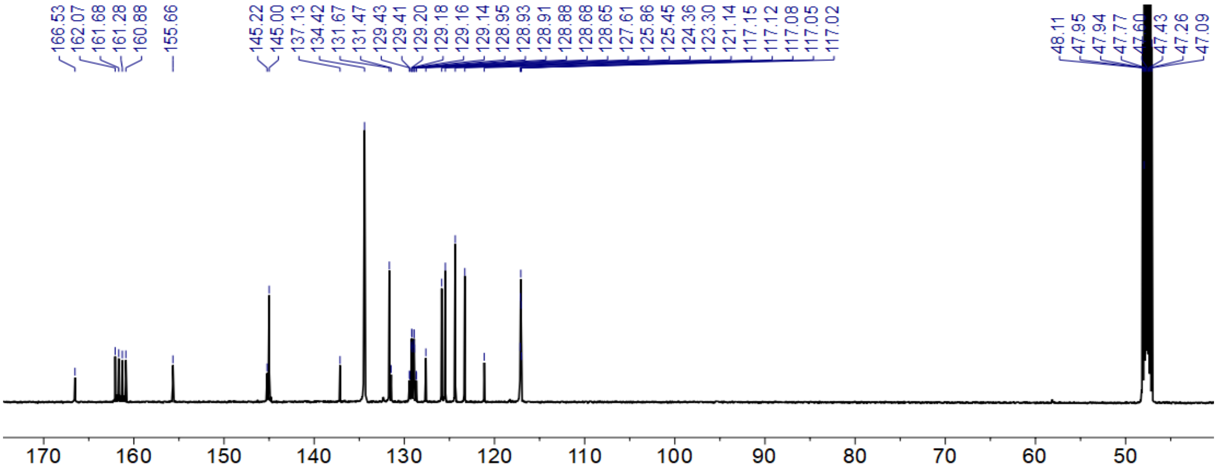

Figure S7. ^{13}C NMR spectrum (CD_3OD, 298 K, 100 MHz) of H_3**L**-BAr$_F$.

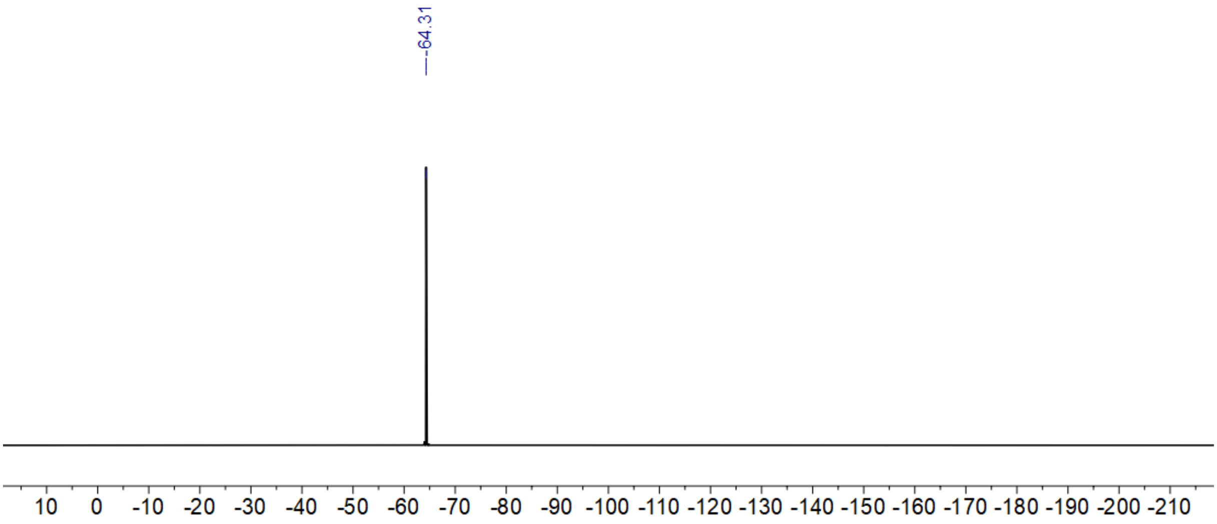

Figure S8. ^{19}F NMR spectrum (CD_3OD, 298 K, 376 MHz) of H_3**L**-BAr$_F$.

2.2 Synthesis and characterization of 1-BArF

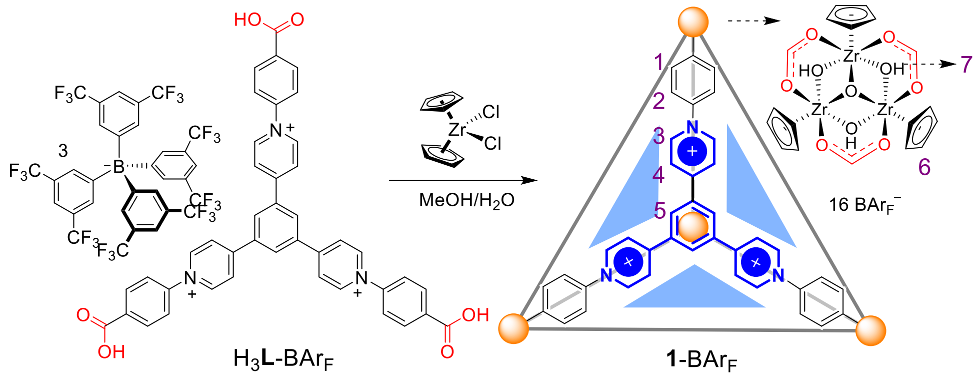

Figure S9. Synthesis of **1**-BArF.

H_3**L**-BArF (20 mg, 6.1 µmol, 1.0 equiv.), Cp_2ZrCl_2 (5.6 mg, 19 µmol, 3.1 equiv.), and NaBArF (5.4 mg, 6.1 µmol, 1.0 equiv.) were dissolved in a mixture of 3 mL of CH_3OH and 120 µL of H_2O. The reaction mixture was heated to 60 °C and stirred overnight. After cooling to room temperature, 12 mL of water was added, resulting in the formation of a large amount of white precipitate. The precipitate was collected by centrifugation, thoroughly washed with water (3 × 12 mL), and dried under vacuum to obtain **1**-BArF (21 mg, 74% yield). As the ^{1}H NMR signals of **1**-BArF are broad presumably due to the partial dissociation of the cage, the subsequent NMR characterization was performed after adding 32 equiv. TBAOTf as the template. **^{1}H NMR** (CD_3OD, 298 K, 400 MHz): δ 9.30 (d, J = 6.6 Hz, 24H), 8.73 (d, J = 6.6 Hz, 24H), 8.64 (s, 12H), 8.39 (d, J = 8.5 Hz, 24H), 7.95 (d, J = 8.5 Hz, 24H), 7.68 – 7.52 (m, 265H), 6.75 (s, 61H) ppm. **^{13}C NMR** (CD_3OD, 298 K, 100 MHz): δ 173.0, 162.1, 161.9, 161.8, 161.7, 161.6, 161.5, 161.4, 161.3, 161.2, 161.1, 160.9, 156.3, 145.8, 144.3, 137.6, 134.4, 134.0, 132.1, 131.1, 129.5, 129.4(4), 129.4(1), 129.4(0), 129.2, 129.1(8), 129.1(6), 129.1(4), 129.0, 128.9(3), 128.9(1), 128.9(0), 128.7, 128.6(8), 128.6(6), 128.6(3), 127.6, 126.4, 125.5, 124.2, 123.3, 121.2, 117.2, 117.1, 117.0(9), 117.0(6), 117.0(3), 116.5 ppm. **^{19}F NMR** (CD_3OD, 298 K, 376 MHz): δ -65.7 ppm.

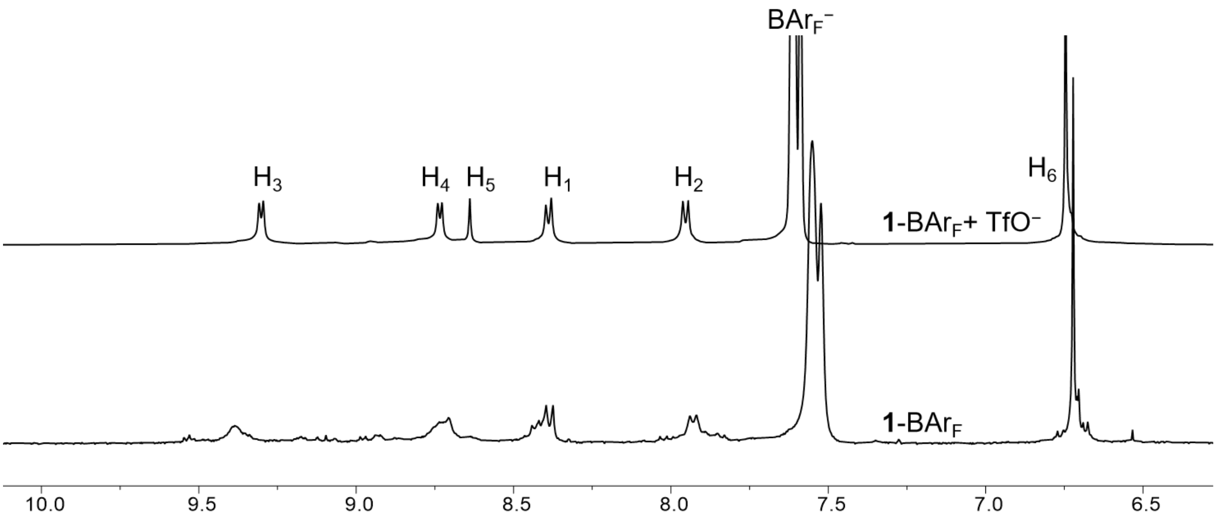

Figure S10. ^{1}H NMR spectra (CD_3OD, 298 K, 400 MHz) of **1**-BArF in the absence and presence of 32 equiv. TBAOTf.

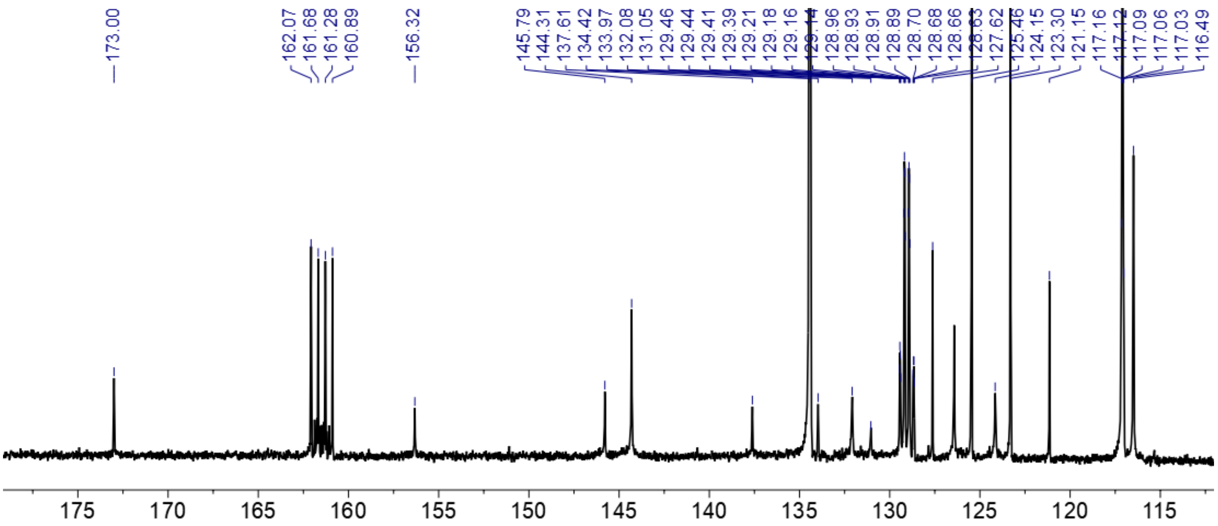

Figure S11. ^{13}C NMR spectrum (CD_3OD, 298 K, 100 MHz) of **1**-BAr_F in the absence and presence of 32 equiv. TBAOTf.

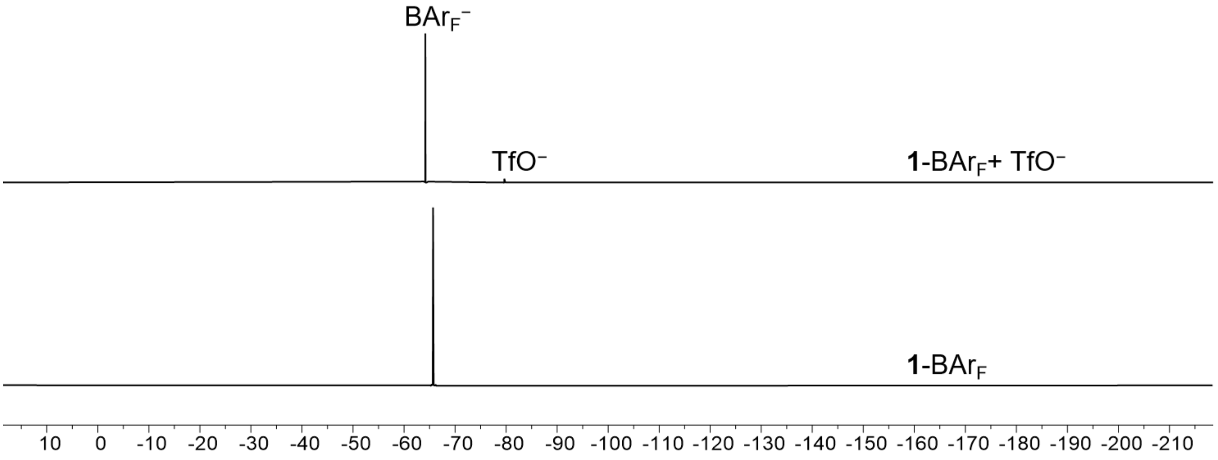

Figure S12. ^{19}F NMR spectra (CD_3OD, 298 K, 376 MHz) of **1**-BAr_F in the absence and presence of 32 equiv. TBAOTf.

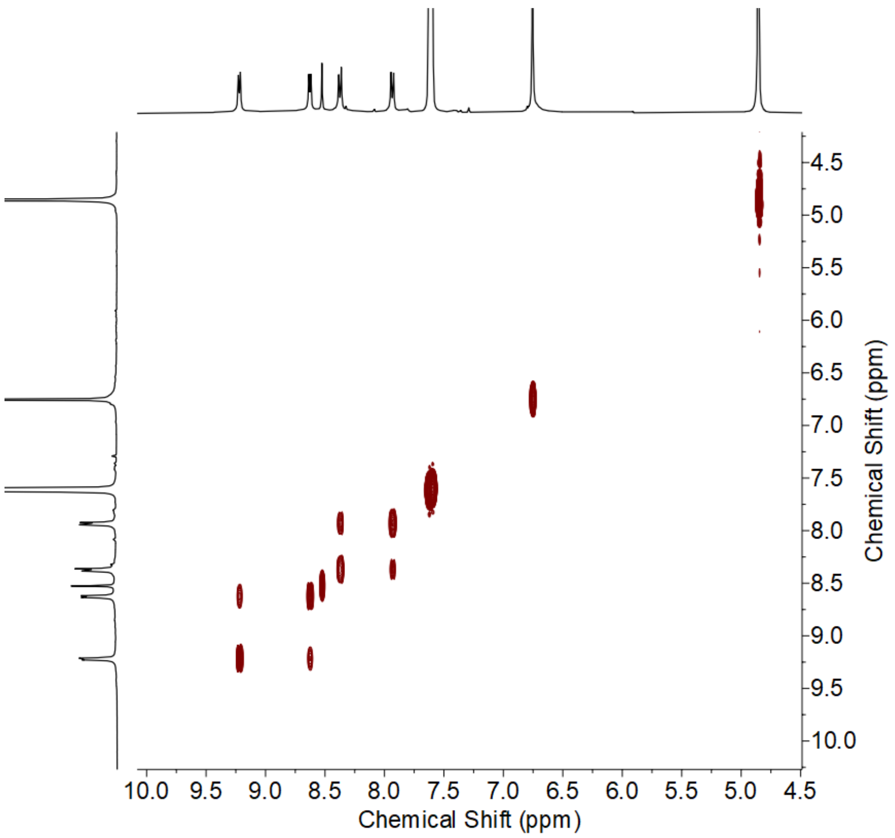

Figure S13. ^1H-^1H COSY spectrum (CD_3OD, 298 K, 500 MHz) of **1**-BAr_F in the presence of 32 equiv. TBAOTf.

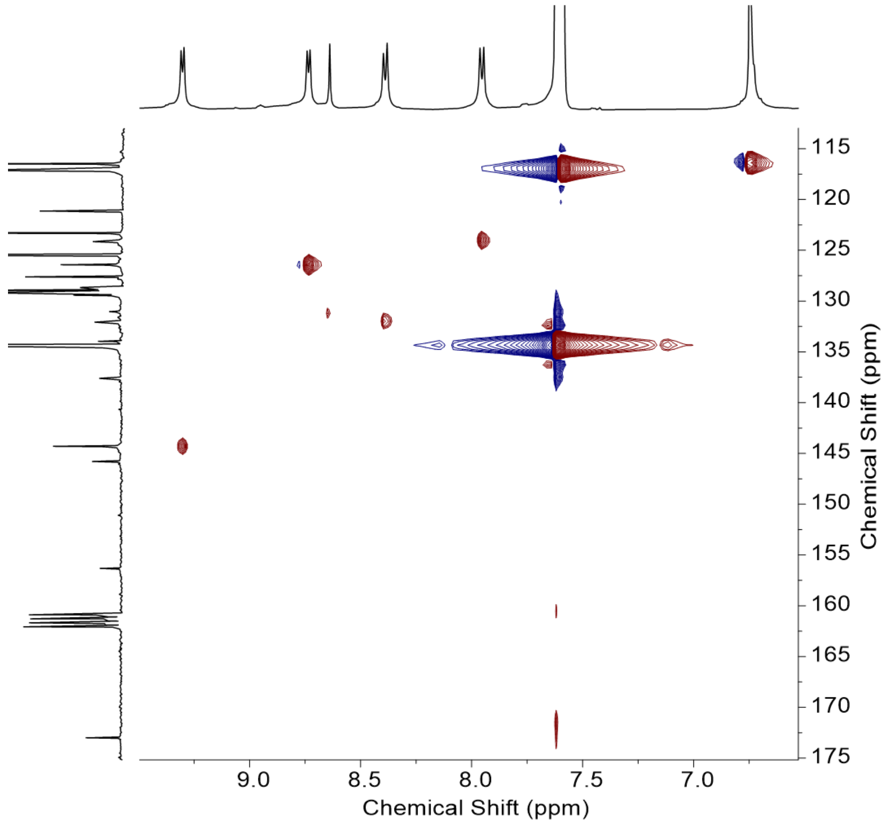

Figure S14. ^1H-^{13}C HSQC spectrum (CD_3OD, 298 K, 500 MHz) of **1**-BAr_F in the presence of 32 equiv. TBAOTf.

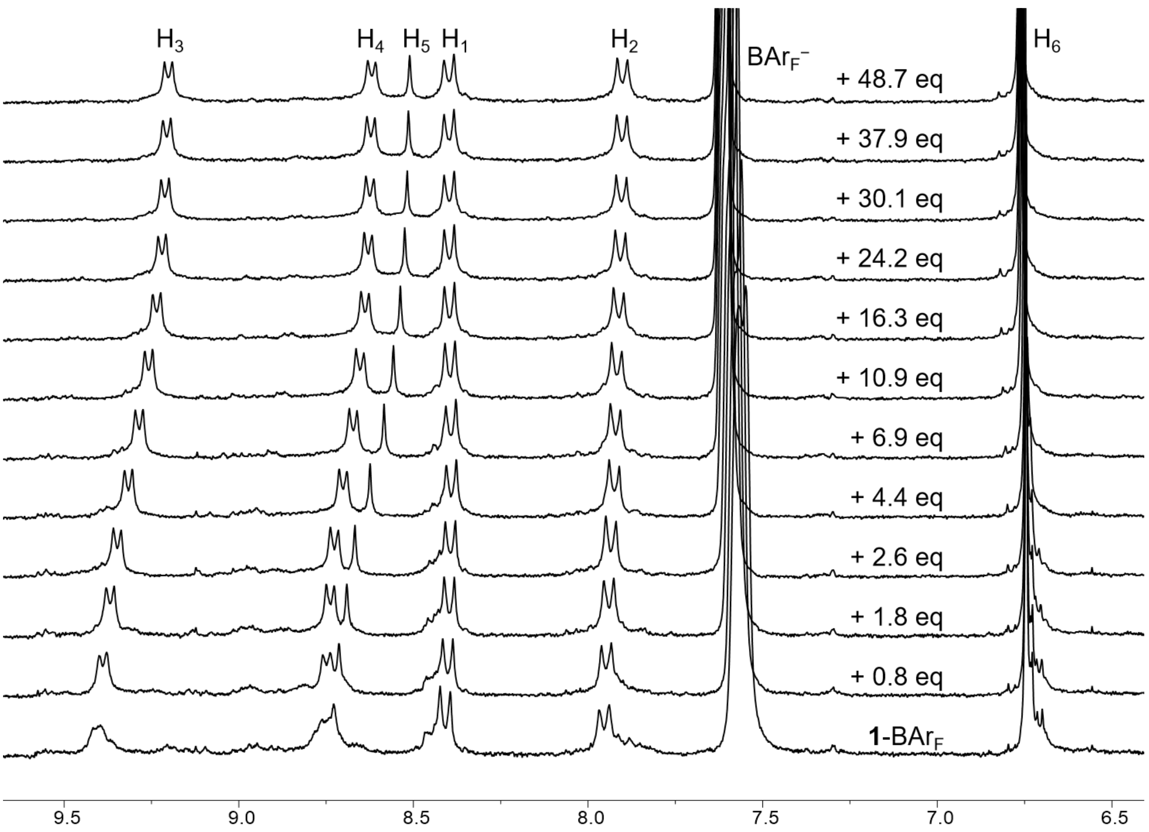

Figure S15. ^{1}H NMR (CD_3OD, 298 K, 300 MHz) titrations of $TBANTf_2$ into a methanol solution of **1**-BAr_F (0.25 mM).

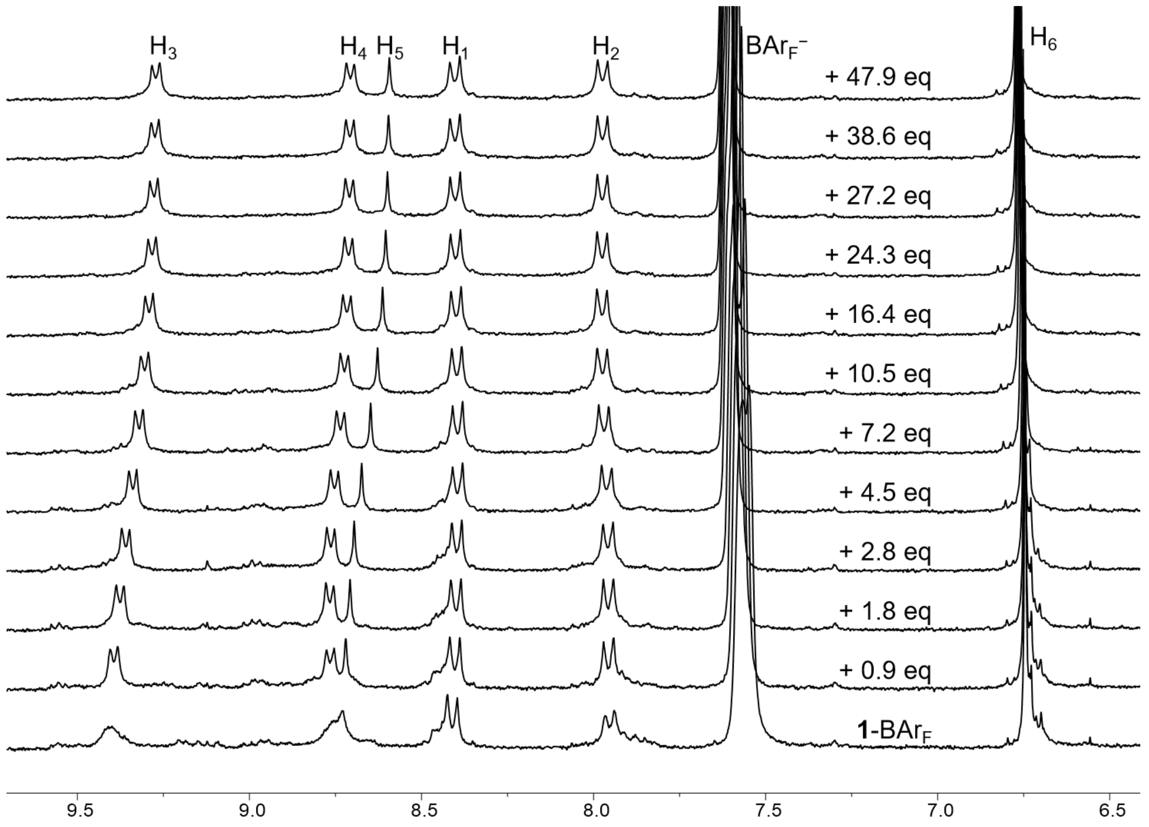

Figure S16. ^{1}H NMR (CD_3OD, 298 K, 300 MHz) titrations of TBAOTf into a methanol solution of **1**-BAr_F (0.25 mM).

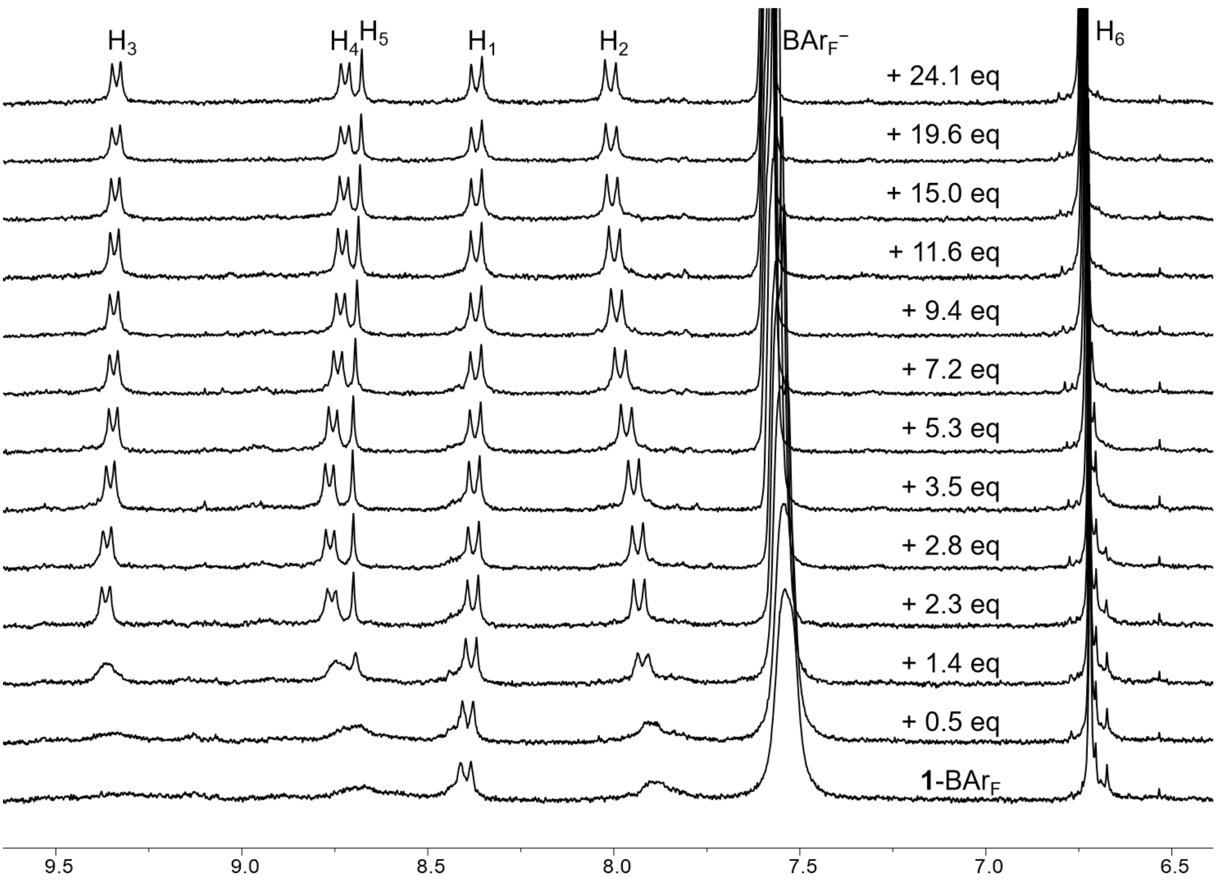

Figure S17. ^{1}H NMR (CD_3OD, 298 K, 300 MHz) titrations of $TBANO_3$ into a methanol solution of **1**-BAr_F (0.25 mM).

2.3 Synthesis and characterization of 1-X (X = OTf, NTf_2, NO_3)

Synthesis of 1-OTf: 1-BAr_F (101 mg, 5.42 µmol, 1.00 equiv.) was dissolved in 2 mL of CH_3OH, followed by the addition of TBAOTf (80.0 mg, 205 µmol, 37.8 equiv.). Upon the subsequent introduction of 13 mL of diethyl ether, a significant amount of precipitate formed immediately, which was isolated through centrifugation. The solid thus obtained still contains a minor amount of BAr_F^- in addition to TfO^- as the counteranions. To further minimize the BAr_F^- content, the solid was dissolved in 3 mL CH_3OH, and diethyl ether (12 mL) was added again to the solution to precipitate **1**-OTf. The precipitate was collected and was dried under vacuum to obtain **1**-OTf (32 mg, 81% yield). **^{1}H NMR** (CD_3OD, 298 K, 300 MHz) δ 9.26 (d, *J* = 6.5 Hz, 24H), 8.69 (d, *J* = 6.5 Hz, 24H), 8.58 (s, 12H), 8.38 (d, *J* = 8.3 Hz, 24H), 7.96 (d, *J* = 8.5 Hz, 24H), 6.75 (s, 60H) ppm. **^{19}F NMR** (CD_3OD, 298 K, 376 MHz): *δ* -80.0 ppm.

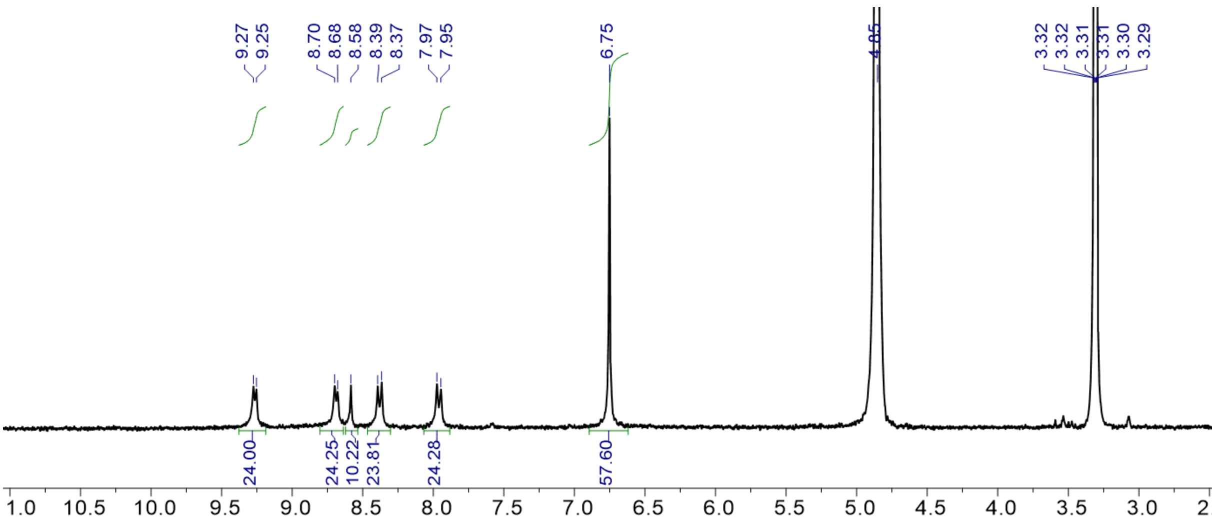

Figure S18. ^{1}H NMR spectrum (CD_3OD, 298 K, 300 MHz) of **1**-OTf.

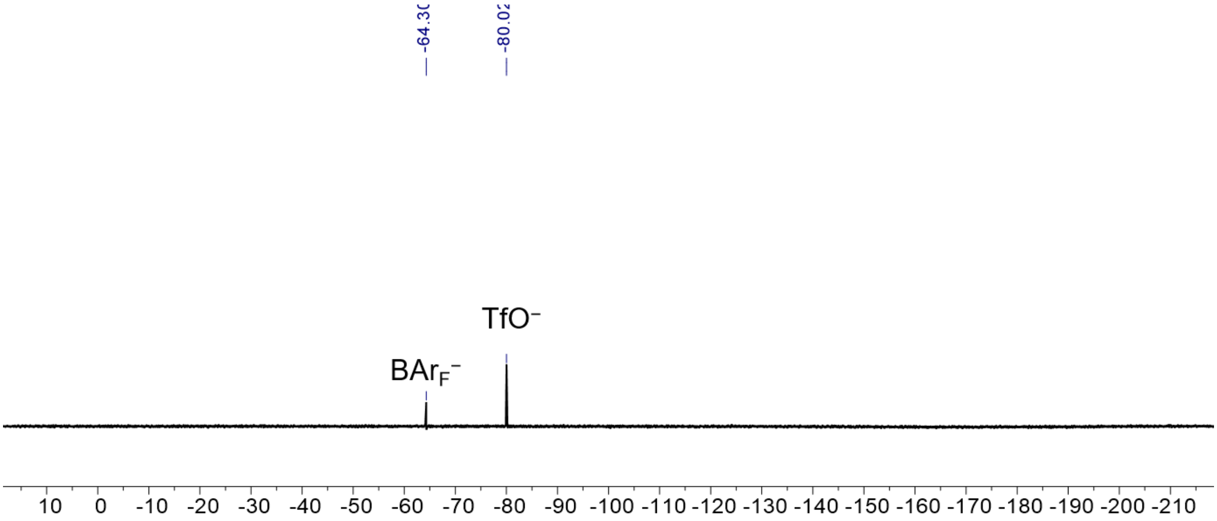

Figure S19. ^{19}F NMR spectrum (CD_3OD, 298 K, 376 MHz) of **1**-OTf. A trace amount of BAr_F^- (0.016 equiv.) relative to OTf^- was present.

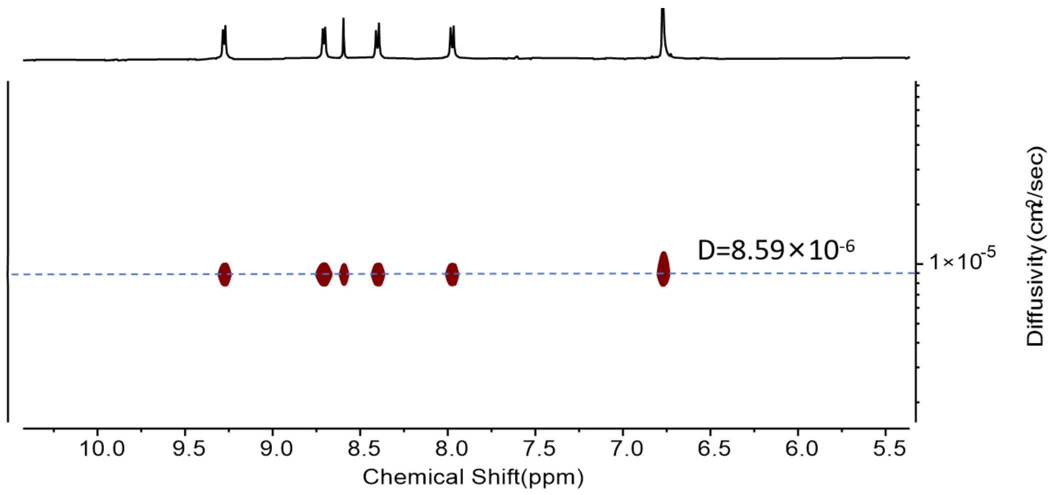

Figure S20. DOSY spectrum (CD_3OD, 298 K, 500 MHz) of **1**-OTf.

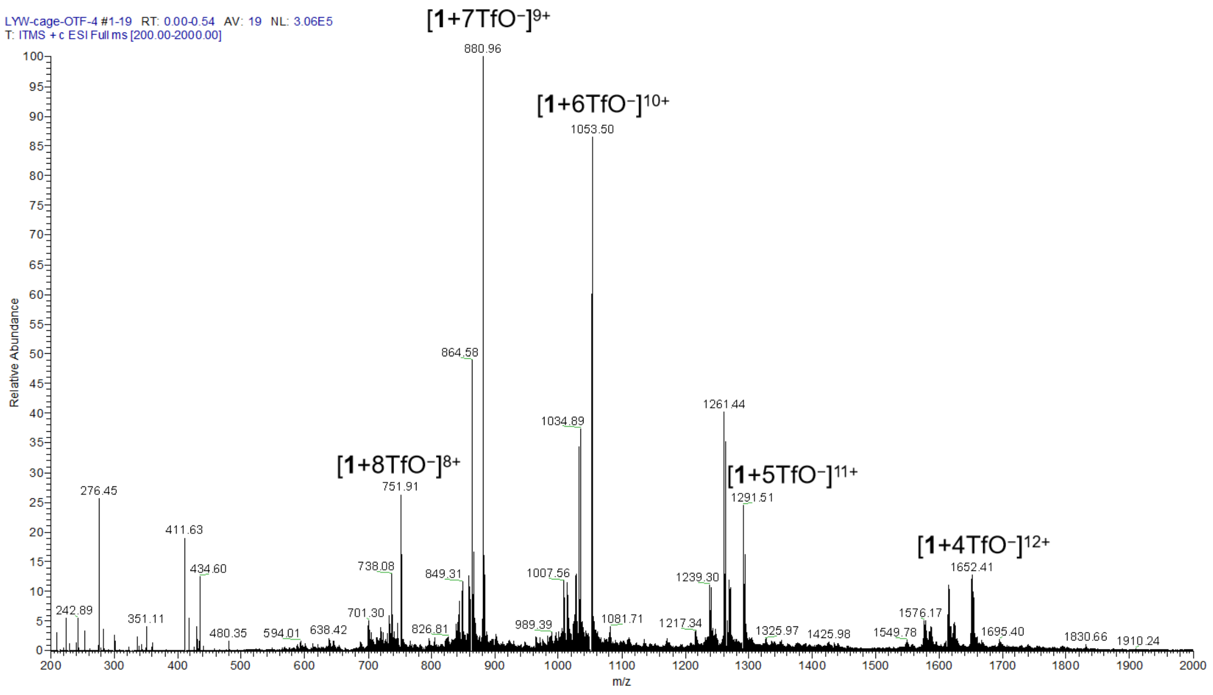

Figure S21. Low-resolution ESI-mass spectrum of **1**-OTf.

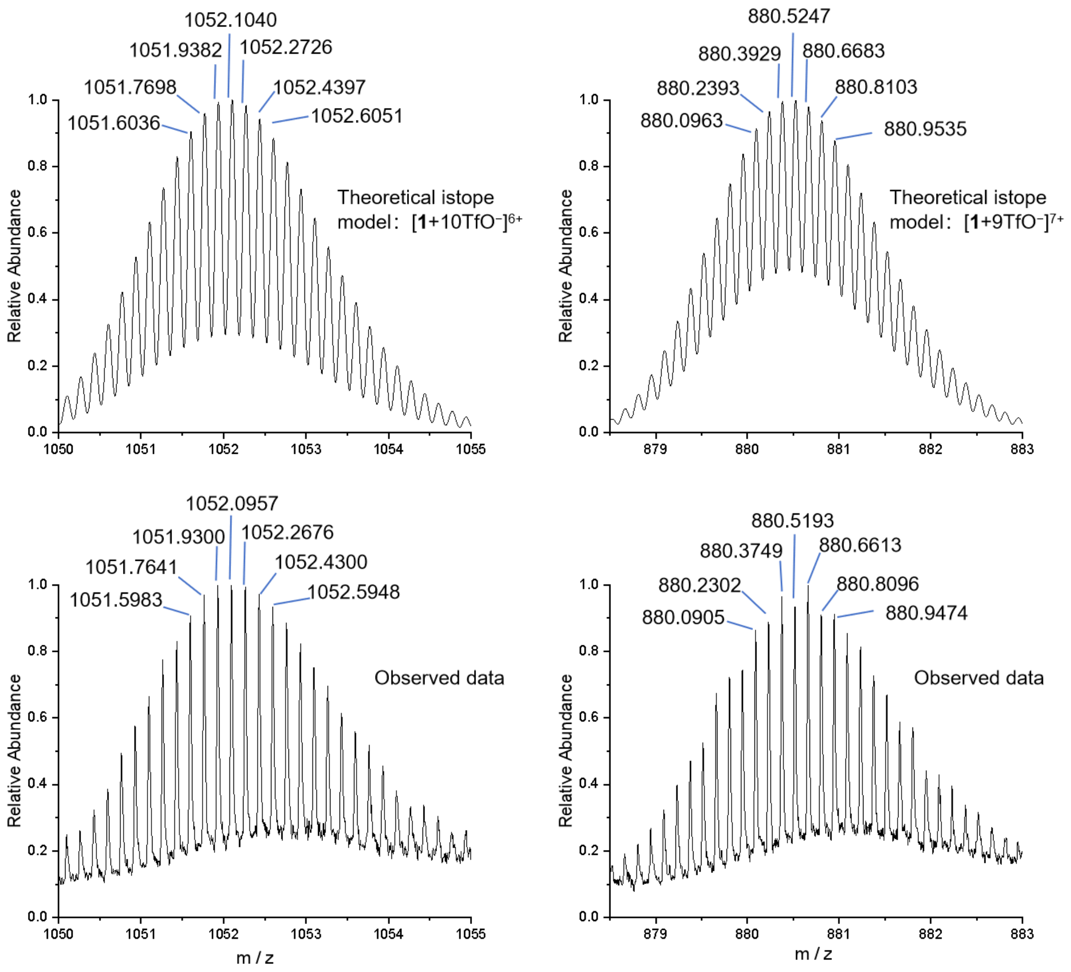

Figure S22. Simulated (top) and experimental (bottom) high-resolution ESI-mass spectra of **1**-OTf showing the 6+ and 7+ peaks.

Synthesis of 1-NTf$_2$: 101 mg (5.42 μmol, 1.00 equiv.) of **1**-BAr_F was dissolved in 2 mL of CH_3OH, and $TBANTf_2$ (104 mg 199 μmol, 36.7 equiv.) was added. Upon the subsequent introduction of 13 mL of diethyl ether, a significant amount of precipitate formed immediately, which was isolated through centrifugation. The solid thus obtained still contains a minor amount of BAr_F^- in addition to Tf_2N^-. To minimize the BAr_F^- content, the solid was dissolved in 3 mL CH_3OH, and diethyl ether (12 mL) was added again to the solution to precipitate **1**-NTf_2. The precipitate was collected and dried under vacuum to obtain **1**-NTf_2 (31 mg, 62% yield). **^{1}H NMR** (CD_3OD, 298 K, 400 MHz): δ 9.17 (d, *J* = 6.6 Hz, 24H), 8.59 (d, *J* = 6.6 Hz, 24H), 8.47 (s, 12H), 8.37 (d, *J* = 8.5 Hz, 24H), 7.87 (d, *J* = 8.4 Hz, 24H), 6.73 (s, 60H) ppm. **^{19}F NMR** (CD_3OD, 298 K, 376 MHz): *δ* -79.8 ppm.

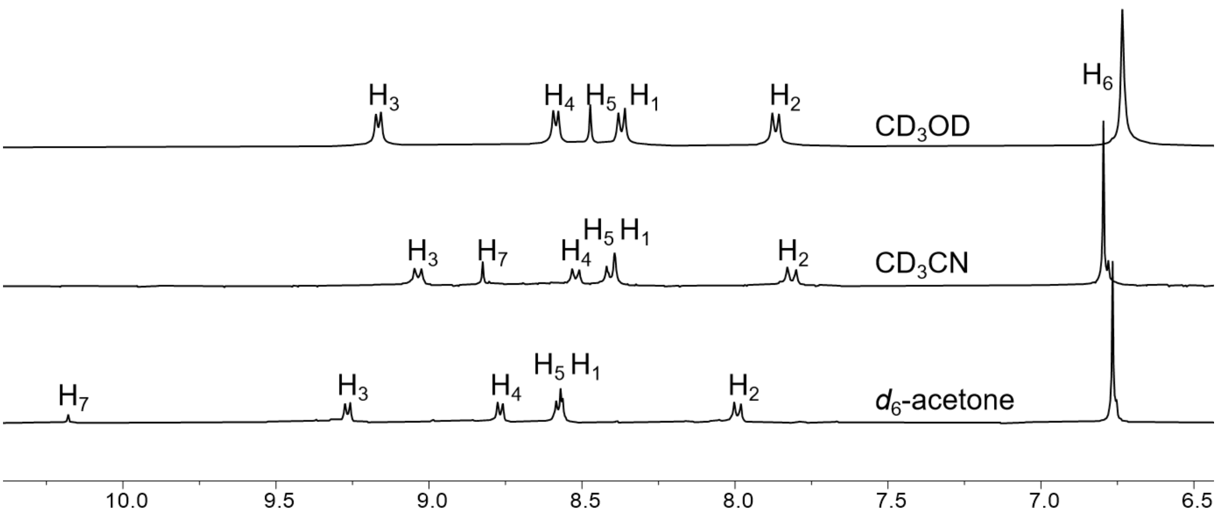

Figure S23. ^{1}H NMR spectra (298 K, 400 MHz) of **1**-NTf_2 in various solvents.

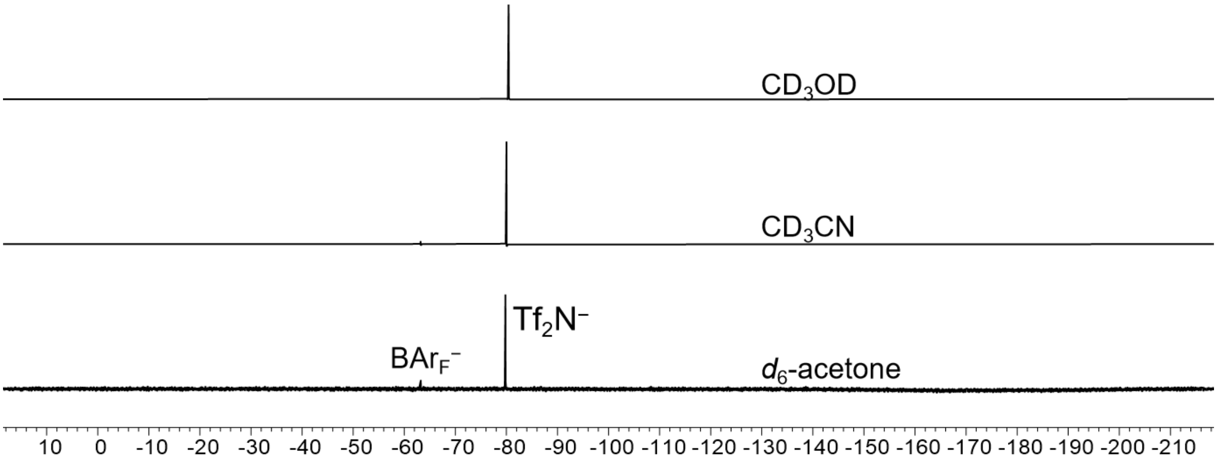

Figure S24. ^{19}F NMR spectra (298 K, 376 MHz) of **1**-NTf_2 in various solvents**.** A trace amount of BAr_F^- (0.011 equiv.) relative to Tf_2N^- was present.

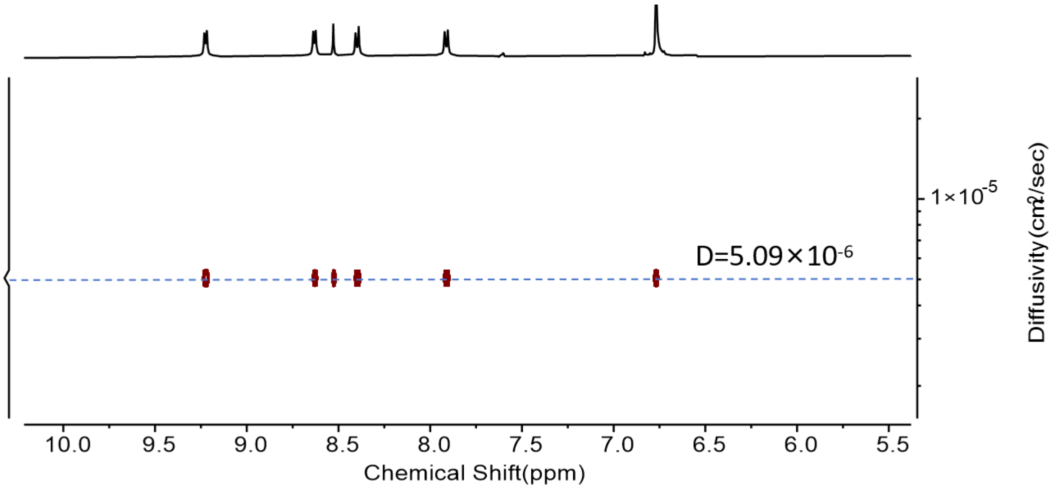

Figure S25. DOSY spectrum (CD_3OD, 298 K, 500 MHz) of **1**-NTf_2.

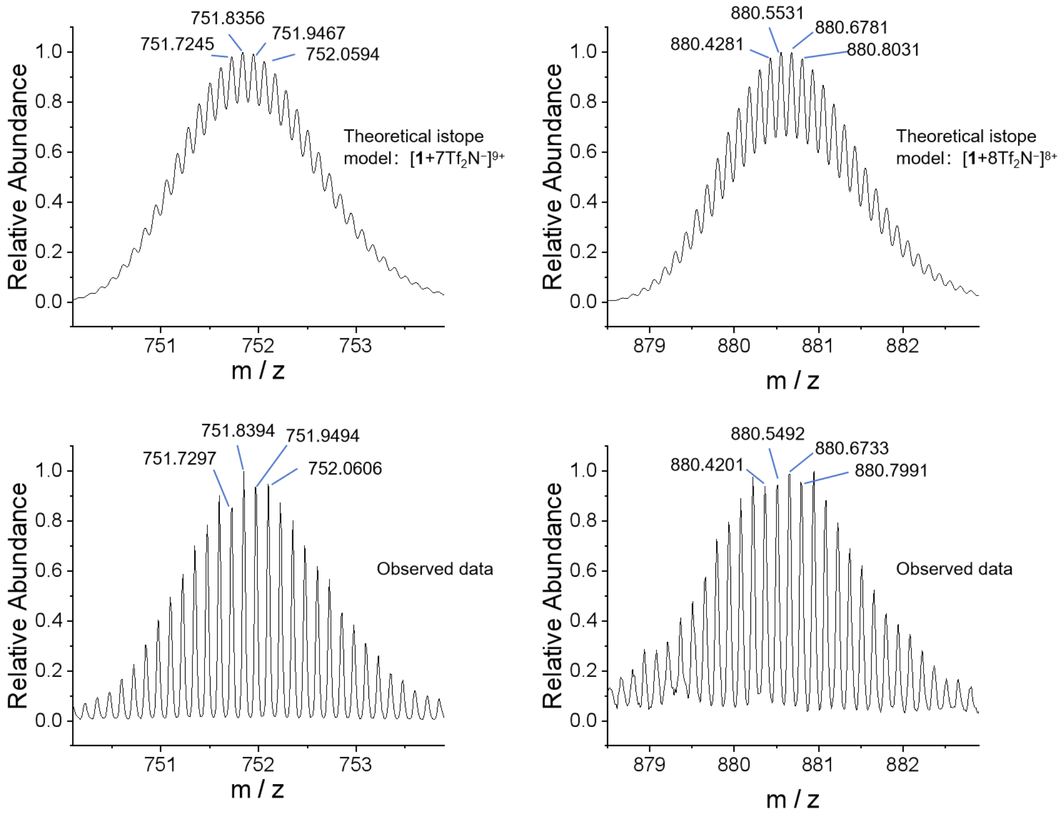

Figure S26. Simulated (top) and experimental (bottom) high-resolution ESI-mass spectra of **1**-NTf_2 showing the 8+ and 9+ peaks.

Synthesis of 1-NO_3: **1**-BAr_F (101 mg, 5.42 µmol, 1.00 equiv.) was dissolved in 2.0 mL CH_3OH and $TBANO_3$ (50.0 mg, 164 µmol, 30.3 equiv.) was added. A large amount of white precipitate appeared immediately. After filtration and washing with CH_3OH (3 × 10 mL), the precipitate was collected and was dried under vacuum to obtain **1**-NO_3 (25 mg, 80% yield). Despite the hydrophilic nature of NO_3^-, **1**-NO_3 was found to be insoluble in water, but soluble in DMSO. **^{1}H NMR** (d_6-DMSO, 298 K, 400 MHz): δ 10.75 (s, 12H), 9.71 (d, J = 6.0 Hz, 24H), 8.83 (d, J = 6.5 Hz, 24H), 8.72 (s, 12H), 8.30 (d, J = 8.3 Hz, 24H), 8.06 (d, J = 8.5 Hz, 24H), 6.76 (s, 60H) ppm.

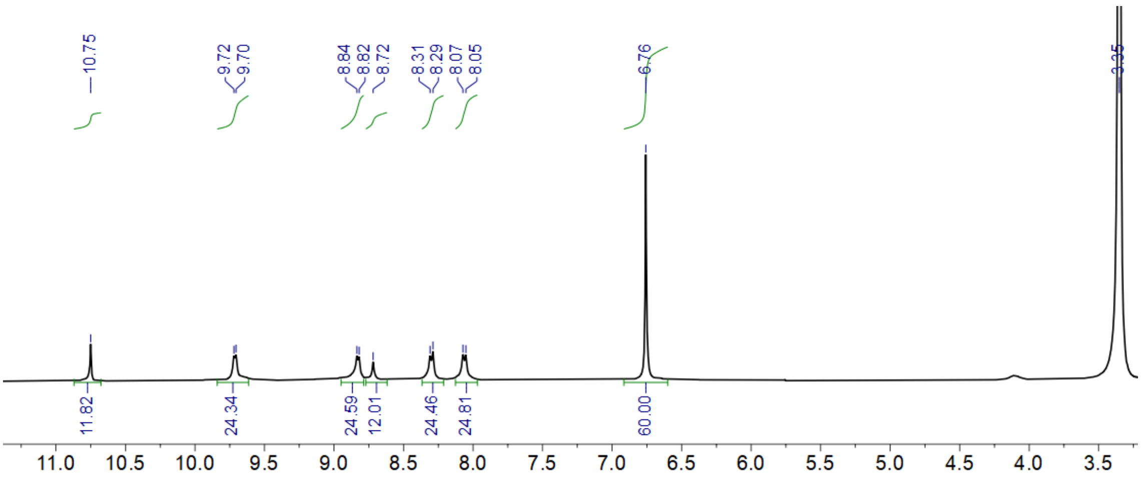

Figure S27. ^{1}H NMR spectrum (d_6-DMSO, 298 K, 400 MHz) of **1**-NO_3.

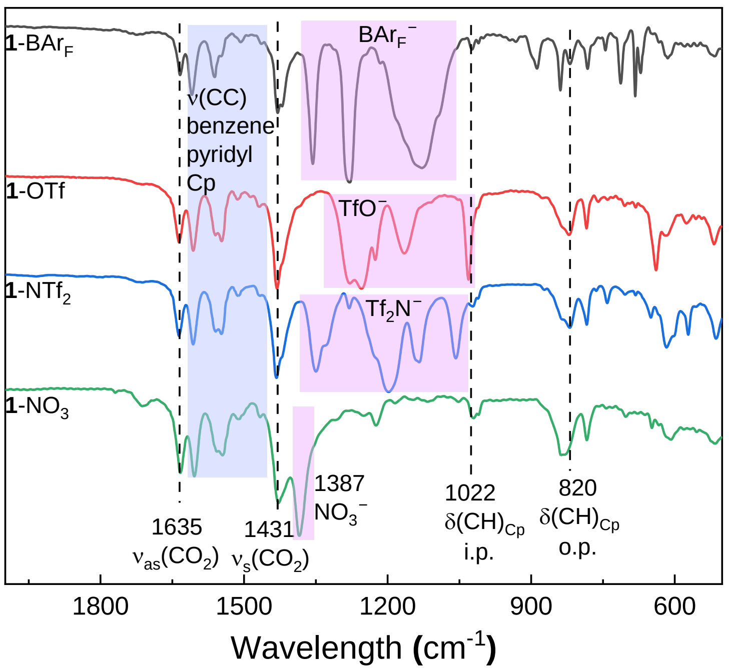

Figure S28. FT-IR spectra of **1**-BAr$_F$, **1**-OTf, **1**-NTf$_2$ and **1**-NO_3. The peaks observed at 1635 and 1431 cm^{-1} are owing to the asymmetric (ν_{as}) and symmetric (ν_s) stretching vibrations, respectively, of the carboxylate groups. The peaks in the range of 1615 –1450 cm^{-1} (blue region) are attributable to the skeletal vibrations of the benzene ring, pyridyl and cyclopentadienyl (Cp) groups. The purple region reveals the presence of various anions (BAr$_F^-$, Tf$_2$N$^-$ TfO$^-$ and NO_3^-). Additionally, the in-plane (i.p.) and out-of-plane (o.p.) deformation vibrations of the Cp moiety appears as weak bands at 1022 cm^{-1} and 820 cm^{-1}, respectively,[2] which may overlap with the absorption of the anions.

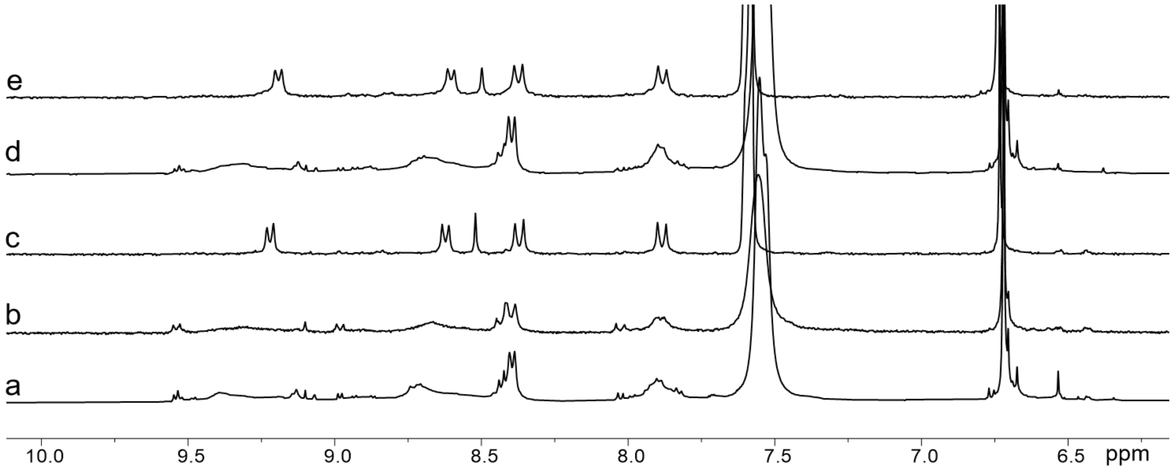

Figure S29. ^{1}H NMR spectra (298 K) of **1**-BAr$_F$ in CD_3OD. (a) The fresh solution. (b) The solution kept under ambient conditions for one month. (c) Adding TBANTf$_2$ (30 eq) into the solution after measuring (b). (d) The solution prepared using the solid of **1-** BAr$_F$ kept under ambient conditions. (e) Adding TBANTf$_2$ (30 eq) into the solution after measuring (d).

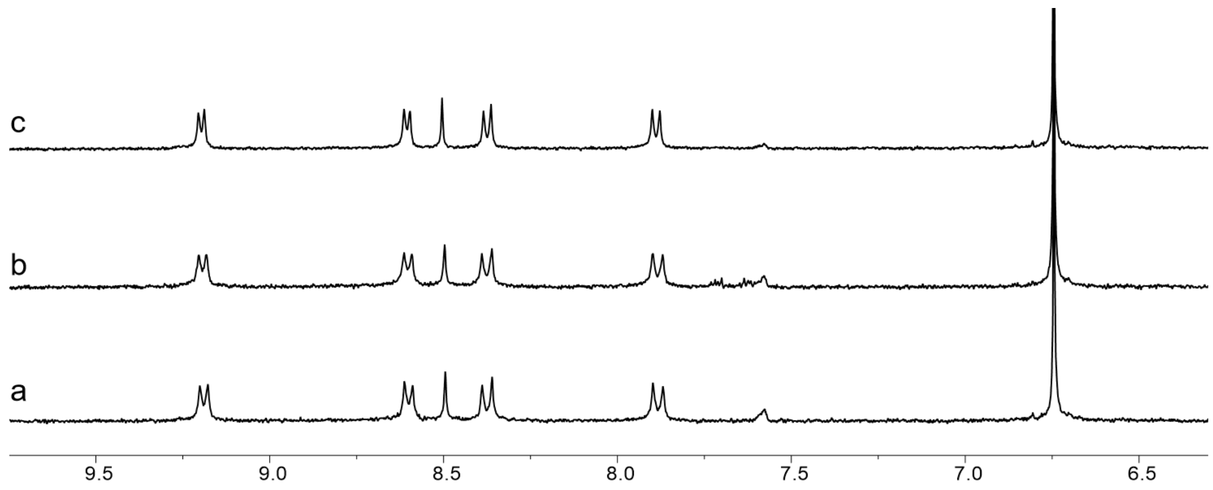

Figure S30. ^{1}H NMR spectra (CD_3OD, 298 K) of **1**-NTf_2 (a), **1**-NTf_2 kept in CD_3OD for one month (b), and **1**-NTf_2 kept as solid for one month (c).

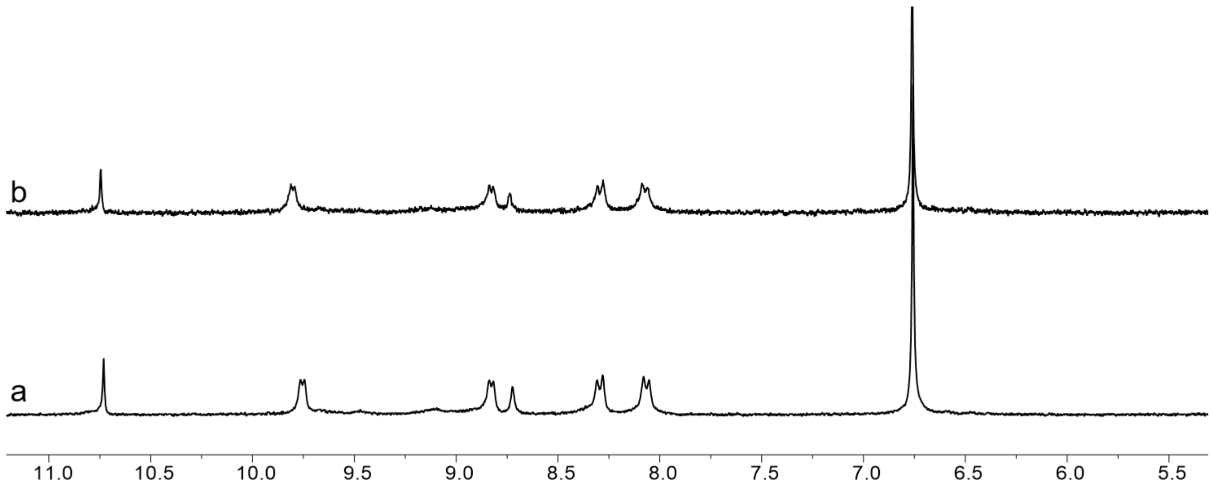

Figure S31. ^{1}H NMR spectra (d_6-DMSO, 298 K) of a fresh sample of **1**-NO_3 (a) and the sample kept as solid for one month (b).

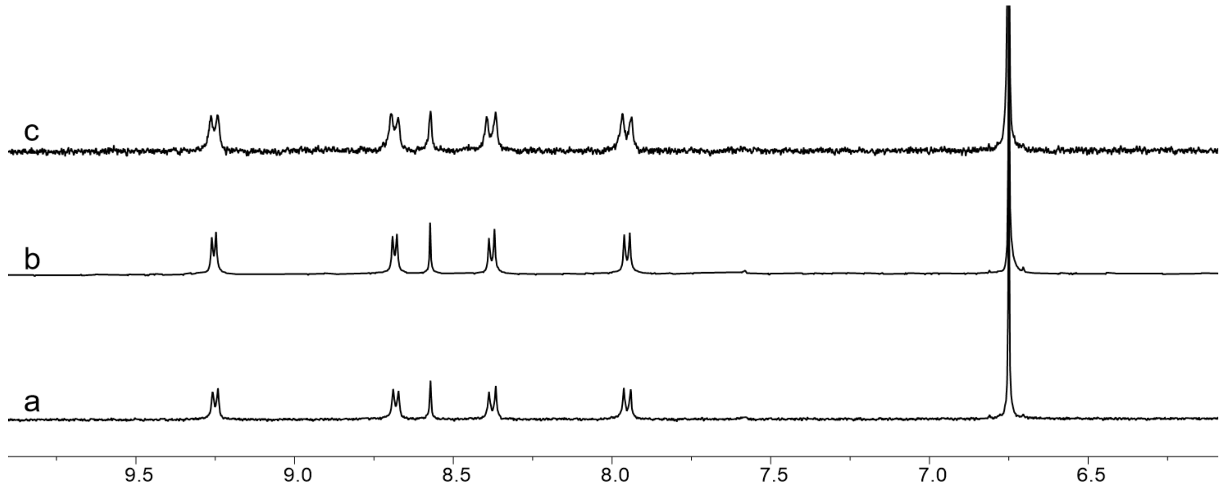

Figure S32. ^{1}H NMR spectra (CD_3OD, 298 K) of **1**-OTf(a), **1**-OTf kept in CD_3OD for one month (b), and **1**-OTf kept as solid for one month (c).

3. Guest binding studies

^{1}H NMR titrations were carried out by incrementally adding the methanol solution of the sulfonate guests into a methanol solution of **1**-BAr_F (0.25 mM). The mixture was immediately subjected to ^{1}H NMR. In case of fast-exchange binding, the binding constants were determined using BINDFIT[3] with the data obtained from ^{1}H NMR titrations.

In case of slow-exchange binding, the addition of a guest into **1**-BAr_F established the following equilibrium:

$$H + G \rightleftarrows HG$$

The concentration of free cage [H], free guest [G], and the host-guest complex [HG] were determined from integration of their NMR peaks relative to those of 1,3,5-trimethoxybenzene (internal standard). The binding constant for a 1:1 binding system was calculated using the following equation:

$$K_a = \frac{[HG]}{[H][G]}$$

The stepwise binding constants K_{ai} (i = 1, 2… n) for a system in which n (≥ 2) guests bind to the host were calculated using the following equation:

$$K_{ai} = \frac{[HG_i]}{[HG_{i-1}][G]}$$

The concentrations involved here were determined from integration of their NMR peaks relative to those of the 1,3,5-trimethoxybenzene internal standard.

3.1 ^{1}H NMR titrations of monosulfonates

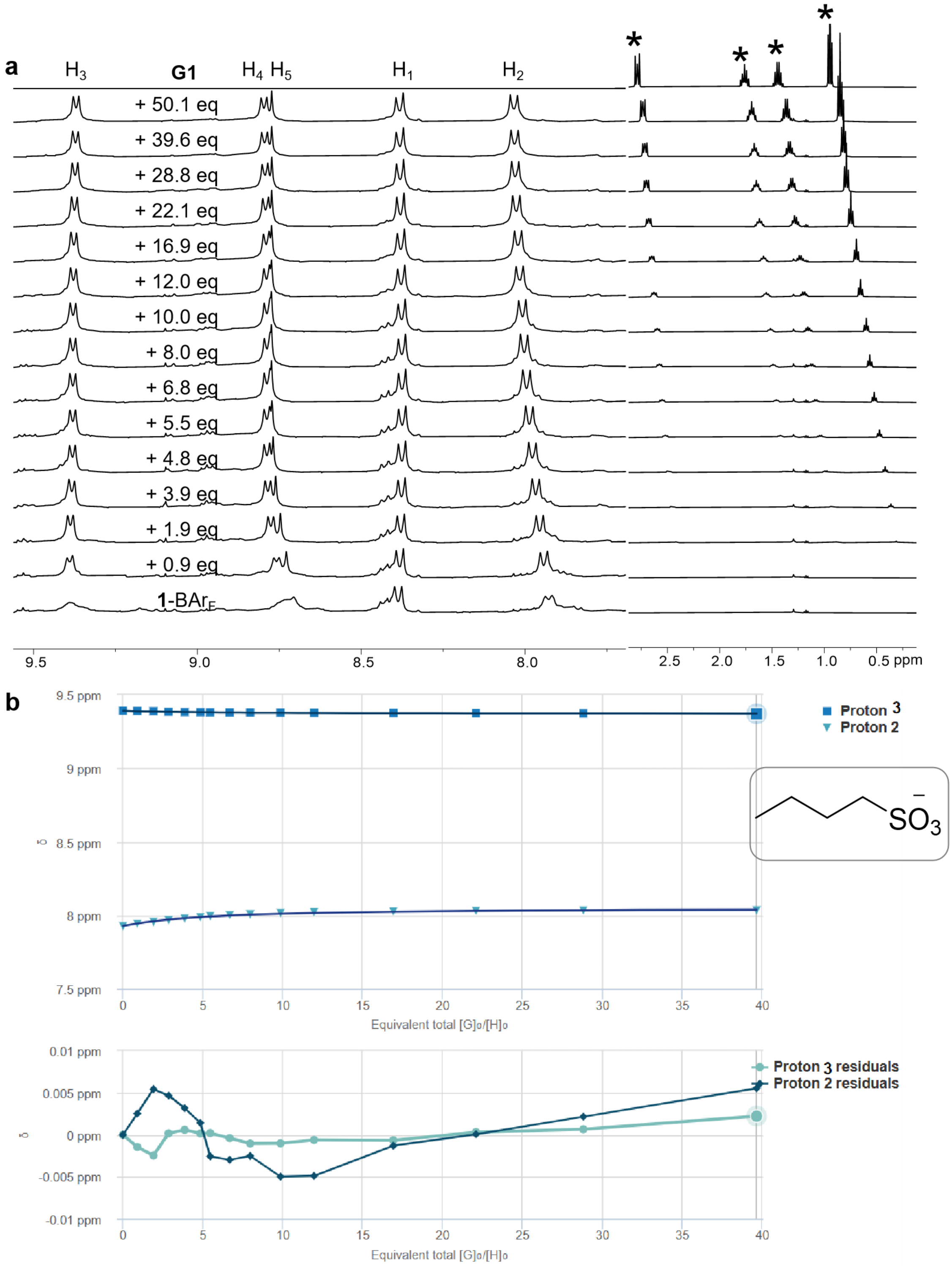

Figure S33. ^{1}H NMR (CD_3OD, 298 K, 400 MHz) titrations of butane-1-sulfonate (**G1**) into a methanol solution of **1**-BAr_F (0.25 mM) (a) and the corresponding binding isotherms (1:1 system) fitted by BINDFIT (b). A binding constant of $(8.7\pm0.5)\times10^2$ M^{-1} was obtained. The peaks of the guest are marked with asterisks.

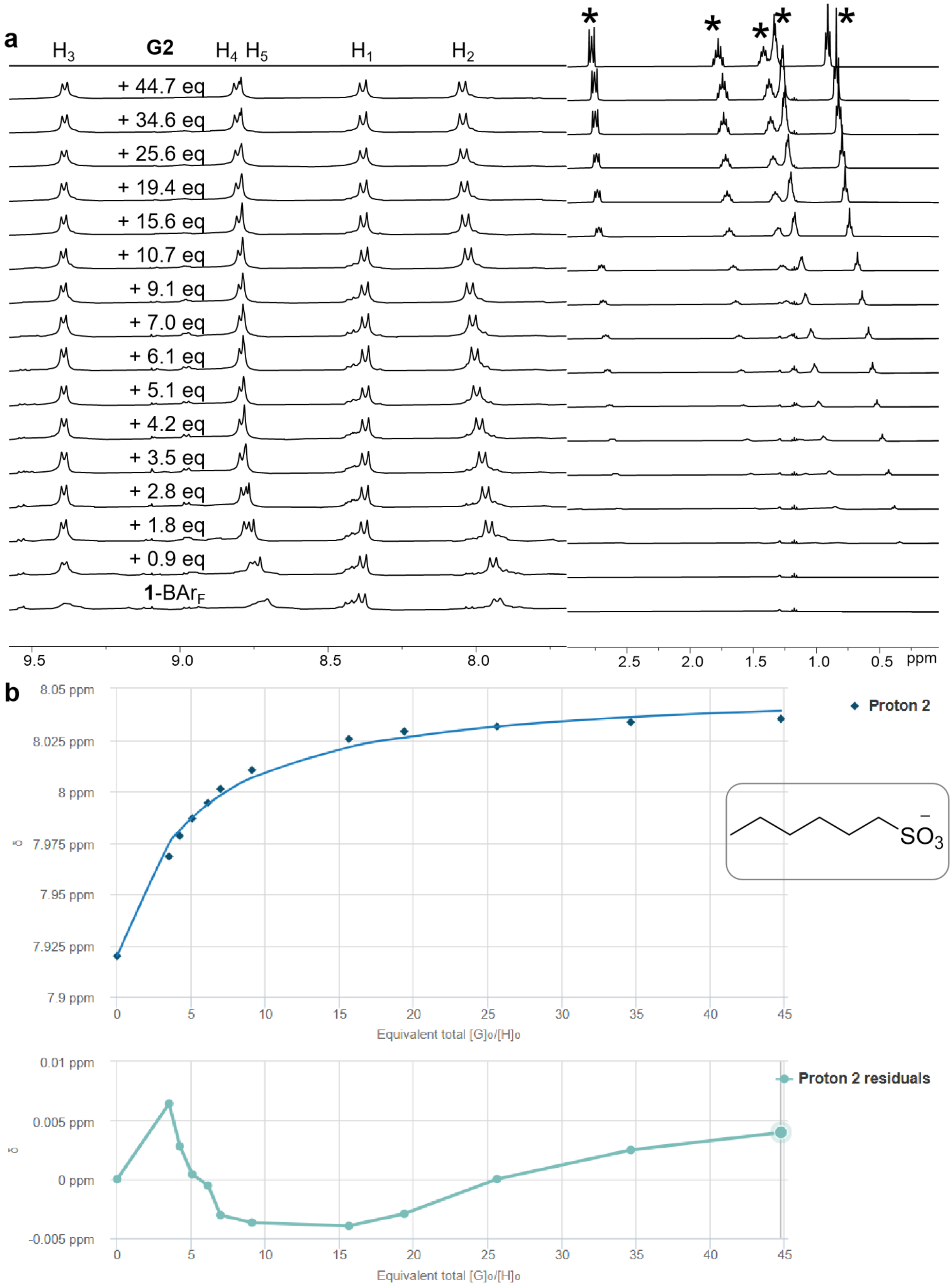

Figure S34. ^{1}H NMR (CD_3OD, 298 K, 400 MHz) titrations of hexane-1-sulfonate (**G2**) into a methanol solution of **1**-BAr$_F$ (0.25 mM) (a) and the corresponding binding isotherms (1:1 system) fitted by BINDFIT (b). A binding constant of (9.2±0.9)×10^2 M^{-1} was obtained. The peaks of the guest are marked with asterisks.

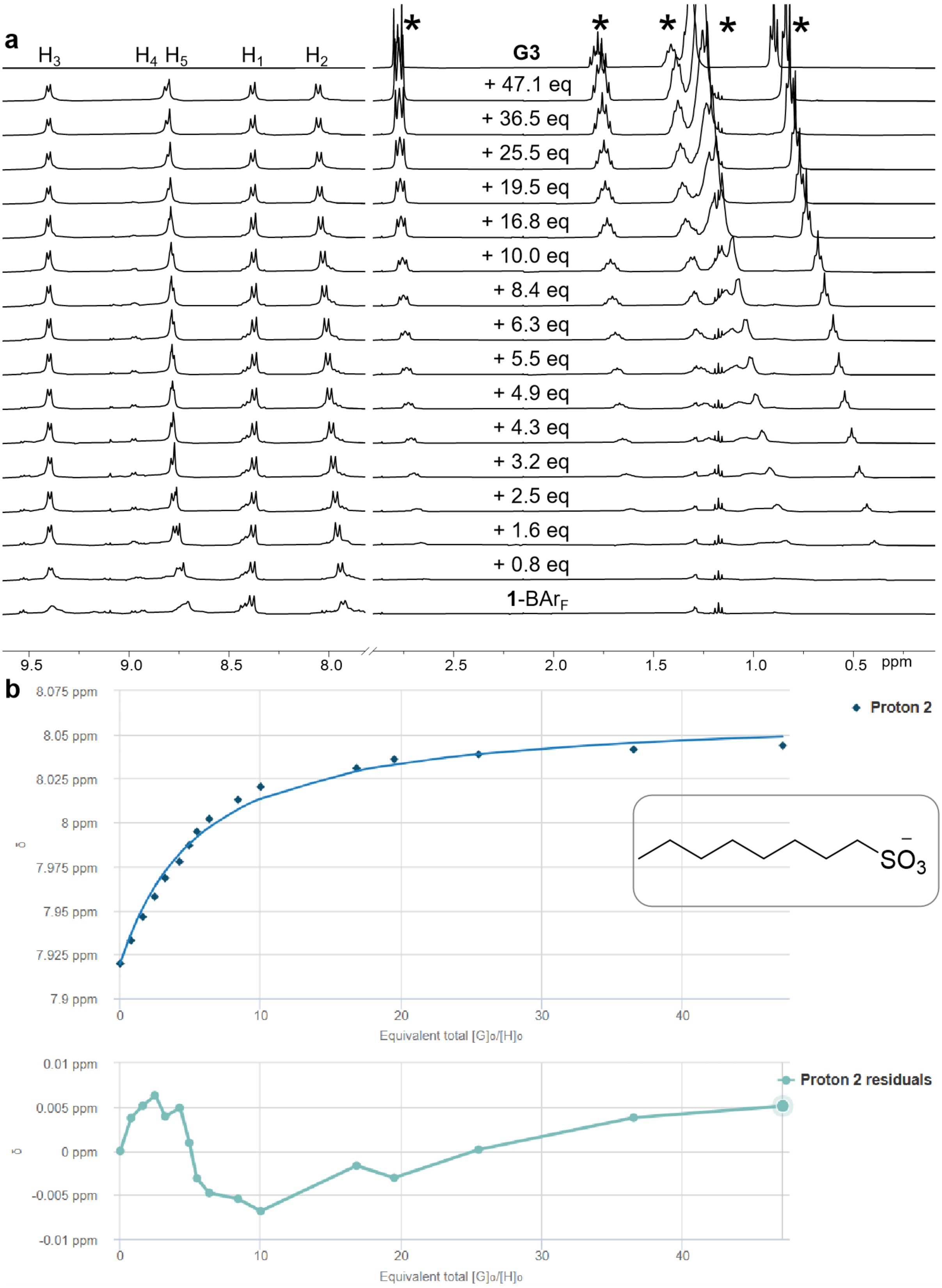

Figure S35. ^{1}H NMR (CD_3OD, 298 K, 400 MHz) titrations of octane-1-sulfonate (**G3**) into a methanol solution of **1**-BAr_F (0.25 mM) (a) and the corresponding binding isotherms (1:1 system) fitted by BINDFIT (b). A binding constant of $(8.2\pm0.8)\times10^2$ M^{-1} was obtained. The peaks of the guest are marked with asterisks.

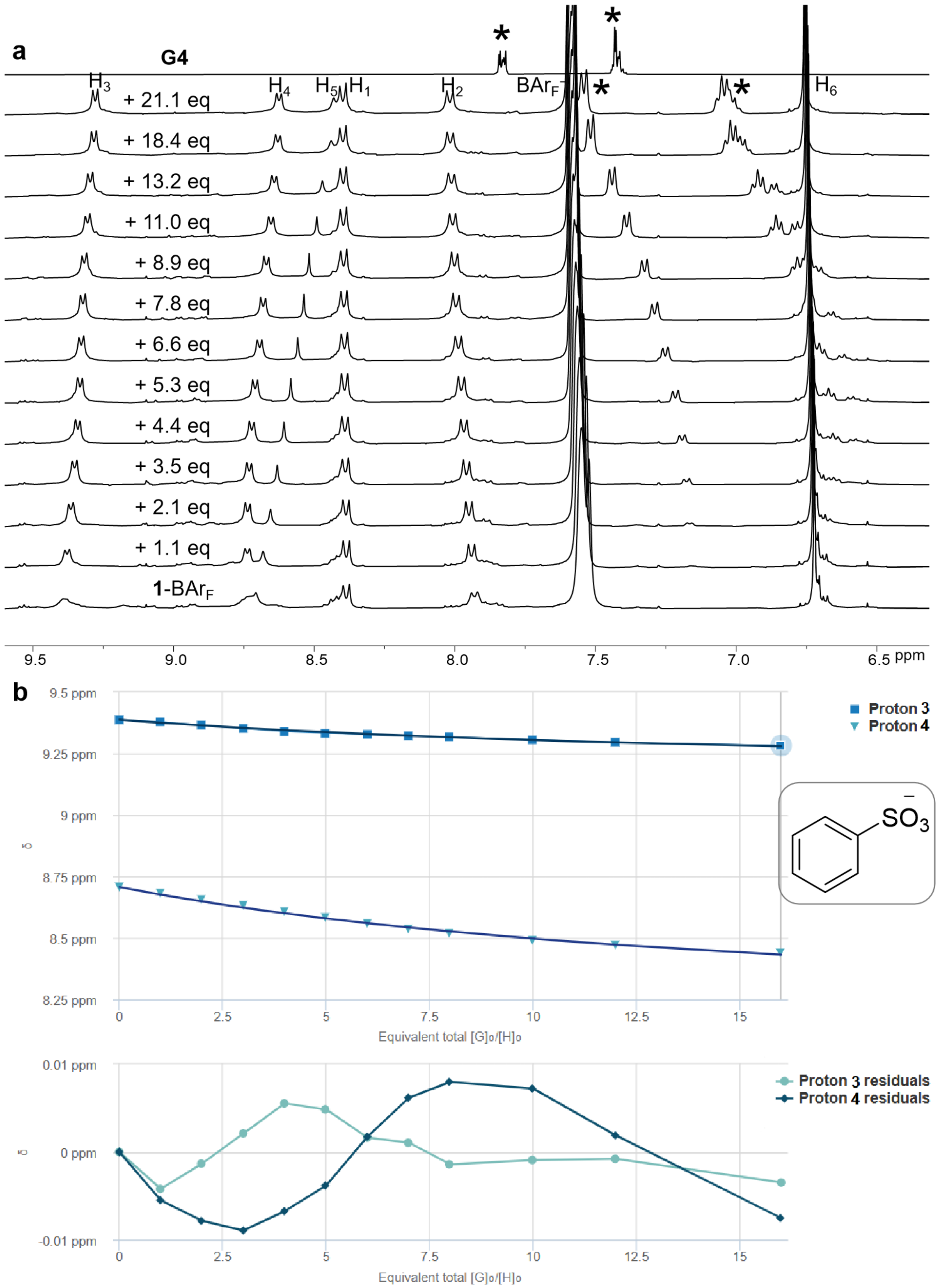

Figure S36. ^{1}H NMR (CD_3OD, 298 K, 400 MHz) titrations of benzenesulfonate (**G4**) into a methanol solution of **1**-BAr_F (0.25 mM) (a) and the corresponding binding isotherms (1:1 system) fitted by BINDFIT (b). A binding constant of $(2.4\pm0.2)\times10^2$ M^{-1} was obtained. The peaks of the guest are marked with asterisks.

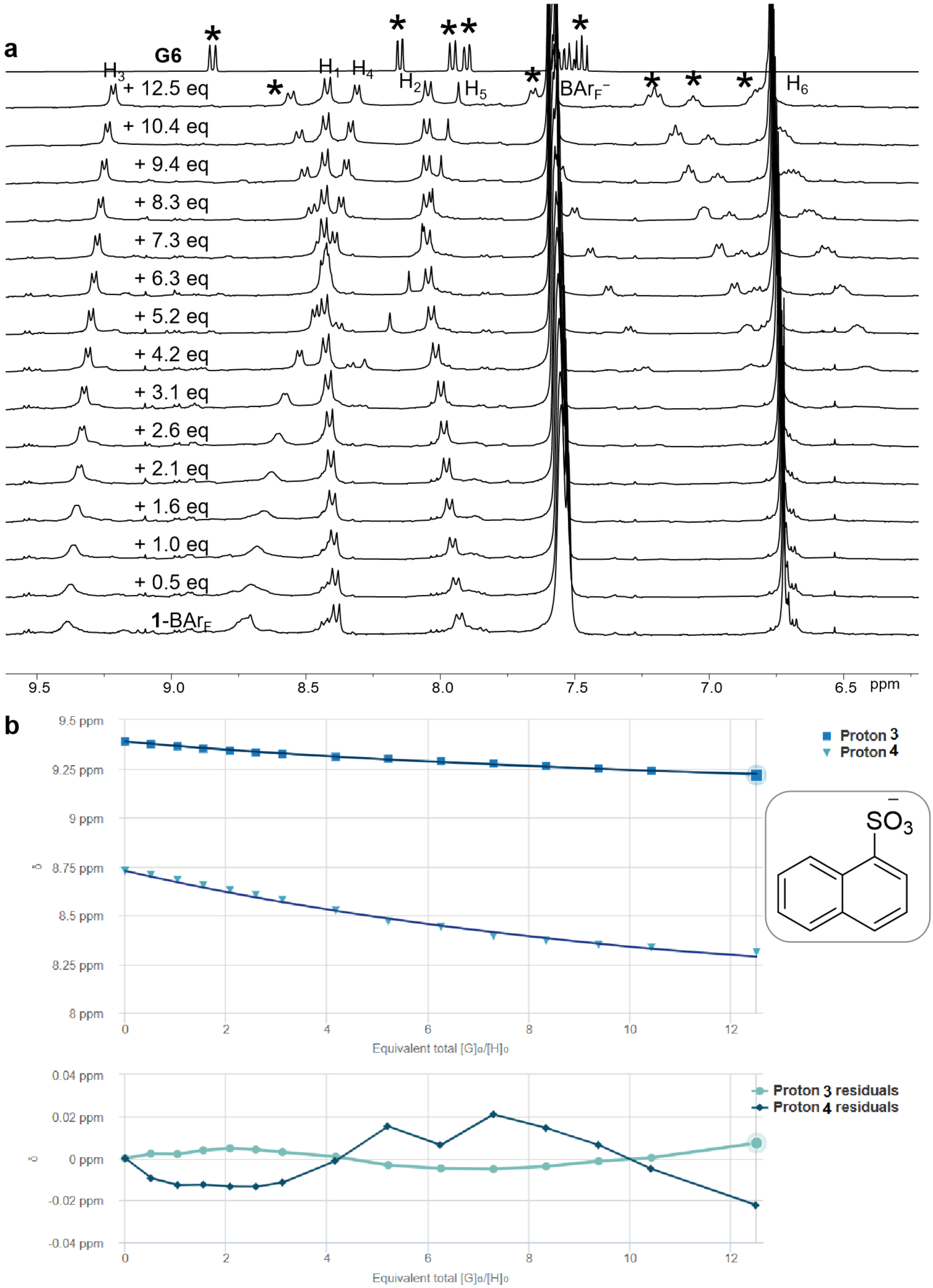

Figure S37. ^{1}H NMR (CD_3OD, 298 K, 400 MHz) titrations of naphthalene-1-sulfonate (**G6**) into a methanol solution of **1**-BAr_F (0.25 mM) (a) and the corresponding binding isotherms (1:1 system) fitted by BINDFIT (b). A binding constant of $(2.3\pm0.1)\times10^2$ M^{-1} was obtained. The peaks of the guest are marked with asterisks.

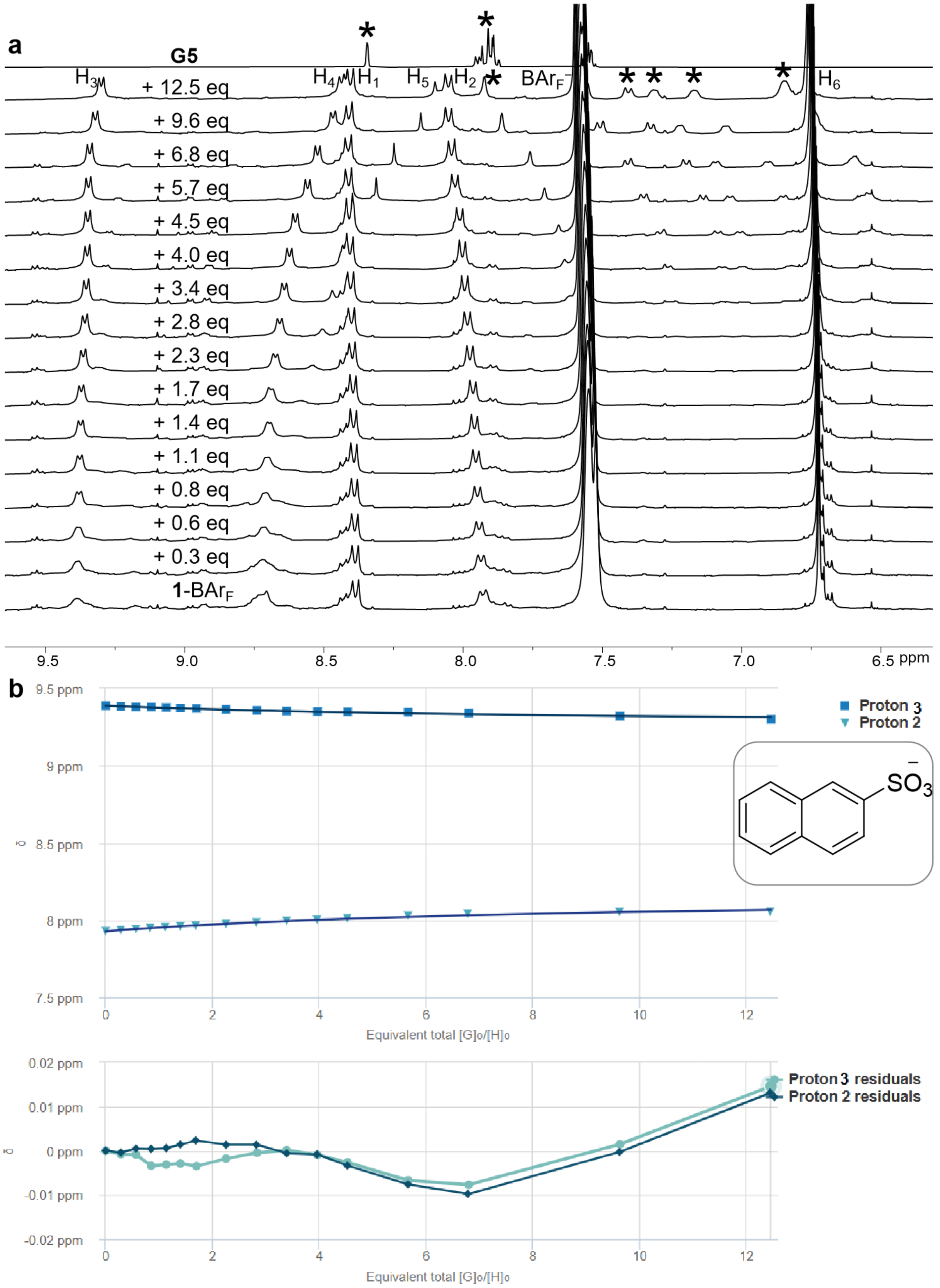

Figure S38. ^{1}H NMR (CD_3OD, 298 K, 400 MHz) titrations of naphthalene-2-sulfonate (**G5**) into a methanol solution of **1**-BAr$_F$ (0.25 mM) (a) and the corresponding binding isotherms (1:1 system) fitted by BINDFIT (b). A binding constant of $(4.7\pm0.3)\times10^{2}$ M^{-1} was obtained. The peaks of the guest are marked with asterisks.

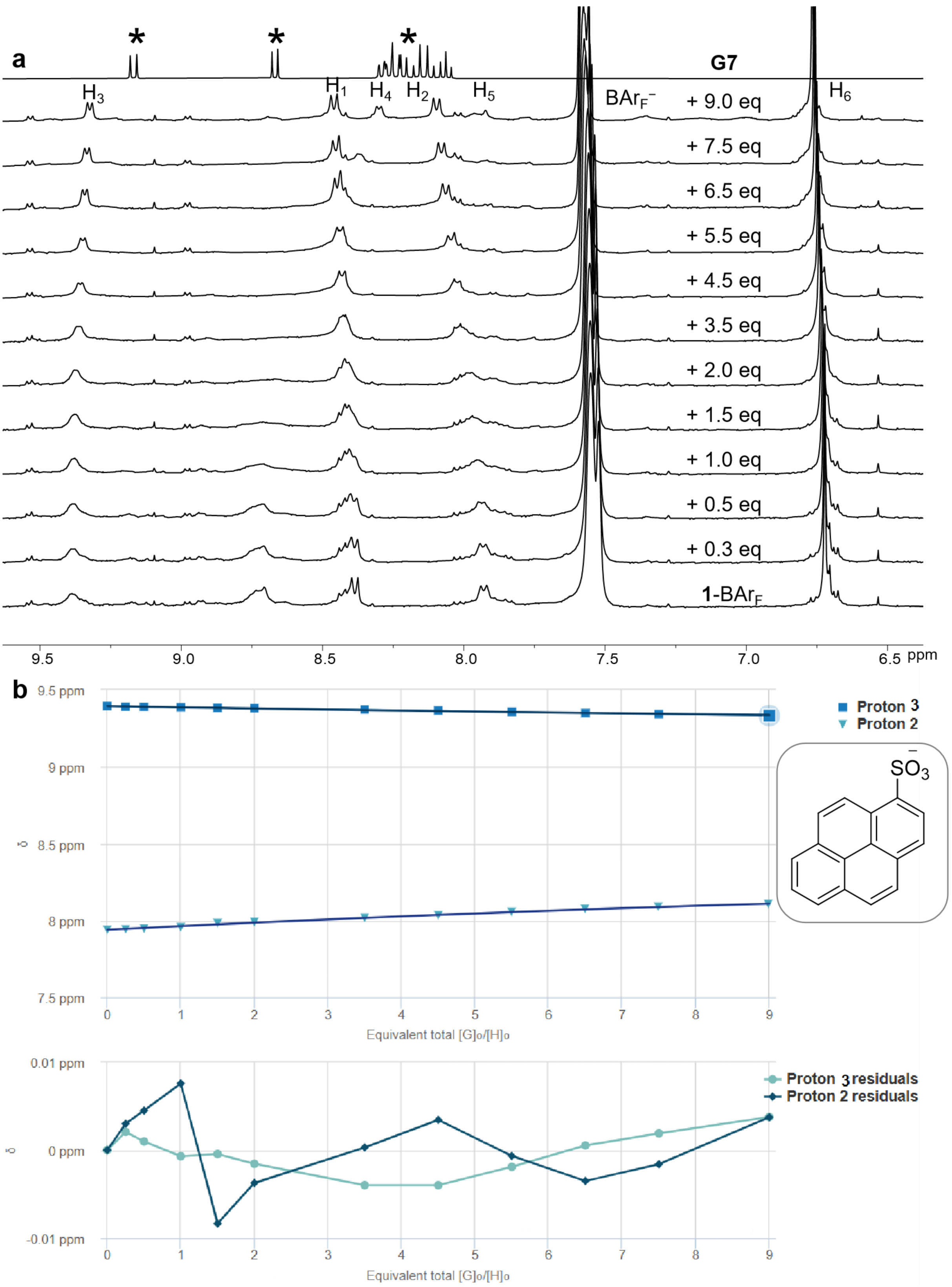

Figure S39. ^{1}H NMR (CD_3OD, 298 K, 400 MHz) titrations of pyrene-1-sulfonate (**G7**) into a methanol solution of **1**-BAr_F (0.25 mM) (a) and the corresponding binding isotherms (1:1 system) fitted by BINDFIT (b). A binding constant of $(1.1\pm0.1)\times10^2$ M^{-1} was obtained. The peaks of the guest are marked with asterisks.

3.2 ^{1}H NMR titrations of disulfonates

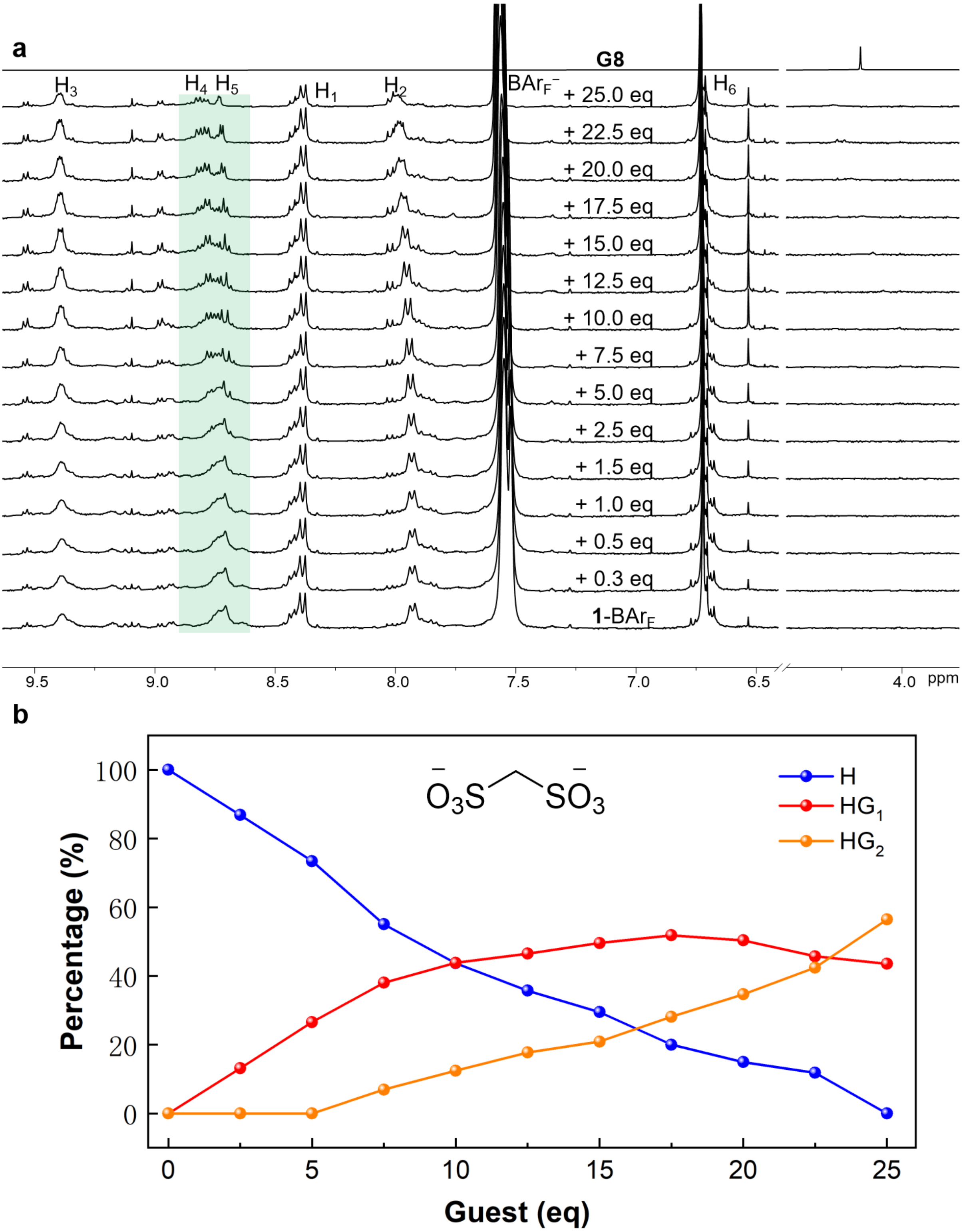

Figure S40. ^{1}H NMR (CD_3OD, 298 K, 400 MHz) titrations of methanedisulfonate (**G8**) into a methanol solution of **1**-BAr_F (0.25 mM) (a) and species distribution map (b). The binding constants were calculated based on H_4.

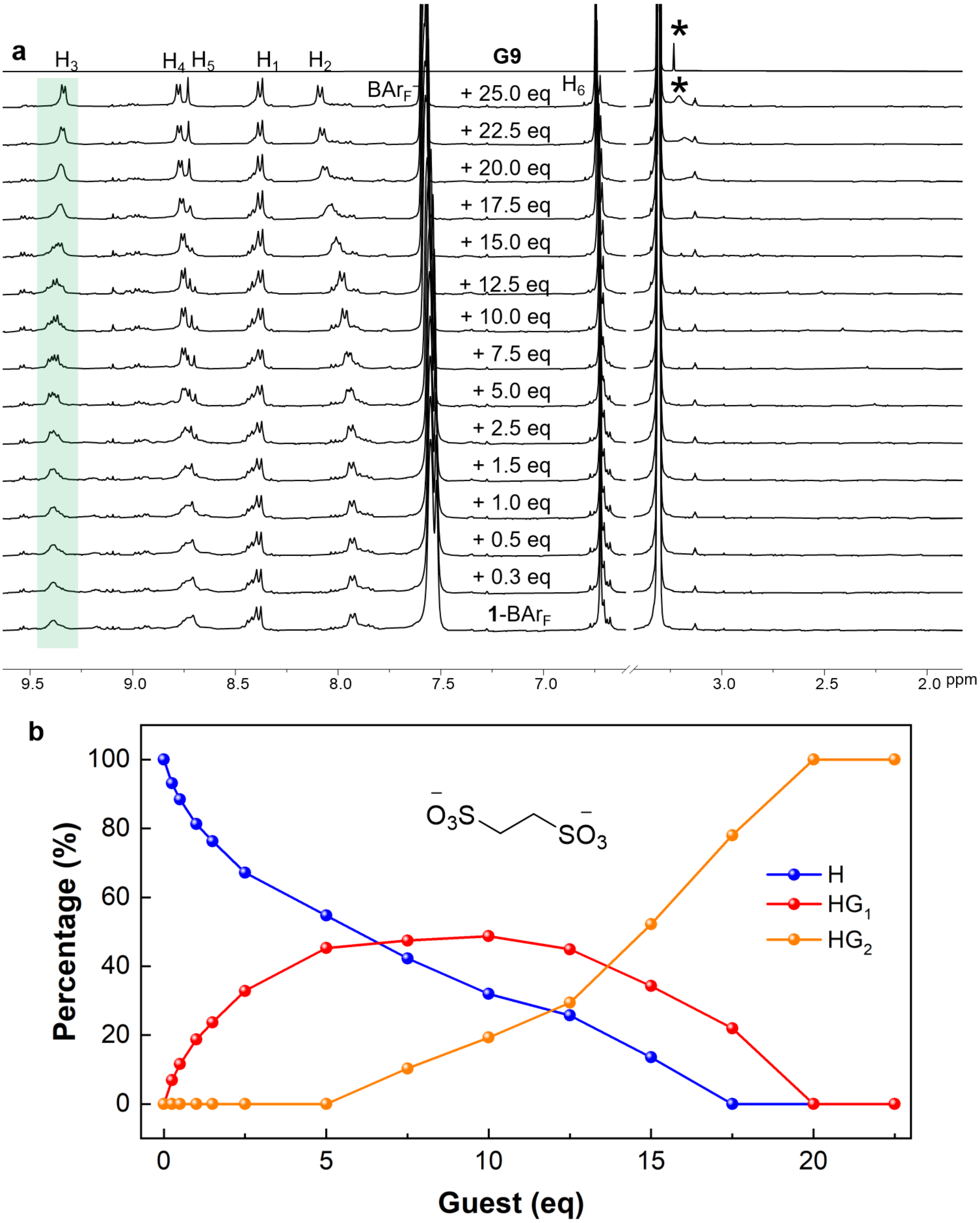

Figure S41. ^{1}H NMR (CD_3OD, 298 K, 400 MHz) titrations of ethane-1,2-disulfonate (**G9**) into a methanol solution of **1**-BAr$_F$ (0.25 mM) (a) and species distribution map (b). The binding constant is calculated based on H_3.

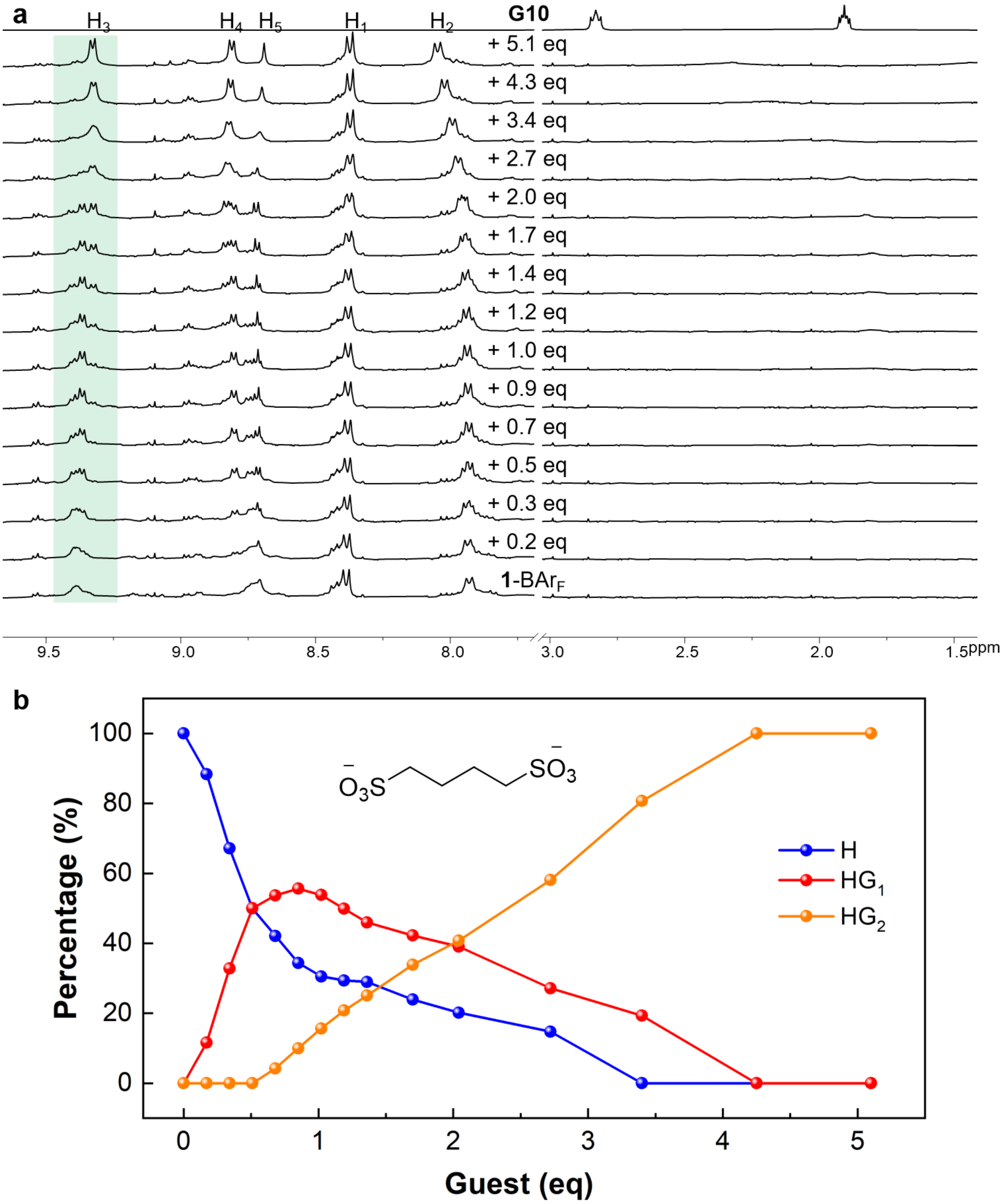

Figure S42. ^{1}H NMR (CD_3OD, 298 K, 400 MHz) titrations of butane-1,4-disulfonate (**G10**) into a methanol solution of **1**-BAr$_F$ (0.25 mM) (a) and species distribution map (b). The binding constant is calculated based on H_3.

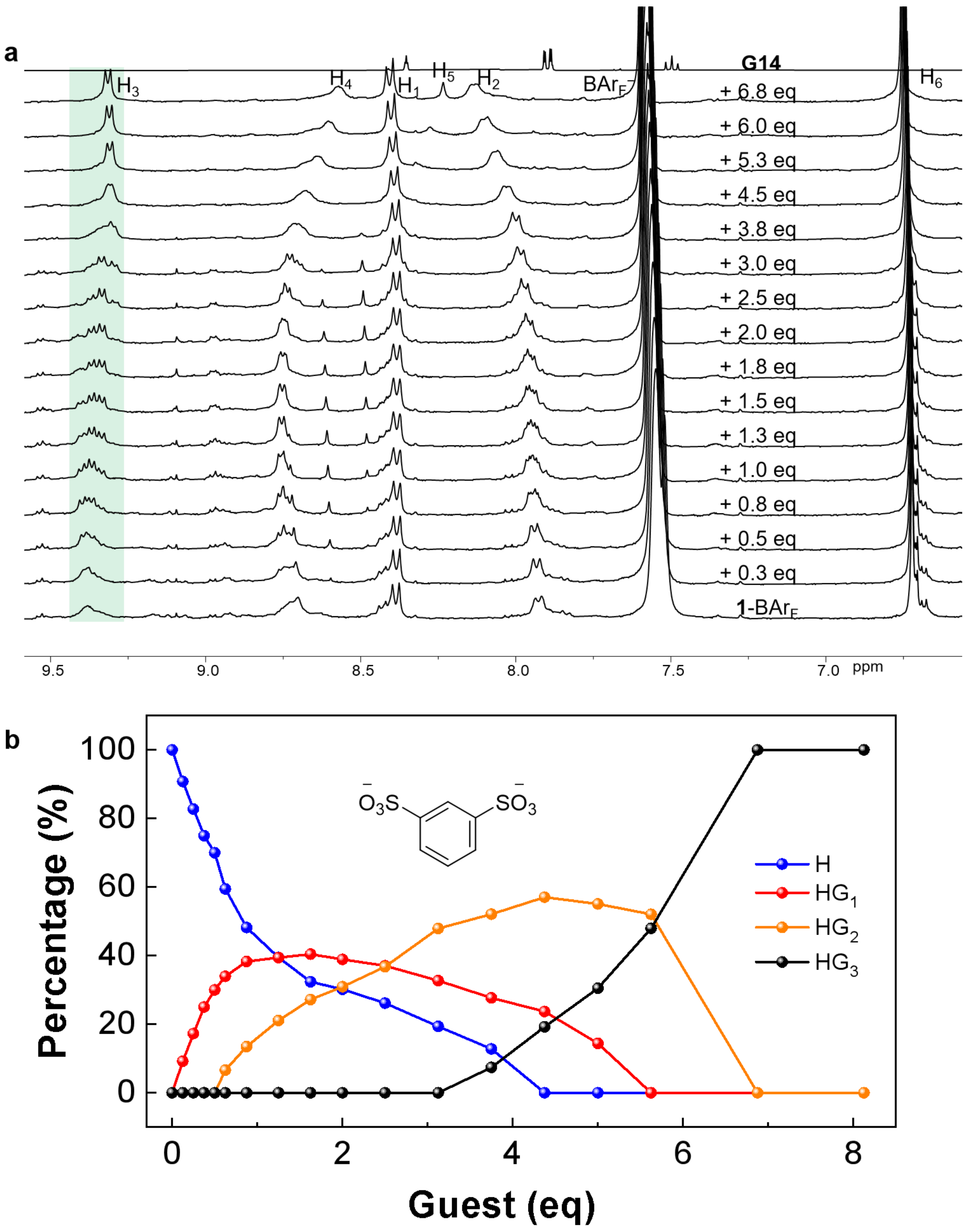

Figure S43. ^{1}H NMR (CD_3OD, 298 K, 400 MHz) titrations of benzene-1,3-disulfonate (**G14**) into a methanol solution of **1**-BAr_F (0.25 mM) (a) and species distribution map (b). The binding constant is calculated based on H_3.

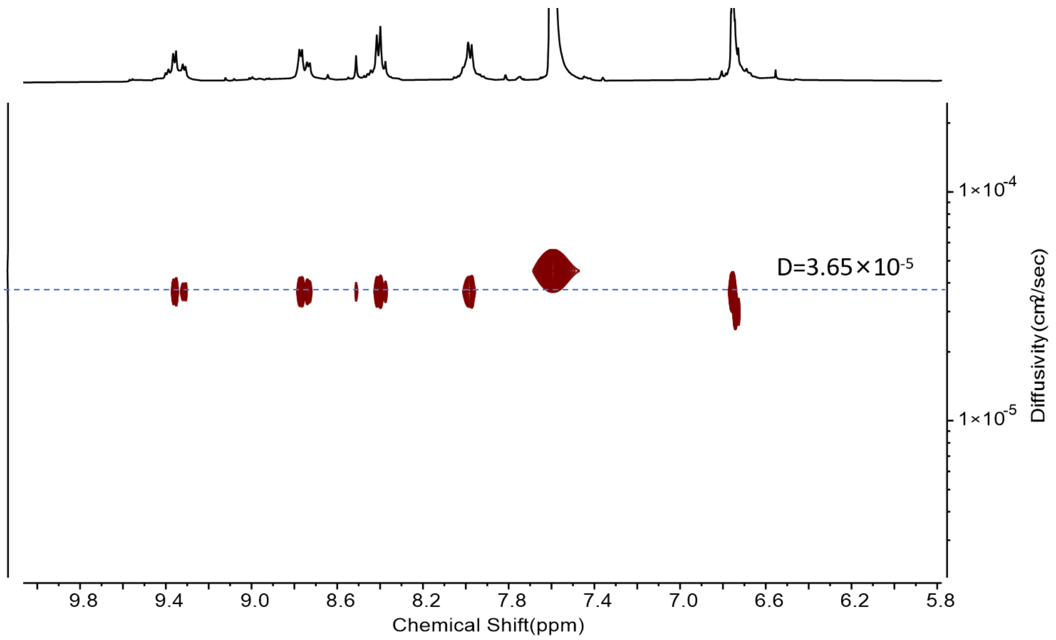

Figure S44. DOSY spectrum (CD_3OD, 298 K, 500 MHz) of **1**-BAr_F in the presence of 3.0 equiv. **G14**. Under the measurement conditions, the cage exists as 1:1, 1:2 and 1:3 host-guest complexes with **G14**. These three species show similar molecular weight and size and thus have similar diffusion coefficients.

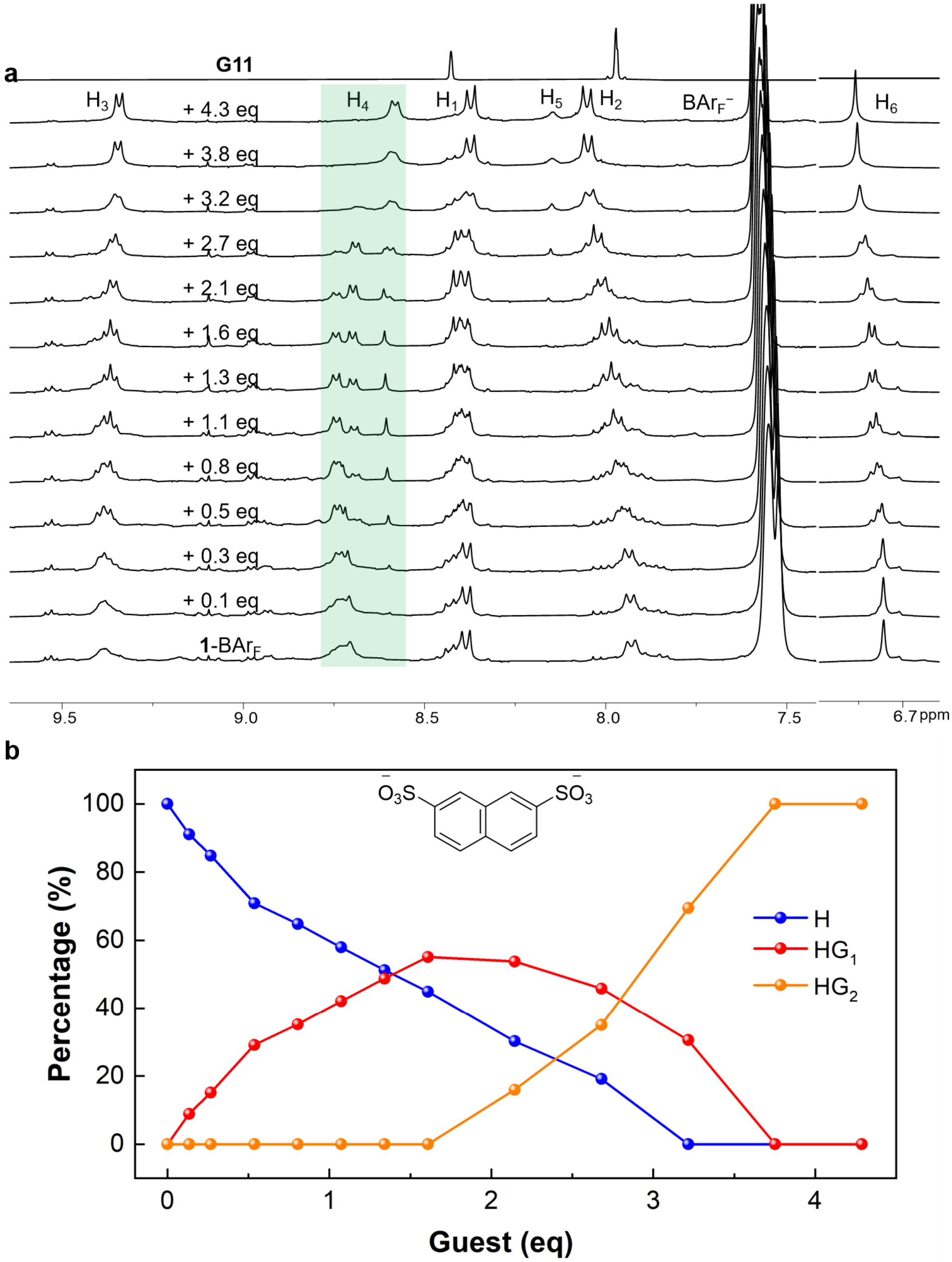

Figure S45. ^{1}H NMR (CD_3OD, 298 K, 400 MHz) titrations of naphthalene-2,7-disulfonate (**G11**) into a methanol solution of **1**-BAr_F (0.25 mM) (a) and species distribution map (b). The binding constant is calculated based on H_4.

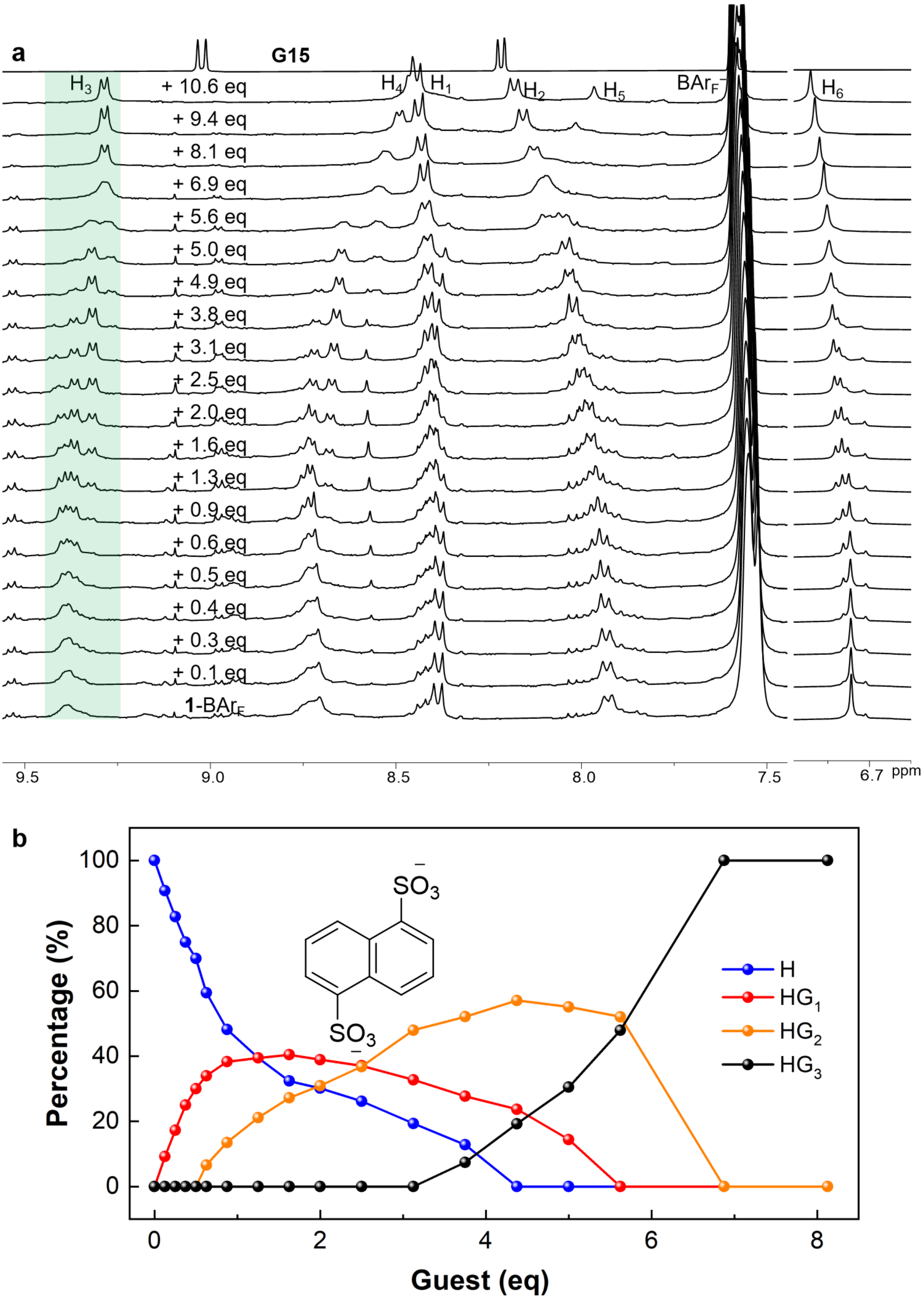

Figure S46. ^{1}H NMR (CD_3OD, 298 K, 400 MHz) titrations of naphthalene-1,5-disulfonate (**G15**) into a methanol solution of **1**-BAr_F (0.25 mM) (a) and species distribution map (b). The binding constant is calculated based on H_3.

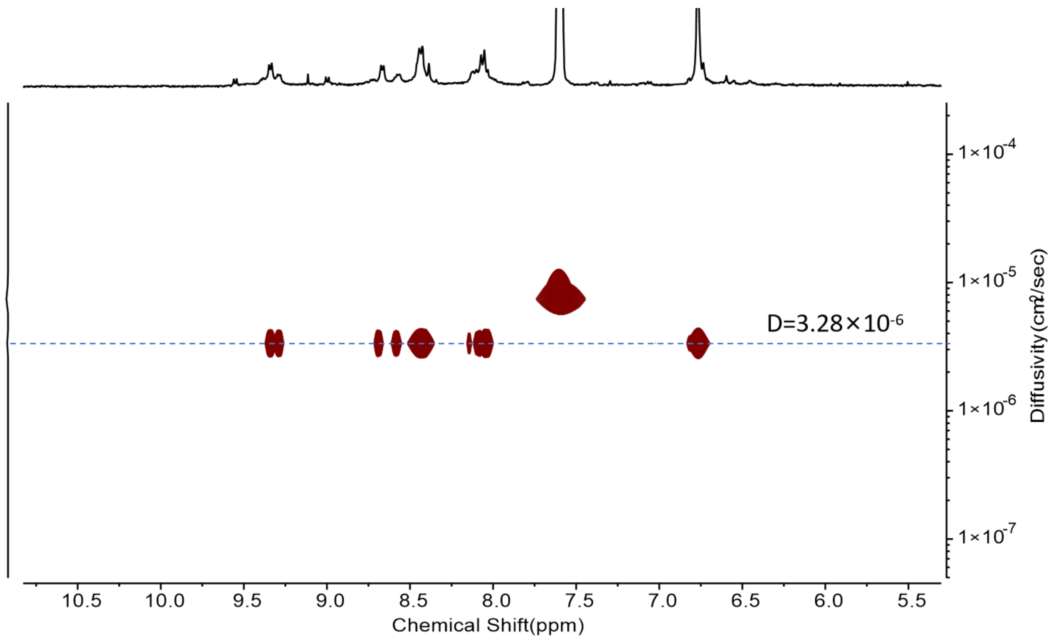

Figure S47. DOSY spectrum (CD_3OD, 298 K, 500 MHz) of **1**-BAr$_F$ in the presence of 5.0 equiv. **G15**. Under the measurement conditions, the cage exists as 1:1, 1:2 and 1:3 host-guest complexes with **G15**. These three species show similar molecular weight and size and thus have similar diffusion coefficients.

3.3 ^{1}H NMR titrations of trisulfonates

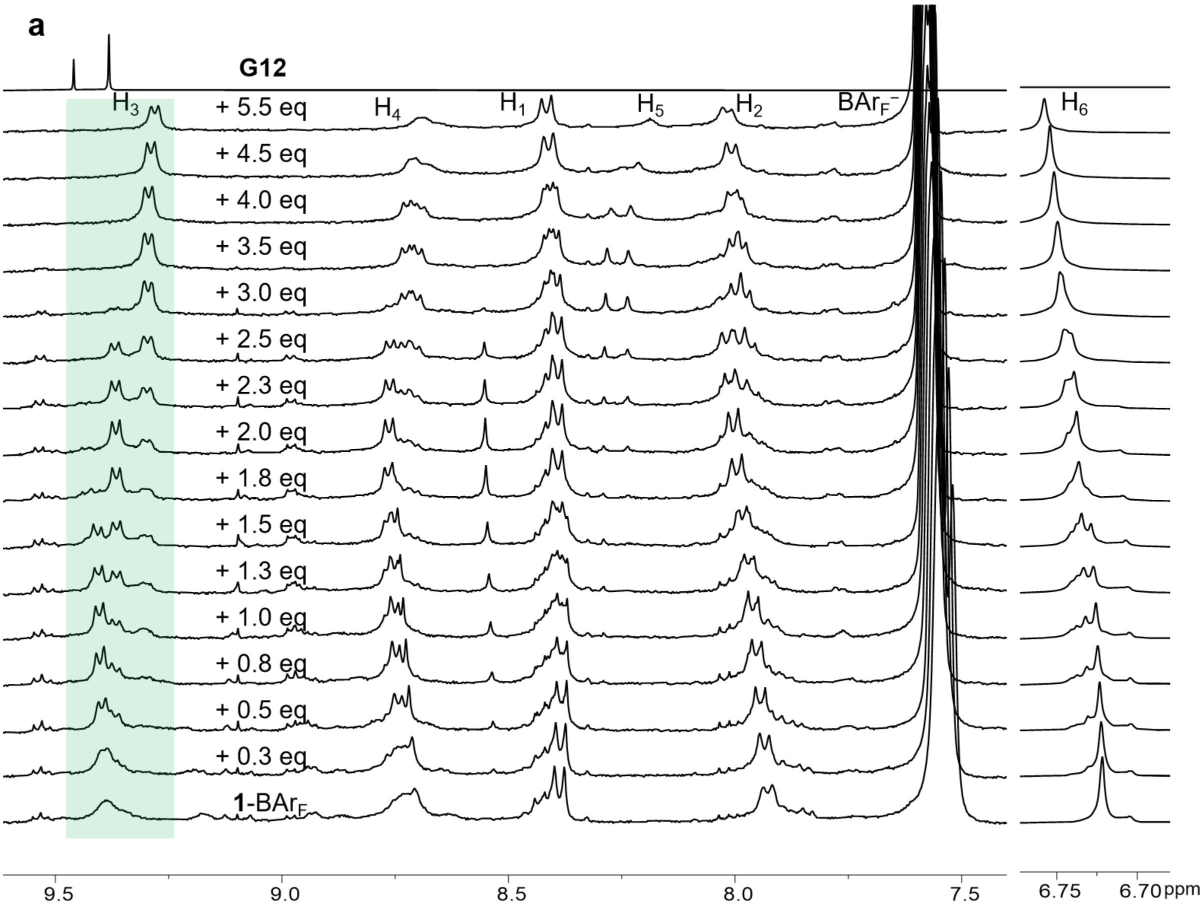

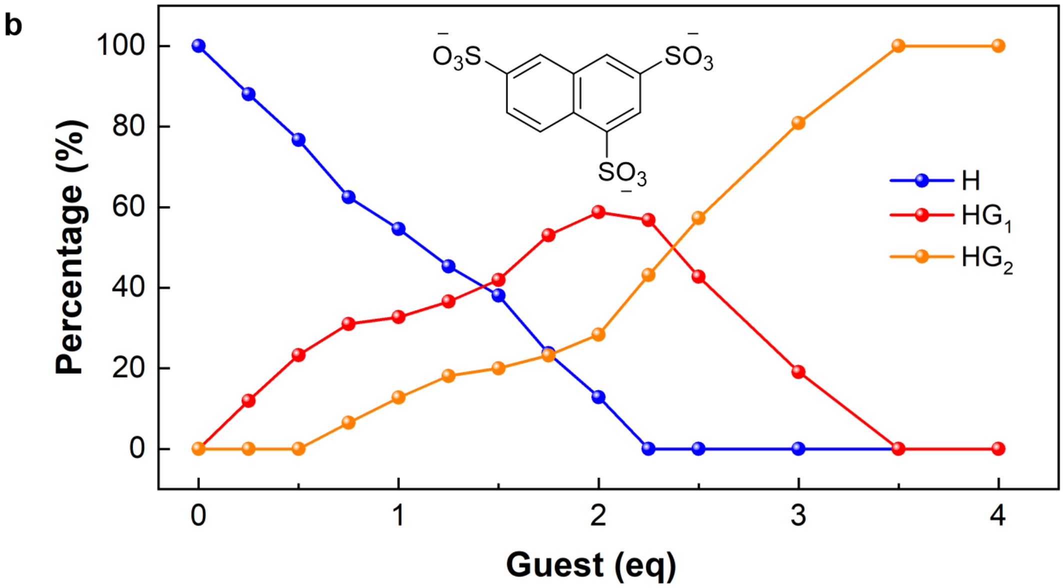

Figure S48. ^{1}H NMR (CD_3OD, 298 K, 400 MHz) titrations of naphthalene-1,3,6-trisulfonate (**G12**) into a methanol solution of **1**-BAr$_F$ (0.25 mM) (a) and species distribution map (b). The binding constant is calculated based on H_3.

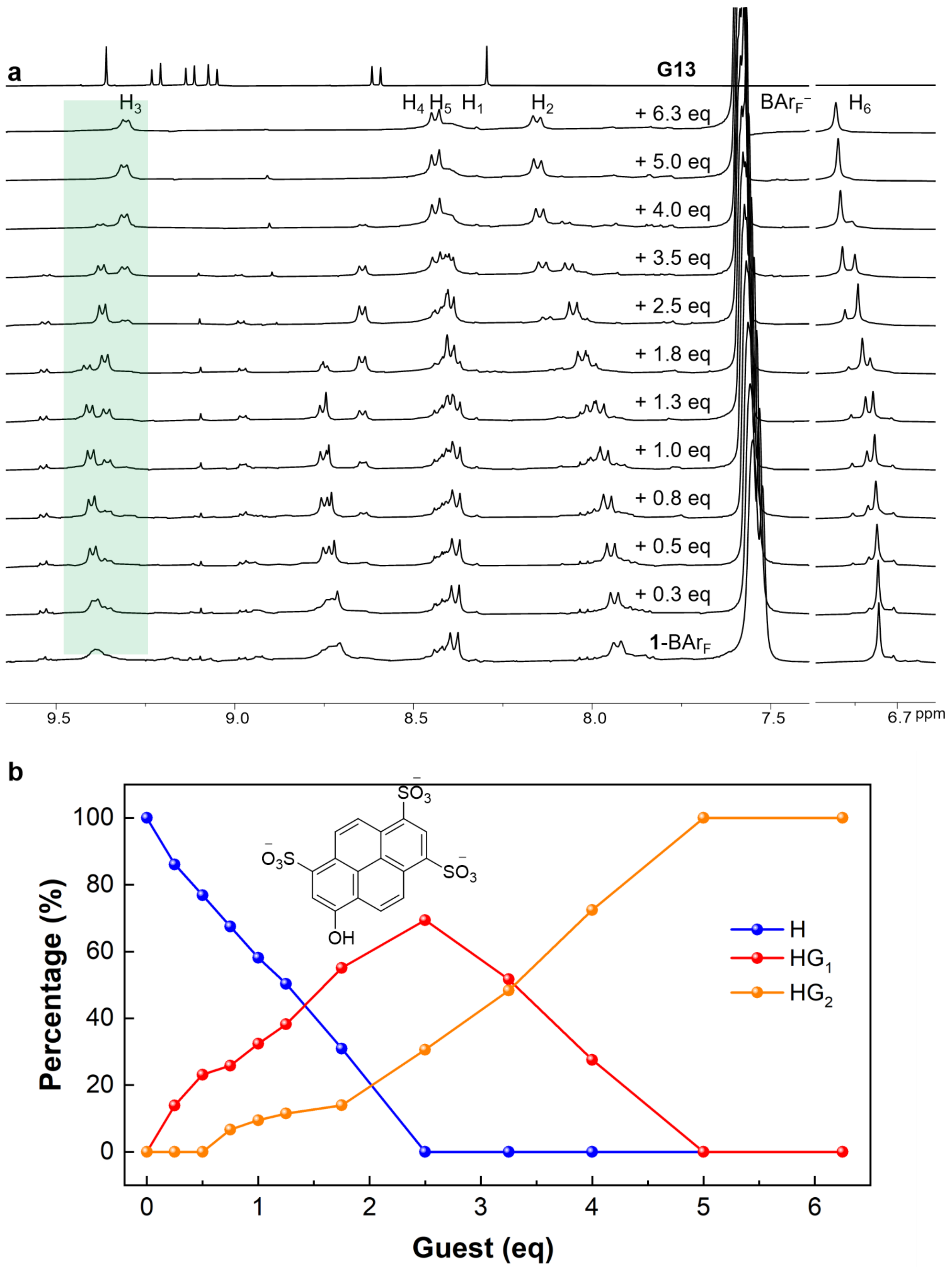

Figure S49. ^{1}H NMR (CD_3OD, 298 K, 400 MHz) titrations of 8-hydroxypyrene-1,3,6-trisulfonate (**G13**) into a methanol solution of **1**-BAr$_F$ (0.25 mM) (a) and species distribution map (b). The binding constant is calculated based on H_3.

3.4 ¹H NMR titrations of tetrasulfonates

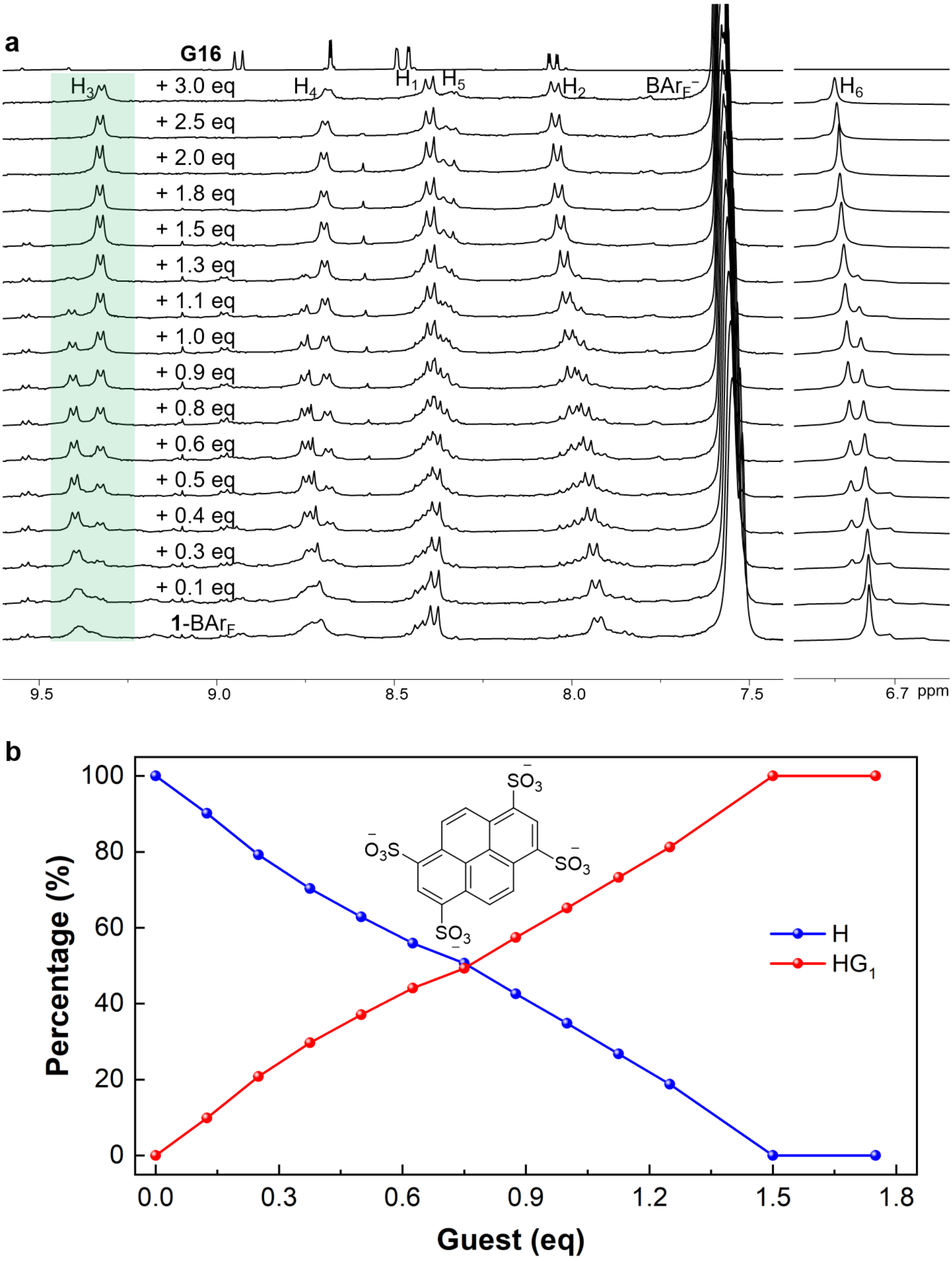

Figure S50. ¹H NMR (CD_3OD, 298 K, 400 MHz) titrations of pyrene-1,3,6,8-tetrasulfonate (**G16**) into a methanol solution of **1**-BAr_F (0.25 mM) (a) and species distribution map (b). The binding constant is calculated based on H_3.

3.5 NMR titrations of perfluorosulfonates

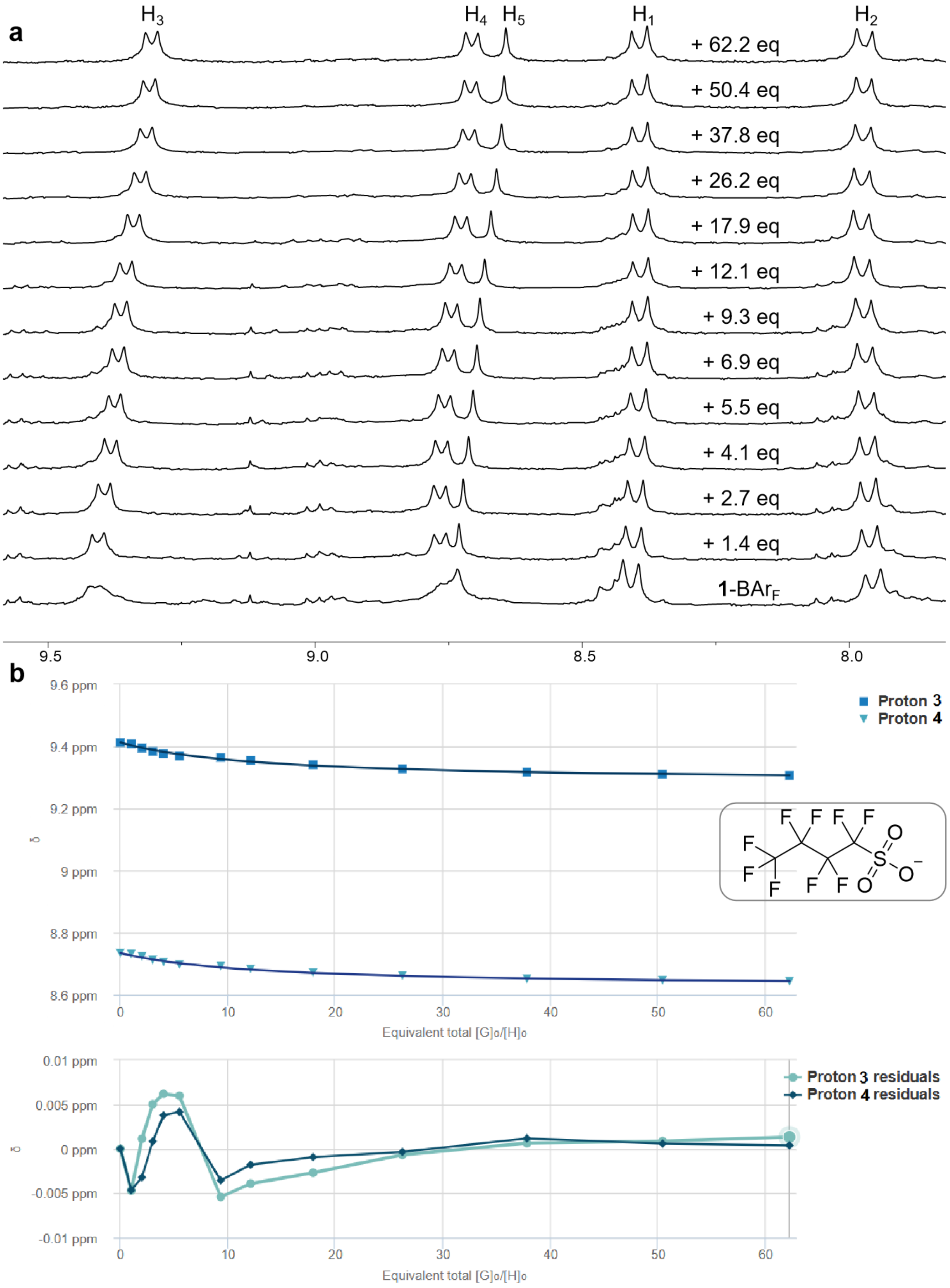

Figure S51. ^{1}H NMR (CD_3OD, 298 K, 400 MHz) titrations of PFBS into a methanol solution of **1**-BAr$_F$ (0.25 mM) (a) and the corresponding binding isotherms (1:1 system) fitted by BINDFIT (b). A binding constant of $(3.5\pm0.2)\times10^2$ M^{-1} was obtained.

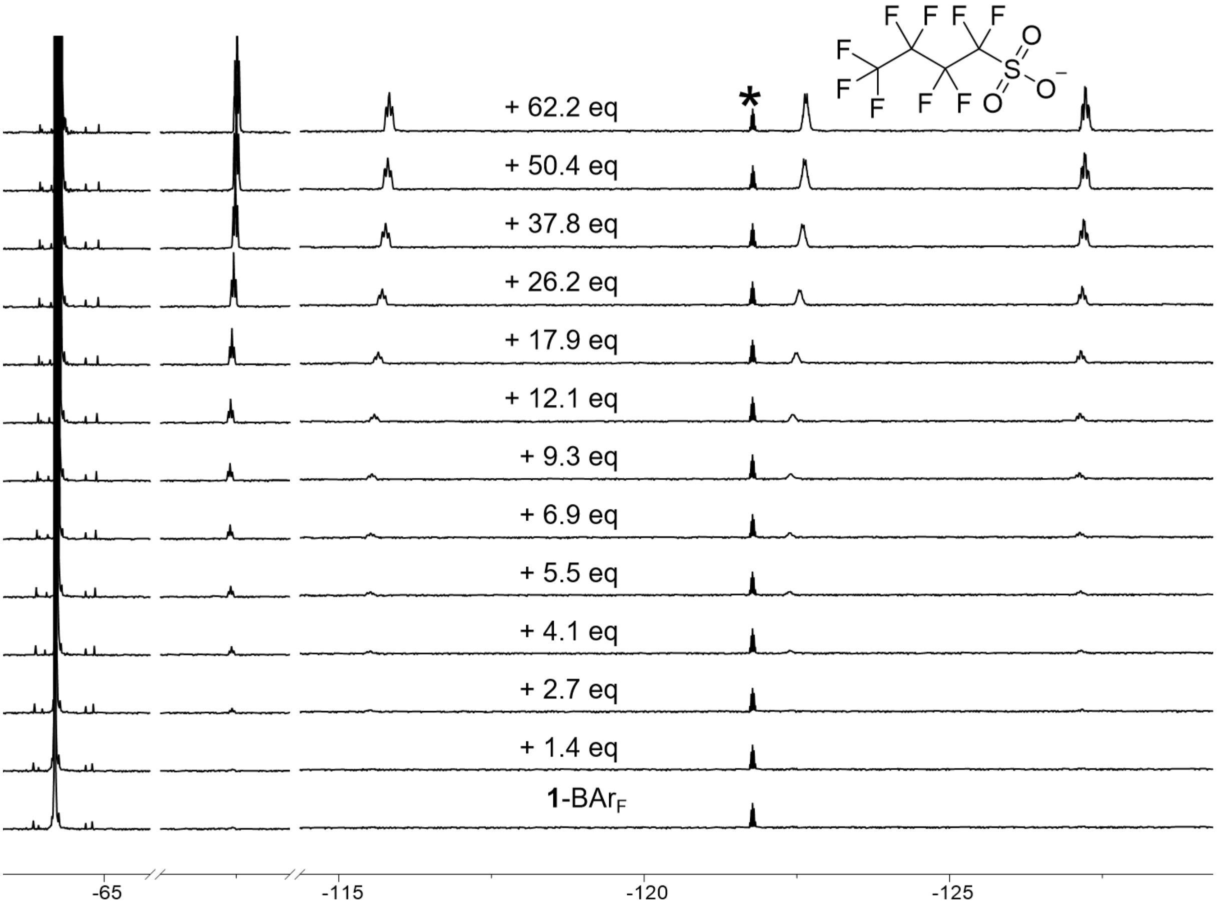

Figure S52. ^{19}F NMR (CD_3OD, 298 K, 376 MHz) titrations of PFBS into a methanol solution of **1**-BAr_F (0.25 mM). The peak of 1,4-difluorobenzene (internal standard) is marked with an asterisk.

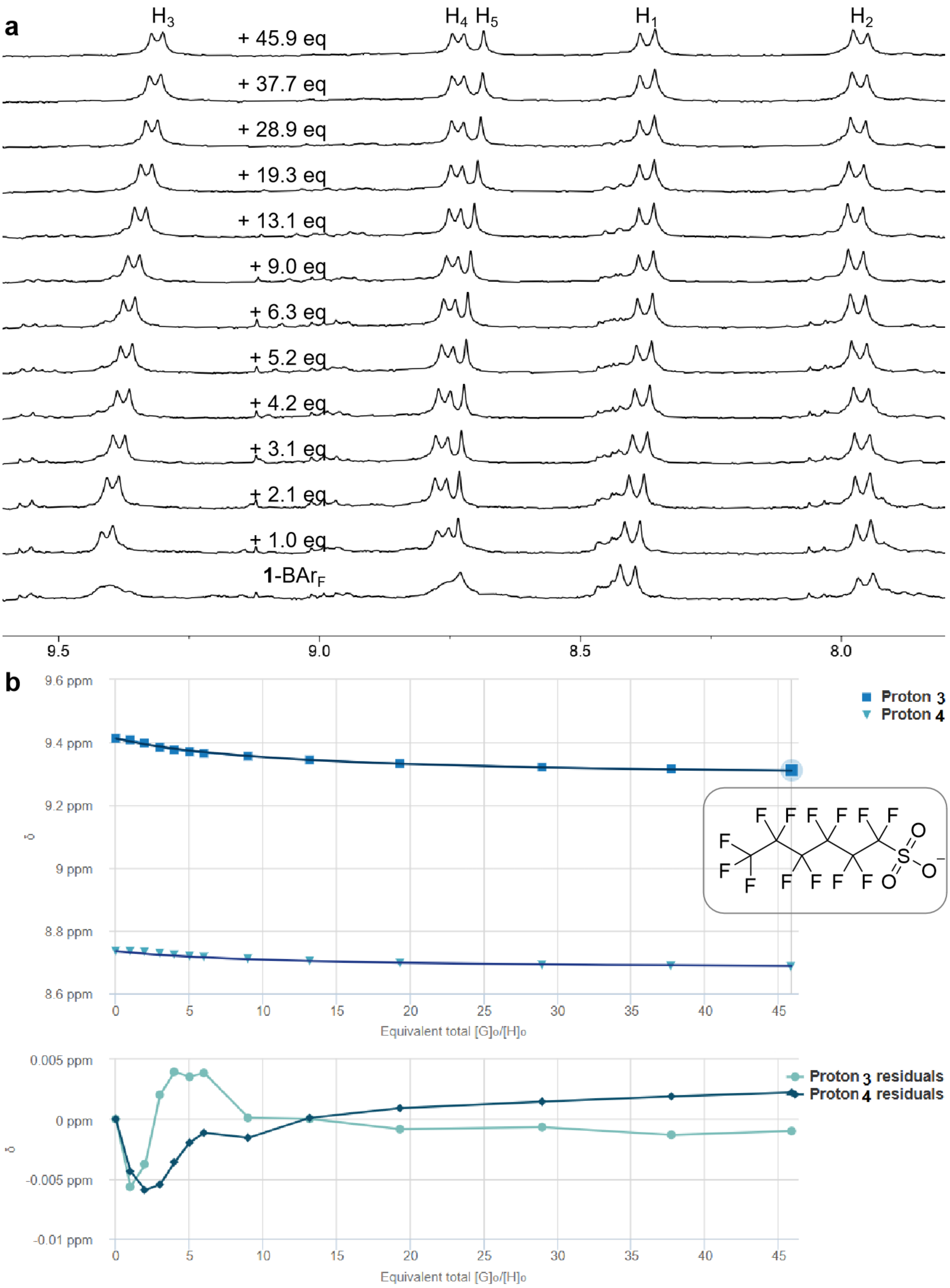

Figure S53. ^{1}H NMR (CD_3OD, 298 K, 400 MHz) titrations of PFHxS into a methanol solution of **1**-BAr_F (0.25 mM) (a) and the corresponding binding isotherms (1:1 system) fitted by BINDFIT (b). A binding constant of $(3.8\pm0.3)\times10^{2}$ M^{-1} was obtained.

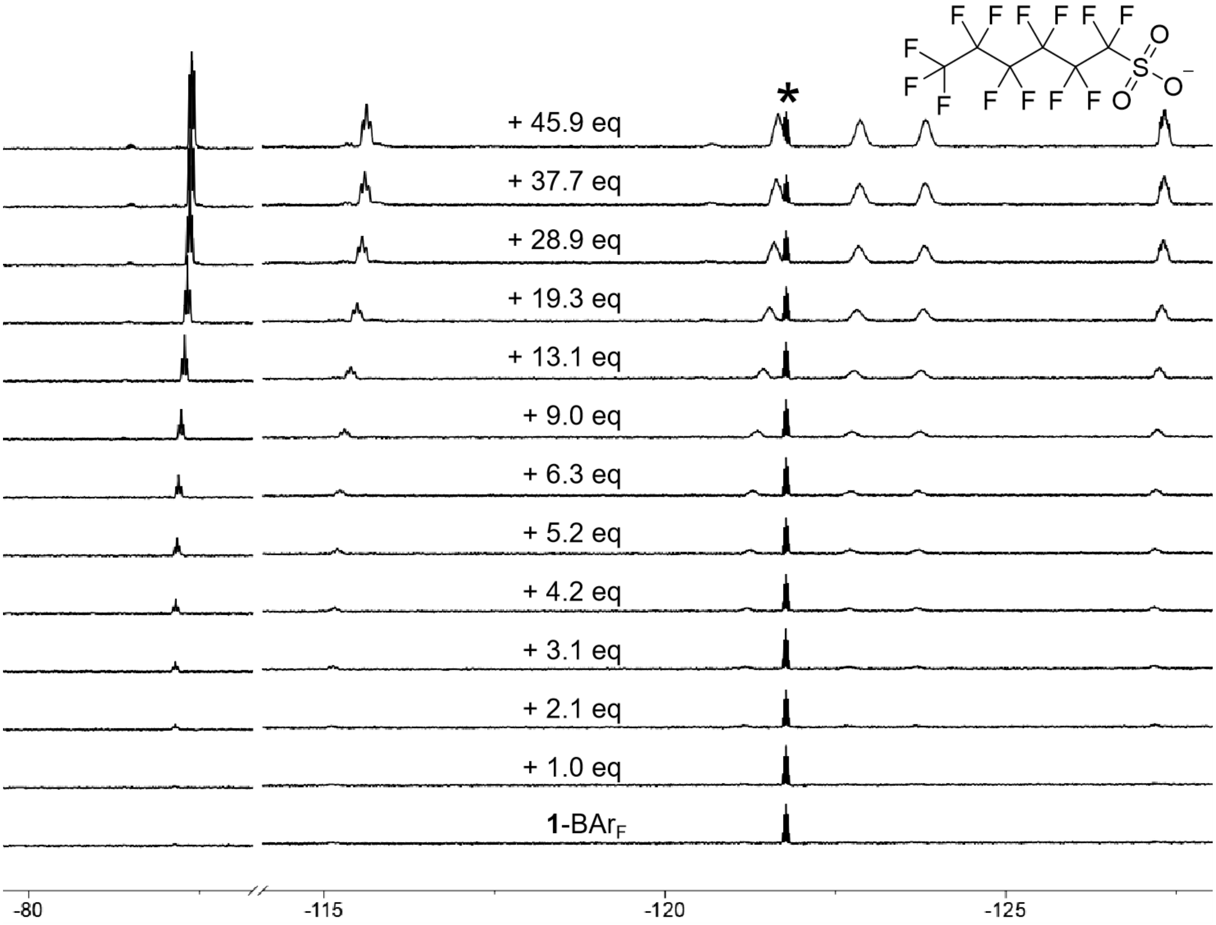

Figure S54. ^{19}F NMR (CD_3OD, 298 K, 376 MHz) titrations of PFHxS into a methanol solution of **1**-BAr_F (0.25 mM). The peak of 1,4-difluorobenzene (internal standard) is marked with an asterisk.

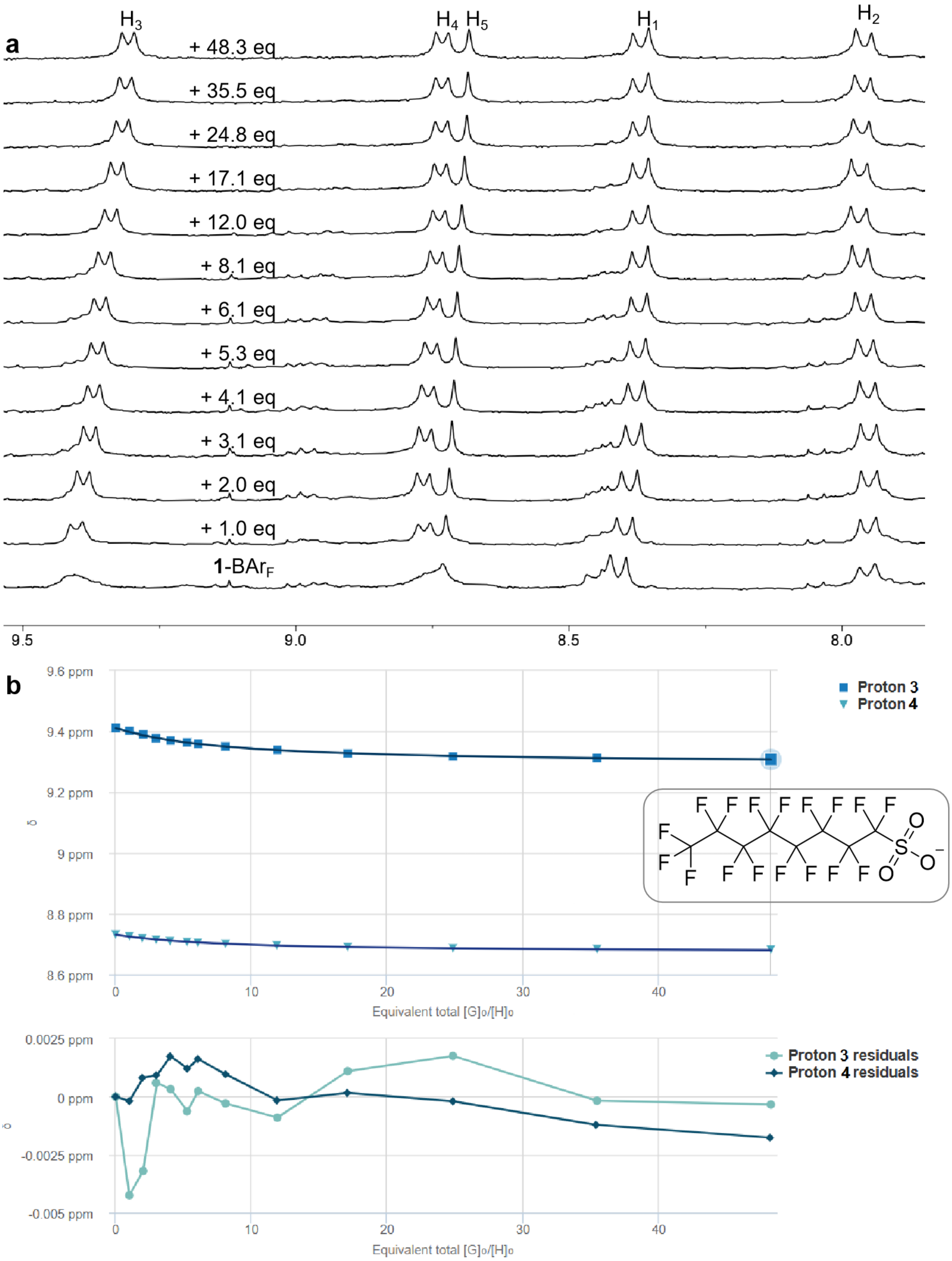

Figure S55. ^{1}H NMR (CD_3OD, 298 K, 400 MHz) titrations of PFOS into a methanol solution of **1**-BAr_F (0.25 mM) (a) and the corresponding binding isotherms (1:1 system) fitted by BINDFIT (b). A binding constant of $(5.7\pm0.2)\times10^{2}$ M^{-1} was obtained.

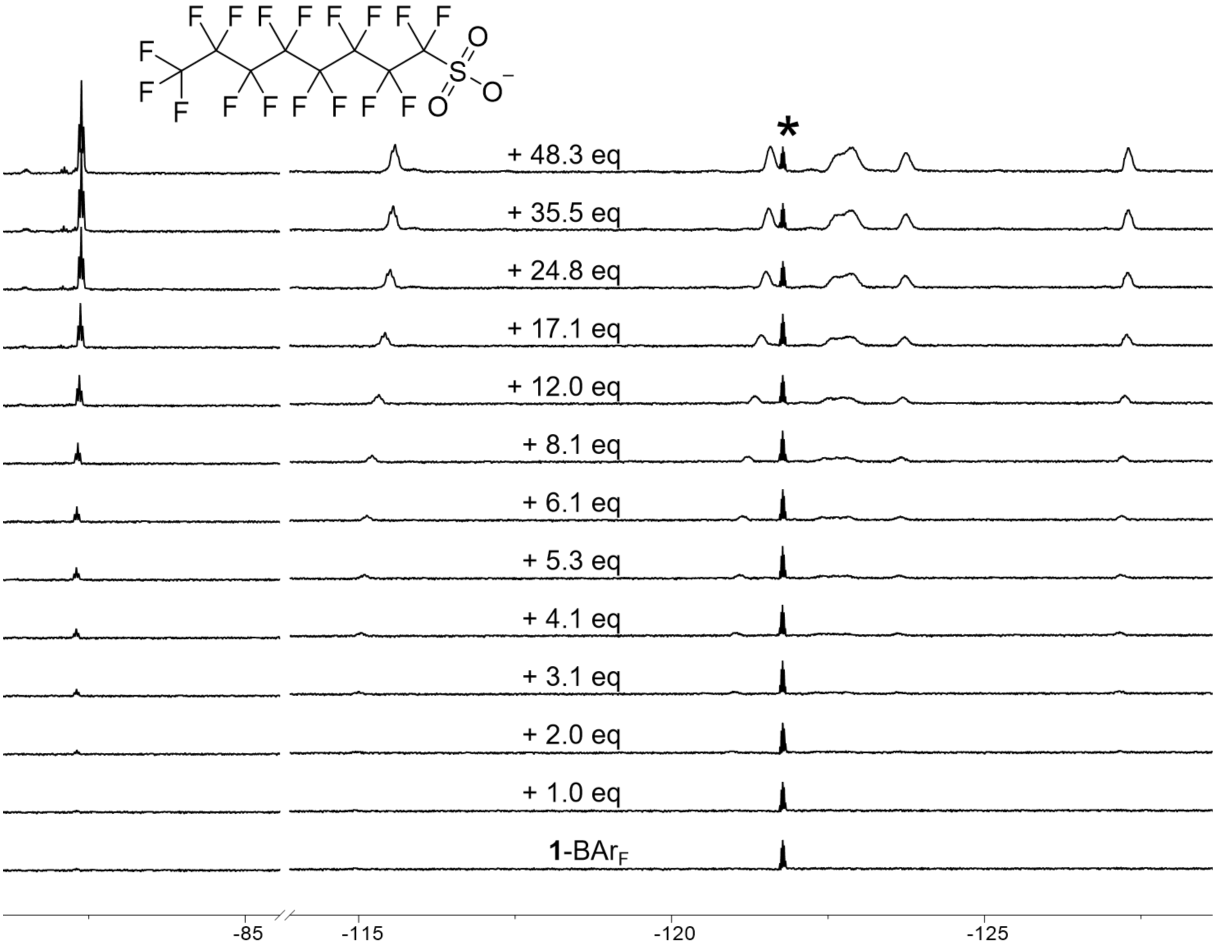

Figure S56. ^{19}F NMR (CD_3OD, 298 K, 376 MHz) titrations of PFOS into a methanol solution of **1**-BAr$_F$ (0.25 mM). The peak of 1,4-difluorobenzene (internal standard) is marked with an asterisk.

4. Adsorption of perfluorosulfonates from water with 1-NO_3

4.1 Removal efficiency of PFOS

1-NO_3 (3.0 mg) was added to an aqueous PFOS solution (670 ppm, 1.0 mL) to form a suspension. After stirring thoroughly for 10 minutes, the suspension was filtered. Then a portion of the filtrate was used for ^{19}F NMR and UHPLC analysis.

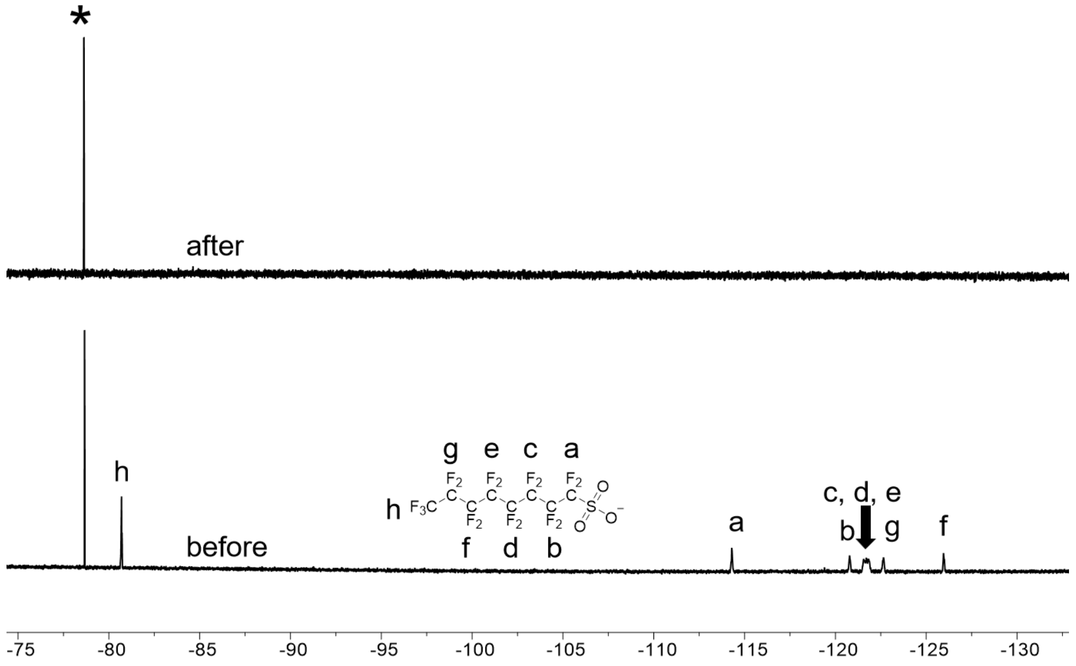

Figure S57. ^{19}F NMR (D_2O, 298 K, 376 MHz) spectra of the PFOS solution with an initial concentration of 670 ppm before and after being treated with **1**-NO_3.The peak of sodium triflate (post-added internal standard) is marked with an asterisk.

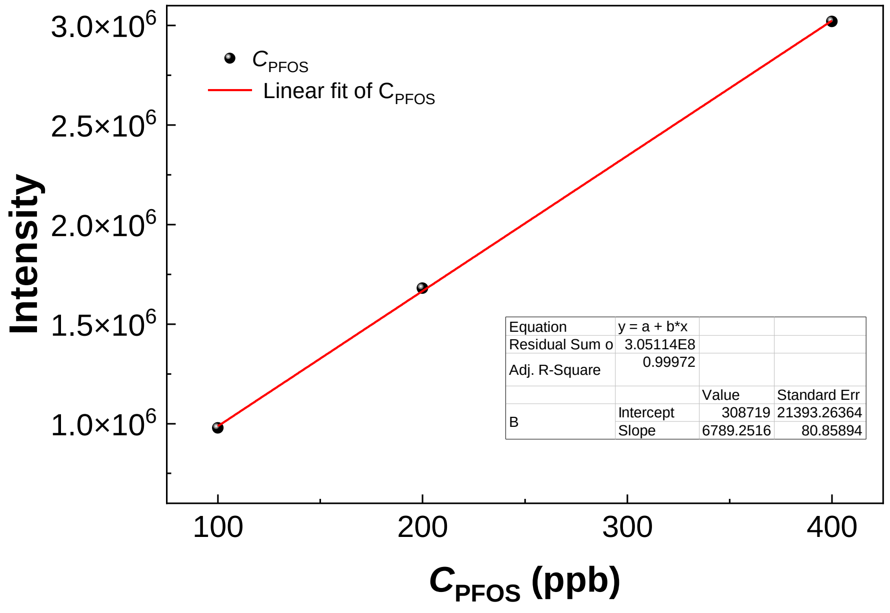

Figure S58. Calibration curve for PFOS quantification.

4.2 Kinetic studies for adsorption of PFOS

Batch kinetic experiments were performed using PFOS solution and **1**-NO_3 at room temperature. Aqueous solutions of PFOS (initial concentration 670 ppm, 1.34 mmol/L, 10 mL) was added to 14 glass vials, followed by the addition of **1**-NO_3 (3.0 mg) to each vial. After continuously stirring for given time intervals (0.5, 1, 1.5, 3, 4.5, 6, 8, 10, 12, 15, 20, 30, 60 and 120 min), the mixture was filtered, and a 500 µL aliquot of the filtrate was taken for ^{19}F NMR analysis, for which sodium triflate was used as the internal standard. All batch kinetic experiments were performed in triplicate.

Adsorption kinetic experiments

The amount of adsorbed PFOS was determined using the following equation:

$$q_t = \frac{(C_0 - C_t)V}{m_{\mathbf{1}}}$$

where q_t (mg g^{-1}) represents the mass of PFOS adsorbed per gram of adsorbent at time t (min), C_0 (ppm, mg) and C_t (ppm) are the initial concentration of PFOS and the concentration at time t (min), respectively, V (L) is the volume of the solution, and $m_{\mathbf{1}}$ (g) is the mass of **1**-NO_3.

The adsorption kinetics can be described by a pseudo-second-order adsorption model, with its linearized form given by the following equation:

$$\frac{t}{q_t} = \frac{t}{q_e} + \frac{1}{k_2 * q_e^2}$$

where q_e (mg g^{-1}) is the amount of PFOS adsorbed at equilibrium. k_2 (g mg^{-1} min^{-1}) is the apparent second-order rate constant, which can be further converted to a value having a unit of g mg^{-1} h^{-1} for convenient comparison.

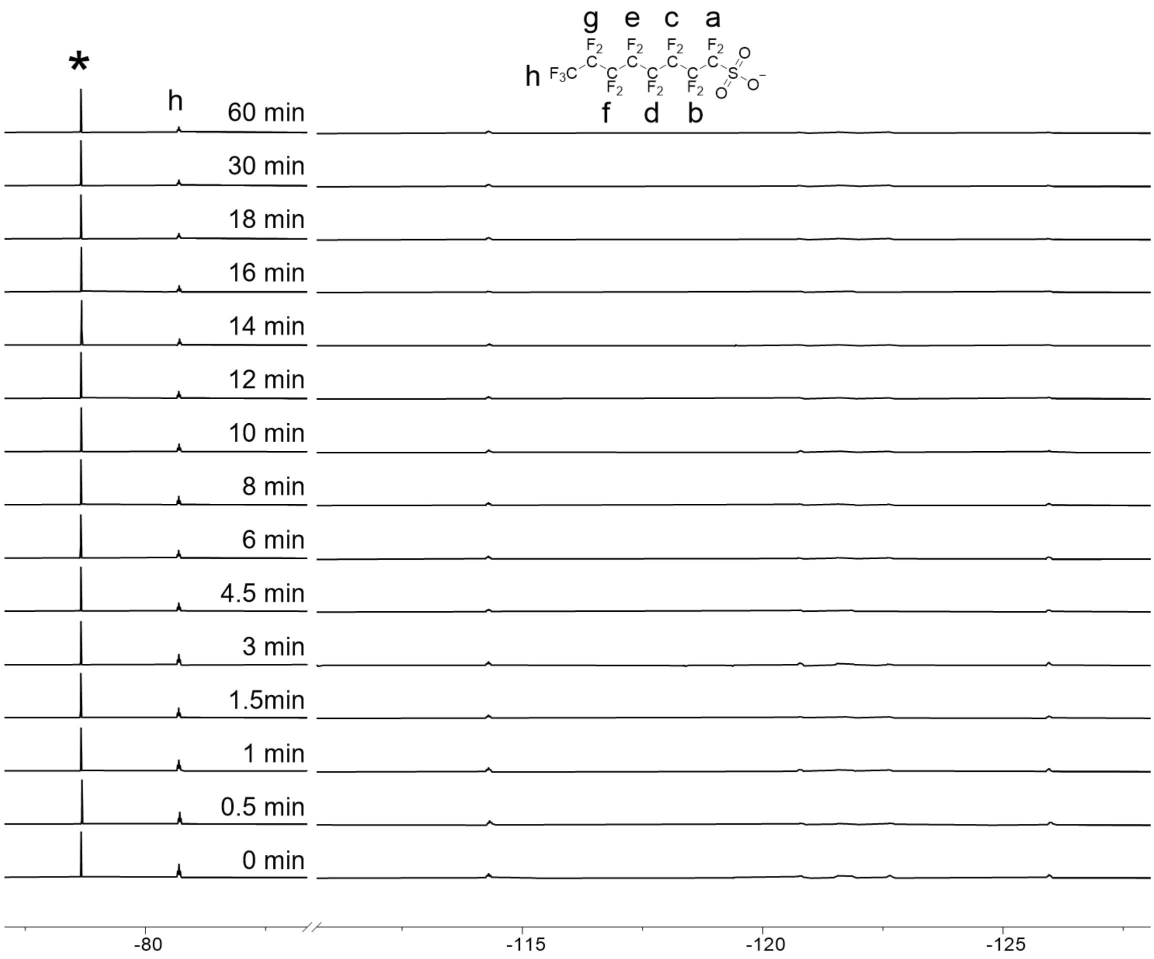

Figure S59. ^{19}F NMR (D_2O, 376 MHz, 298 K) spectra during the kinetic experiments of PFOS adsorption with **1**-NO_3. The peak of sodium triflate (post-added internal standard) is marked with an asterisk.

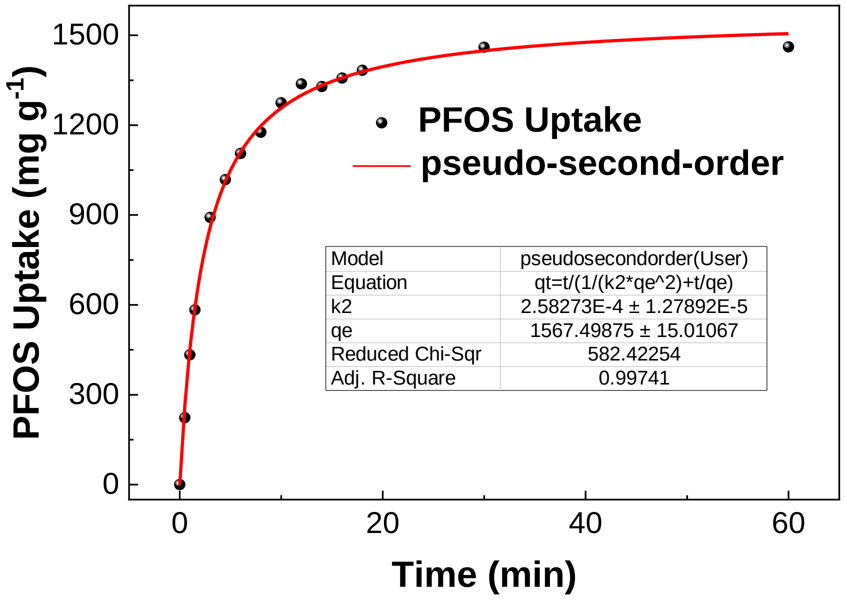

Figure S60. Sorption kinetics of PFOS into **1**-NO_3 with an initial concentration of 670 ppm, fitted with a pseudo-second-order model.

4.3 Isotherm adsorption experiments for PFOS

The isotherm experiments were conducted at room temperture. PFOS solutions (10 mL; 50, 100, 150, 200, 300, 400, 500, and 670 ppm) were added to 25 mL glass vials, followed by the addition of **1**-NO_3 (3.0 mg) to each vial. The suspensions were constantly stirred for 1 h to reach equilibrium, and then filtered. The filtrate was analyzed using ^{19}F NMR. All batch experiments were performed in triplicate.

The experimental data was fitted to the Langmuir isotherm models as follows:

$$\frac{1}{q_e}=\frac{1}{q_m}+\frac{1}{C_e q_m K_L}$$

Where q_e (mg g^{-1}) is the mass of FPOS adsorbed at equilibrium. q_m (mg g^{-1}) is the maximum adsorption capacity of the adsorbent. C_e (mg L^{-1}) is the residual PFOS concentration at equilibrium. K_L (L mg^{-1}) is the equilibrium constant.

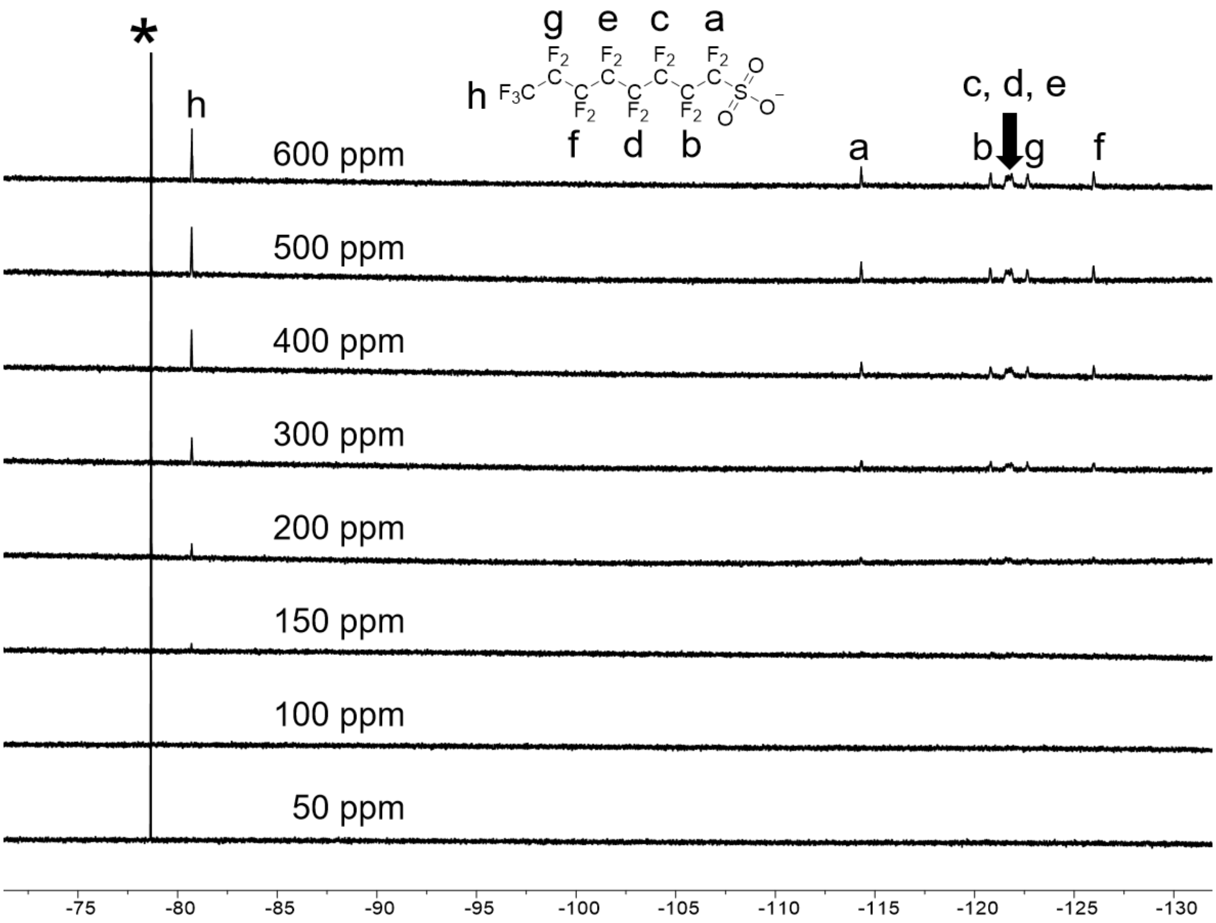

Figure S61. ^{19}F NMR (D_2O, 298 K, 376 MHz) spectra of the filtrates obtained after stirring **1**-NO_3 and PFOS solutions with different initial concentrations for 1 h. The peak of sodium triflate (post-added internal standard) is marked with an asterisk.

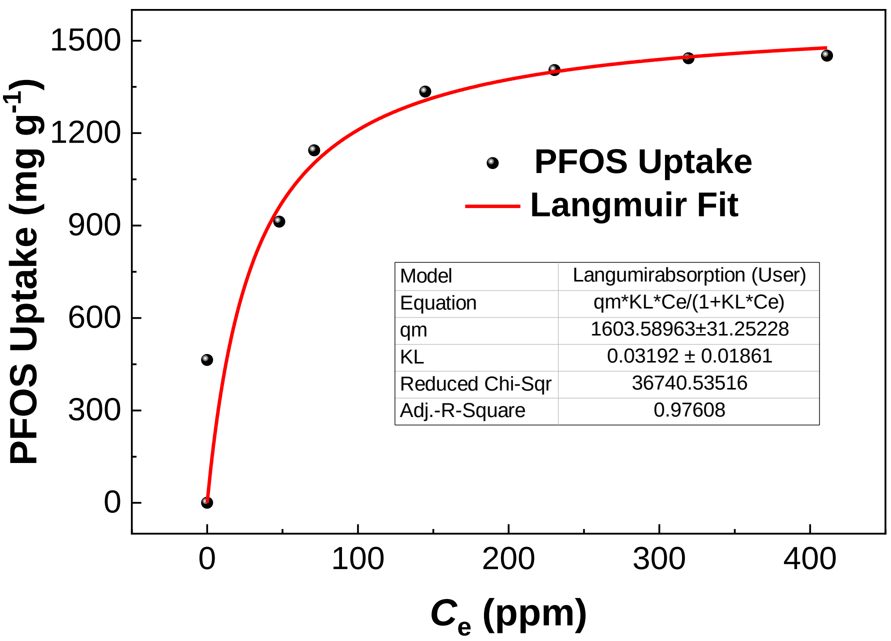

Figure S62. PFOS adsorption isotherm of **1**-NO_3 fitted with the Langmuir model.

4.4 Interference experiments for adsorption of PFOS

An aqueous solution (10 mL) containing 670 ppm (1.34 mmol/L) PFOS and 20 equivalents of an inorganic salt (26.8 mmol/L, KNO_3, $Mg(NO_3)_2$, KCl, $CaCl_2$, Na_2SO_4, or $Zn(NO_3)_2$) was mixed with 3.0 mg of **1**-NO_3 and stirred for 1 h at room temperature. The suspension was filtered, and the filtrate was analyzed using ^{19}F NMR with sodium triflate as the post-added internal standard for the quantification of the adsorbed PFOS.

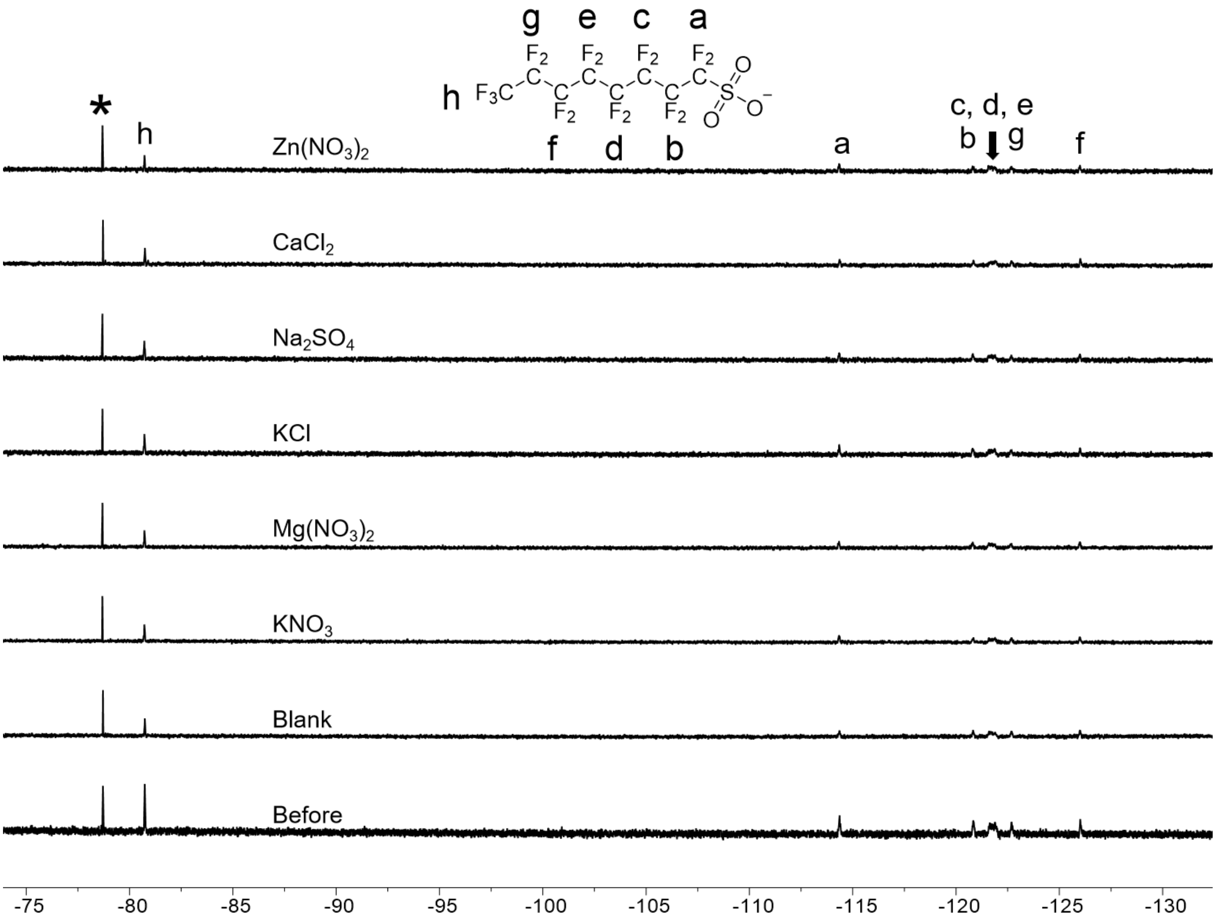

Figure S63. ^{19}F NMR (D_2O, 298 K, 376 MHz) spectra of the solutions after PFOS adsorption in the absence or presence of different interferring species. The peak of sodium triflate (post-added internal standard) is marked with an asterisk.

4.5 Mechanism Investigation

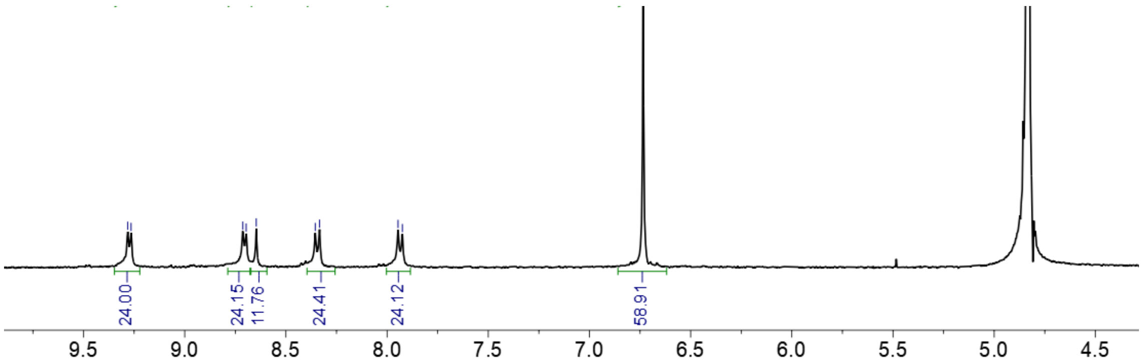

Figure S64. ^{1}H NMR (CD_3OD, 298 K, 400 MHz) spectrum of the solid isolated after PFOS adsorption (**1**-NO_3 3.0 mg, PFOS 670 ppm in 10 mL, 1 h).

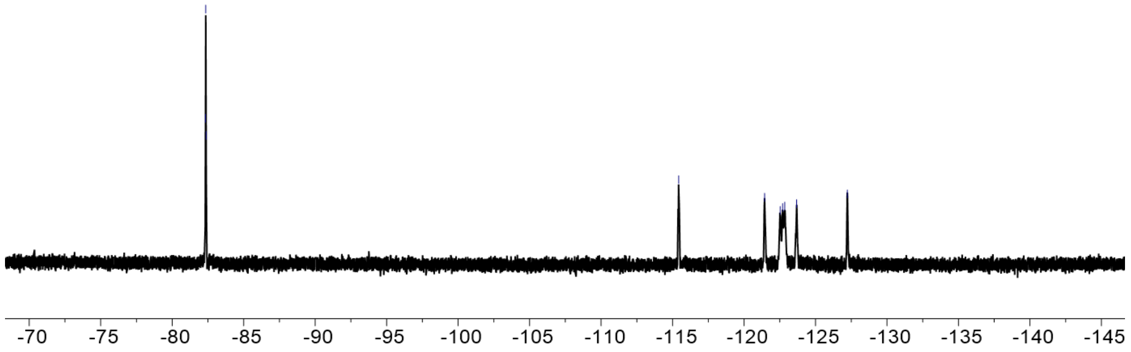

Figure S65. ^{19}F NMR (CD_3OD, 298 K, 376 MHz) spectrum of the solid isolated after PFOS adsorption (**1**-NO_3 3.0 mg, PFOS 670 ppm in 10 mL, 1 h).

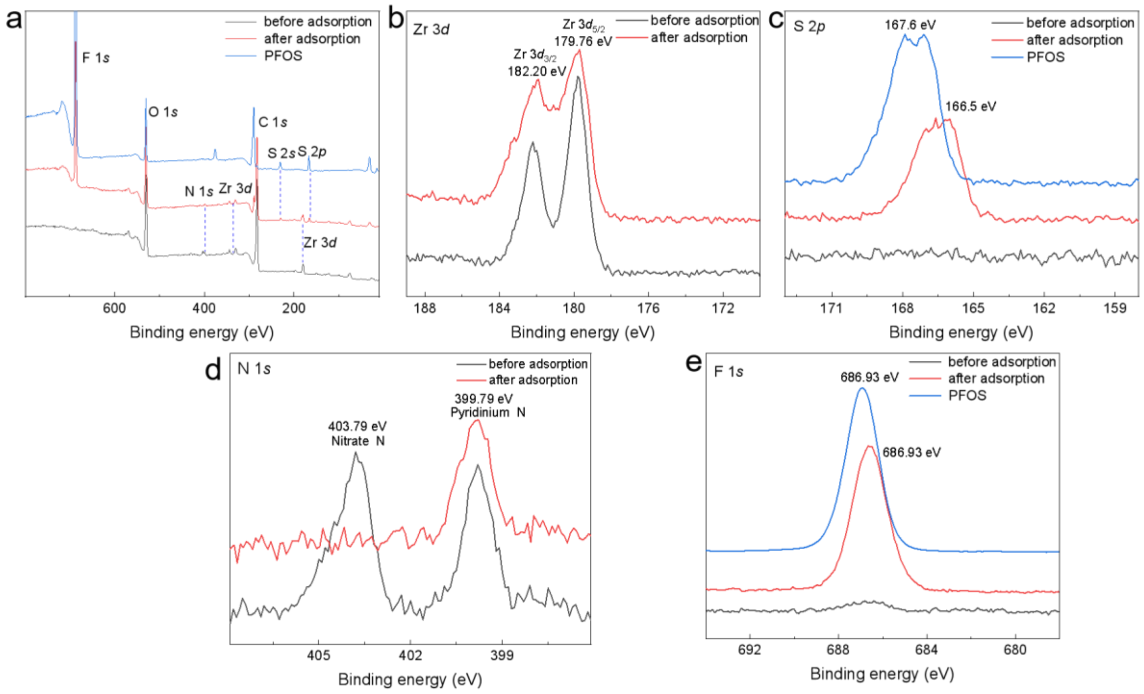

Figure S66. (a) XPS spectra of KPFOS, and 1-NO_3 before and after PFOS adsorption. (b – e) Spectra of S Zr 3*d* (b), S 2*p* (c), N 1*s* (d), and F 1*s* (e).

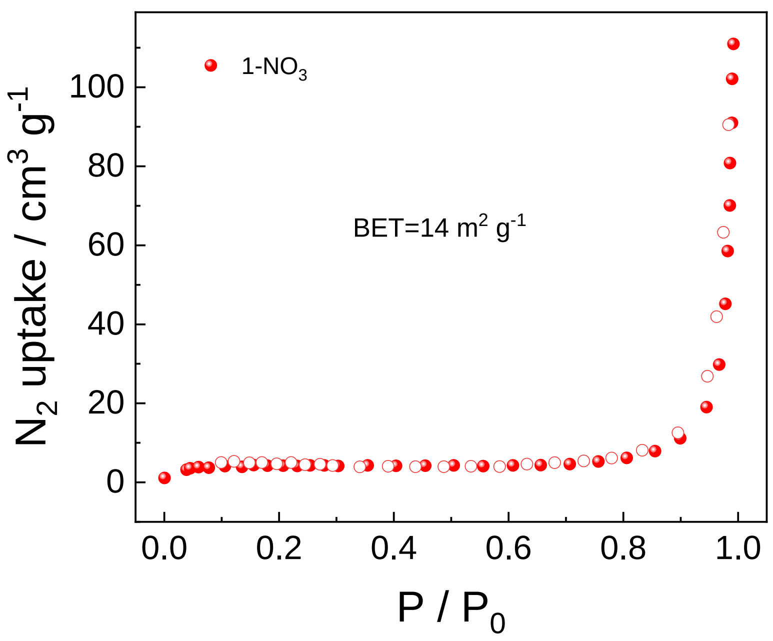

Figure S67. Nitrogen adsorption– desorption isotherms at 77 K of **1**-NO_3.

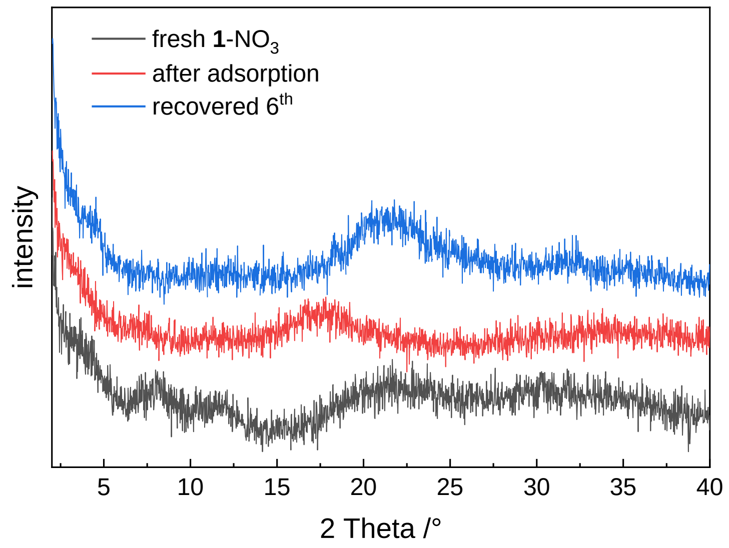

Figure S68. XRD patterns of fresh, PFOS-adsorbed, and regenrated samples of the adsorbent (**1-**NO_3). The essentially amorphous nature makes it difficult to extract precise information about the solid-state transformation from the XRD data. However, the main hump observed around $2\theta = 22°$ for the fresh sample shifted to 17.5° after adsorption. This suggests that the amorphous adsorbent underwent some kind of structural transformation after adsorption, as expected for nonporous or non-surficial adsorption. Interestingly, the main hump returned to 22° after regeneration, indicative of recovery of the main structural features of the adsorbent. There are some differences in the weak bumps in the 7.5 - 12° range, which indicate some kind of structural changes after use. Nevertheless, the changes do not significantly affect the adsorption performance, as suggested by the recycle tests.

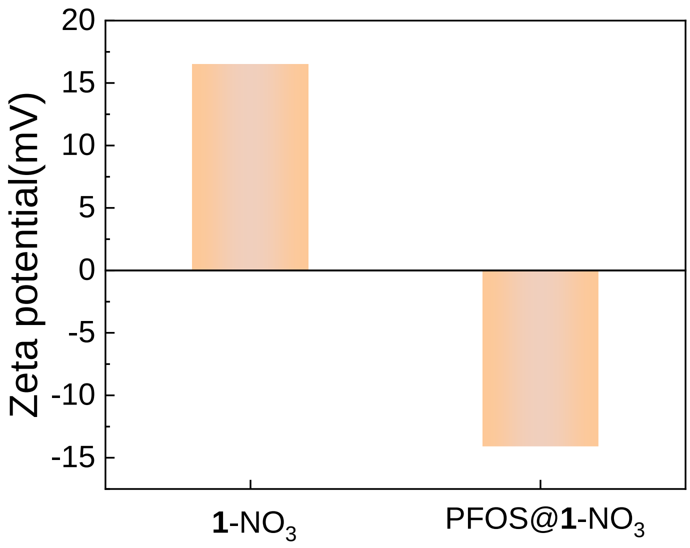

Figure S69. Zeta potential of **1**-NO_3 before and after adsorption.

4.6 Regeneration and reuse of 1-NO_3

1-NO_3 was regenerated by stirring the solid after PFOS adsorption in a KNO_3 solution (0.10 M, 1.0 mL) in methanol/water (10:1, v/v) for 1 h at room temperature. The solid was filtered, washed with the mixed solvent, dried in air, and then used for the next run of PFOS adsorption (**1**-NO_3 3.0 mg (weighed before the first run), PFOS 670 ppm (10 mL), 1 h).

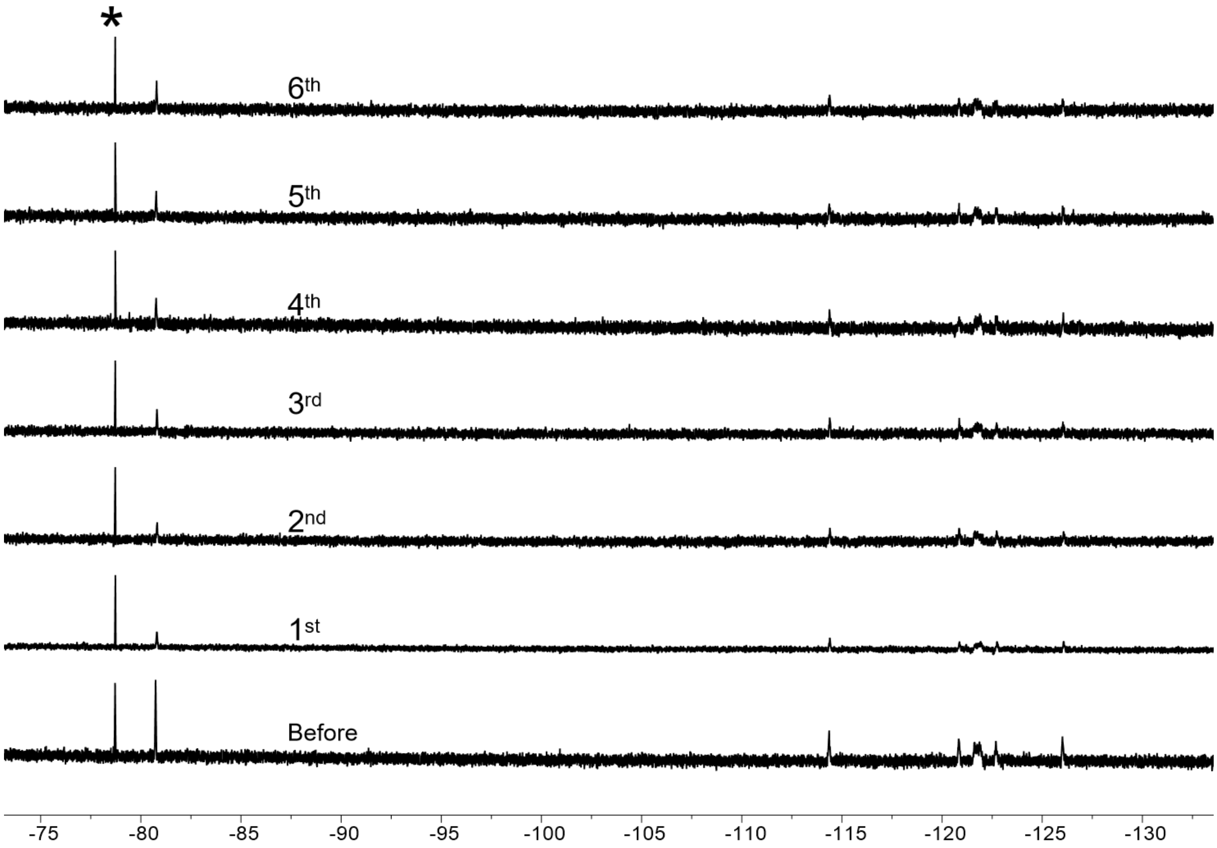

Figure S70. ^{19}F NMR (D_2O, 298 K, 376 MHz) spectra of the PFOS solutions in six successive cycles of adsorption with **1**-NO_3. The peak of sodium triflate (post-added internal standard) is marked with an asterisk.

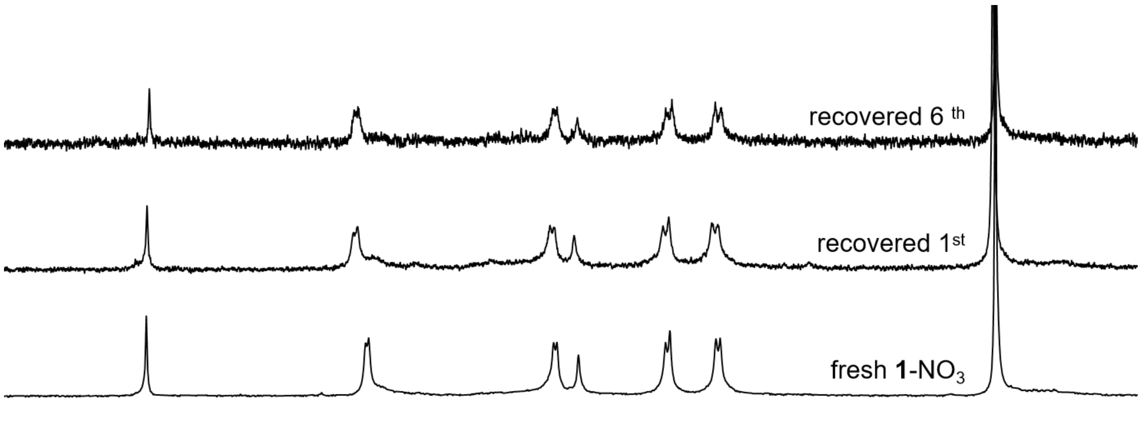

Figure S71. ^{1}H NMR (d_6-DMSO, 298 K, 400 MHz) spectra of fresh, recovered 1st and recovered 6th **1**-NO_3.

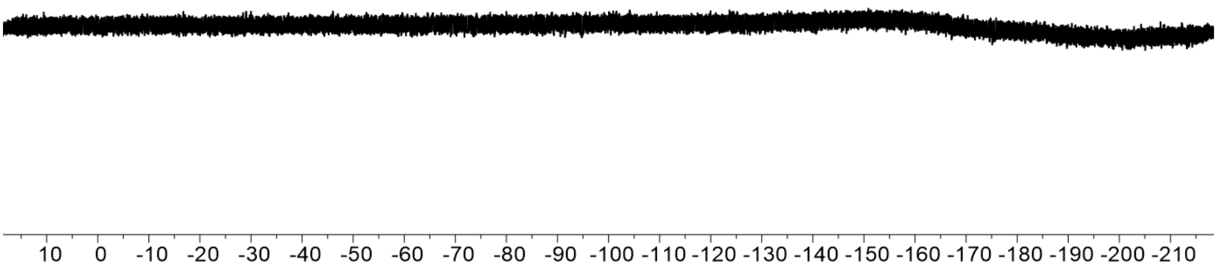

Figure S72. ^{19}F NMR (d_6-DMSO, 298 K, 376 MHz) spectrum of recovered **1**-NO_3.

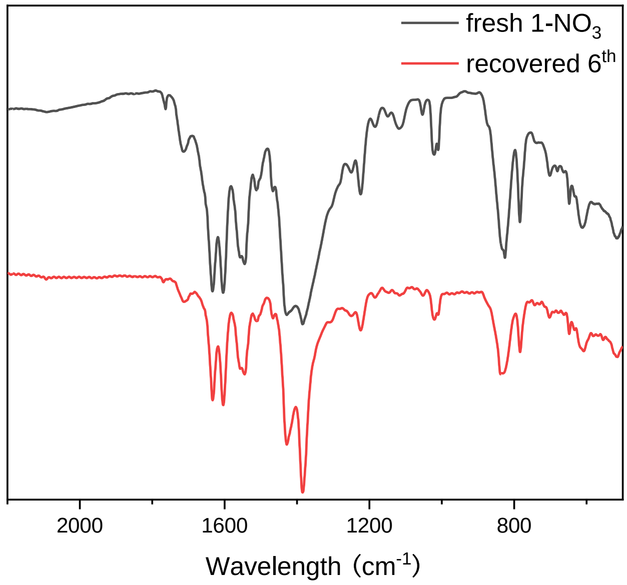

Figure S73. FT-IR spectra of **1**-NO_3 fresh (black) and recovered 6th (red).

4.7 Removal efficiency of PFBS and PFHxS

The experiments were performed following the procedure described in Section 4.1, using PFBS and PFHxS (initial concentration 800 ppm) in the place of PFOS.

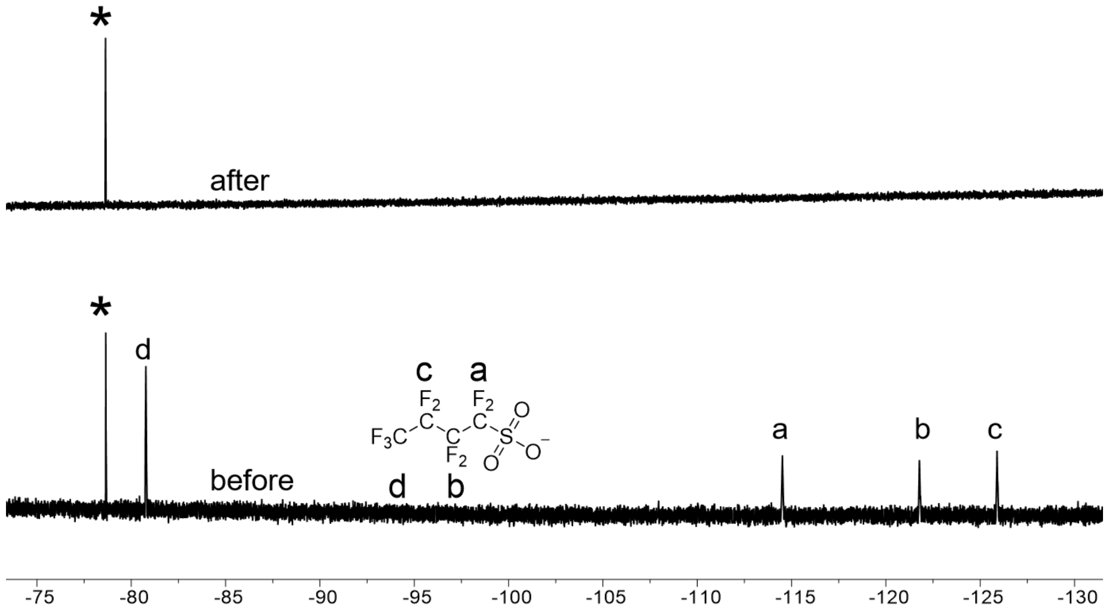

Figure S74. ^{19}F NMR (D_2O, 298 K, 376 MHz) spectra of the PFBS solution with an initial concentration of 800 ppm before and after being treated with **1**-NO_3.The peak of sodium triflate (post-added internal standard) is marked with an asterisk.

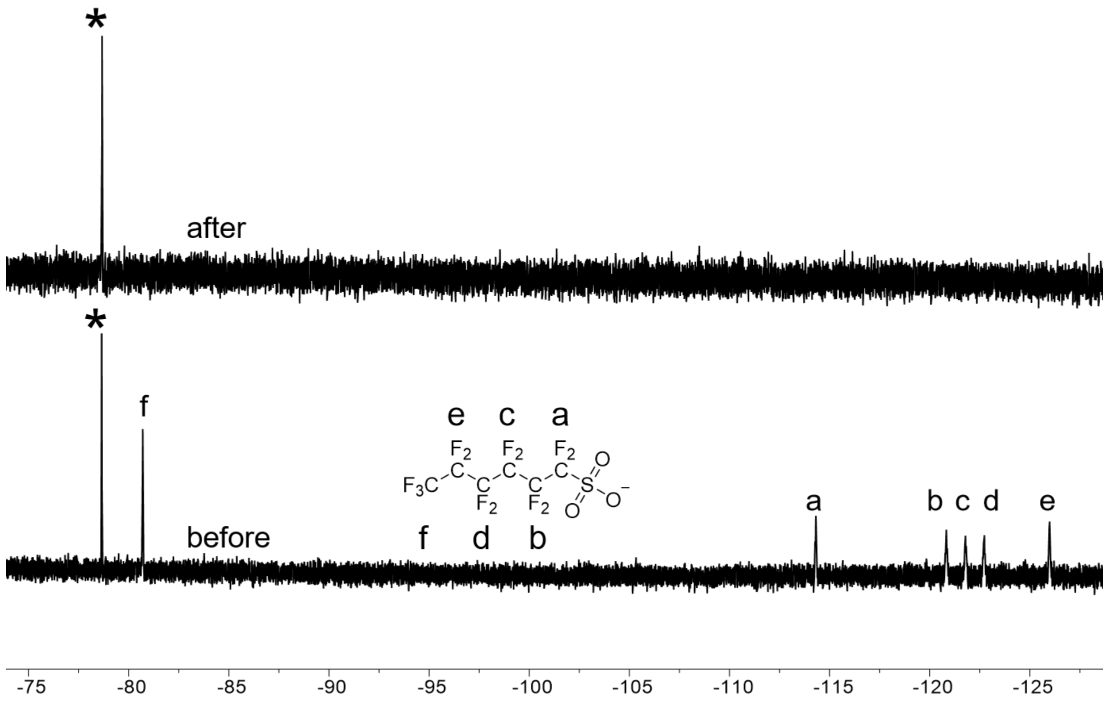

Figure S75. ^{19}F NMR (D_2O, 376 MHz, 298 K) spectra of the PFHxS solution with an initial concentration of 800 ppm before and after being treated with **1**-NO_3. The peak of sodium triflate (post-added internal standard) is marked with an asterisk.

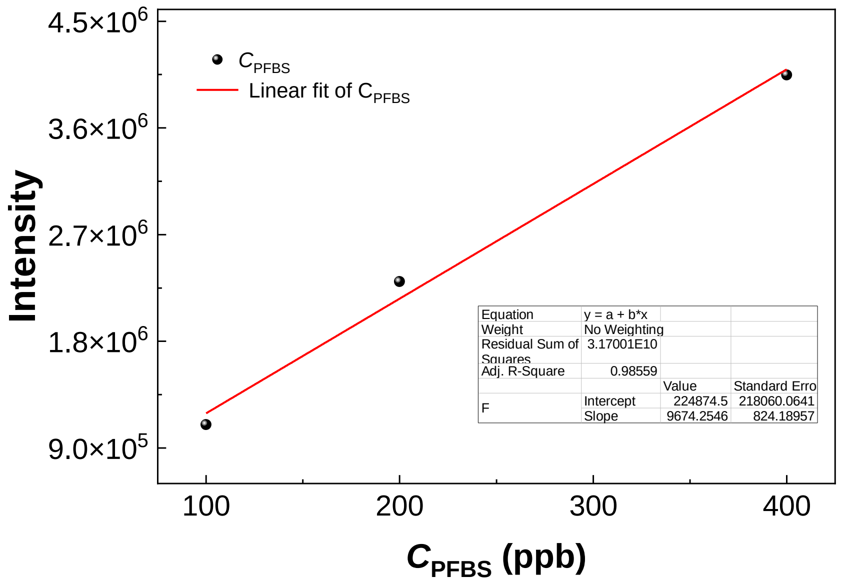

Figure S76. Calibration curve for PFBS quantification.

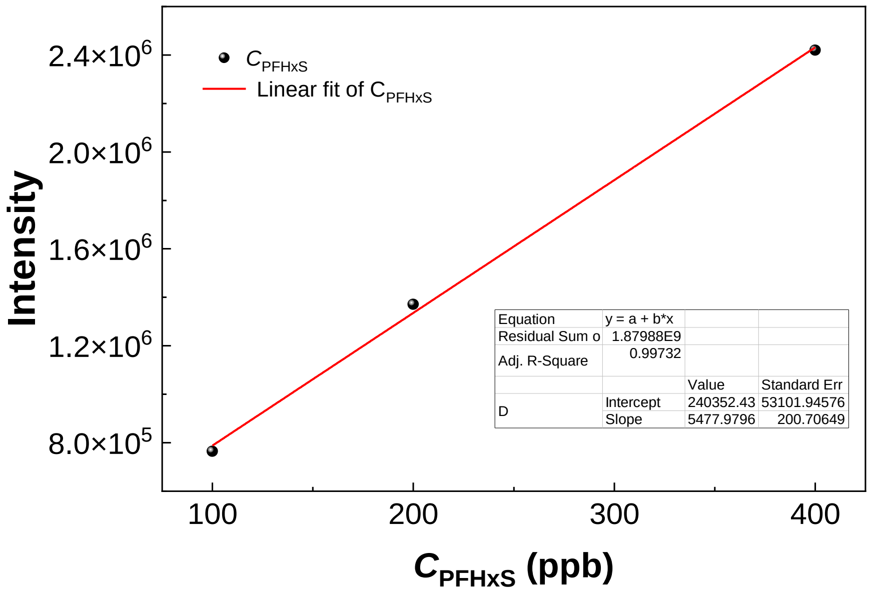

Figure S77. Calibration curve for PFHxS quantification.

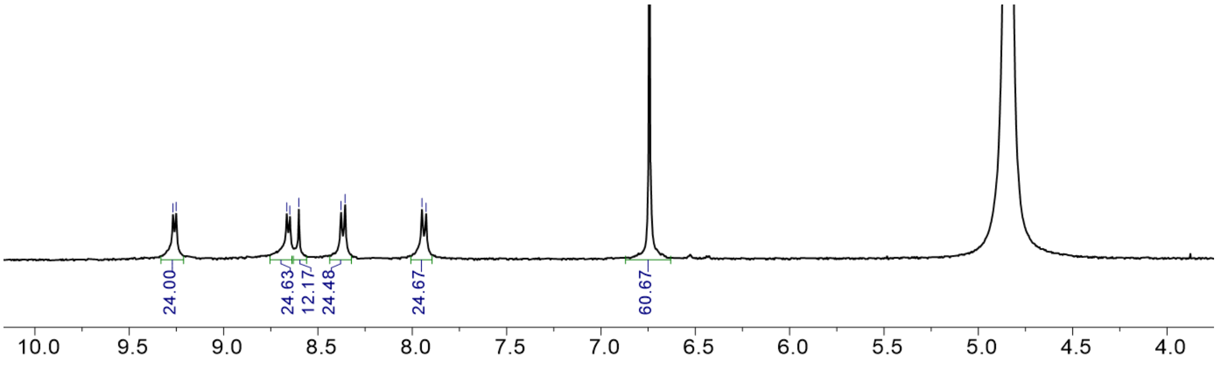

Figure S78. ^{1}H NMR (CD_3OD, 298 K, 400 MHz) spectrum of **1**-NO_3 after the adsorption of PFBS.

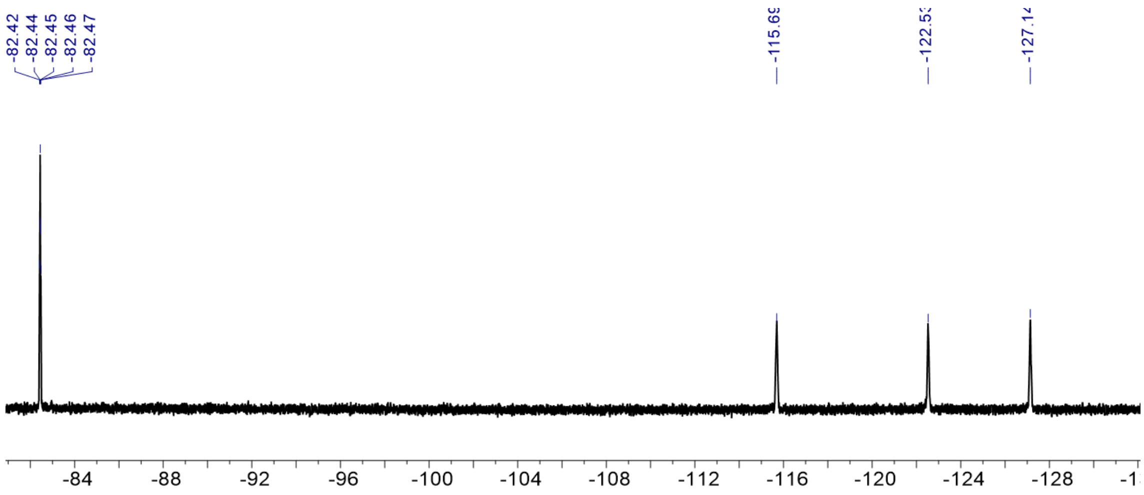

Figure S79. ^{19}F NMR (CD_3OD, 298 K, 376 MHz) spectrum of **1**-NO_3 after the adsorption of PFBS.

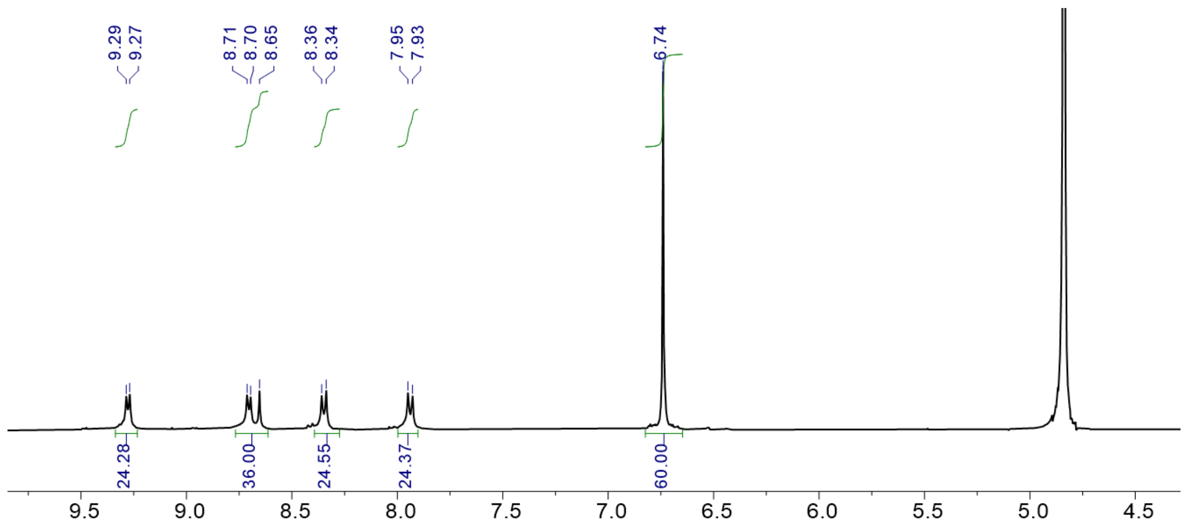

Figure S80. ^{1}H NMR (CD_3OD, 298 K, 400 MHz) spectrum of **1**-NO_3 after the adsorption of PFHxS.

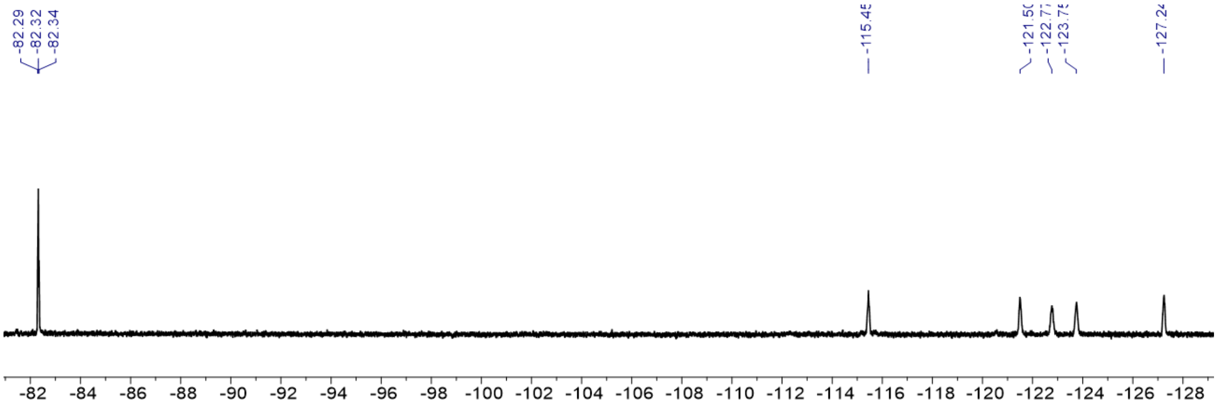

Figure S81. ^{19}F NMR (CD_3OD, 298 K, 376 MHz) spectrum of **1**-NO_3 after the adsorption of PFHxS.

4.8 Kinetic studies for adsorption of PFBS and PFHxS

The experiments were performed following the procedure described in Section 4.2, using PFBS and PFHxS in the place of PFOS, with the same initial concentration of 1.34 mmol/L.

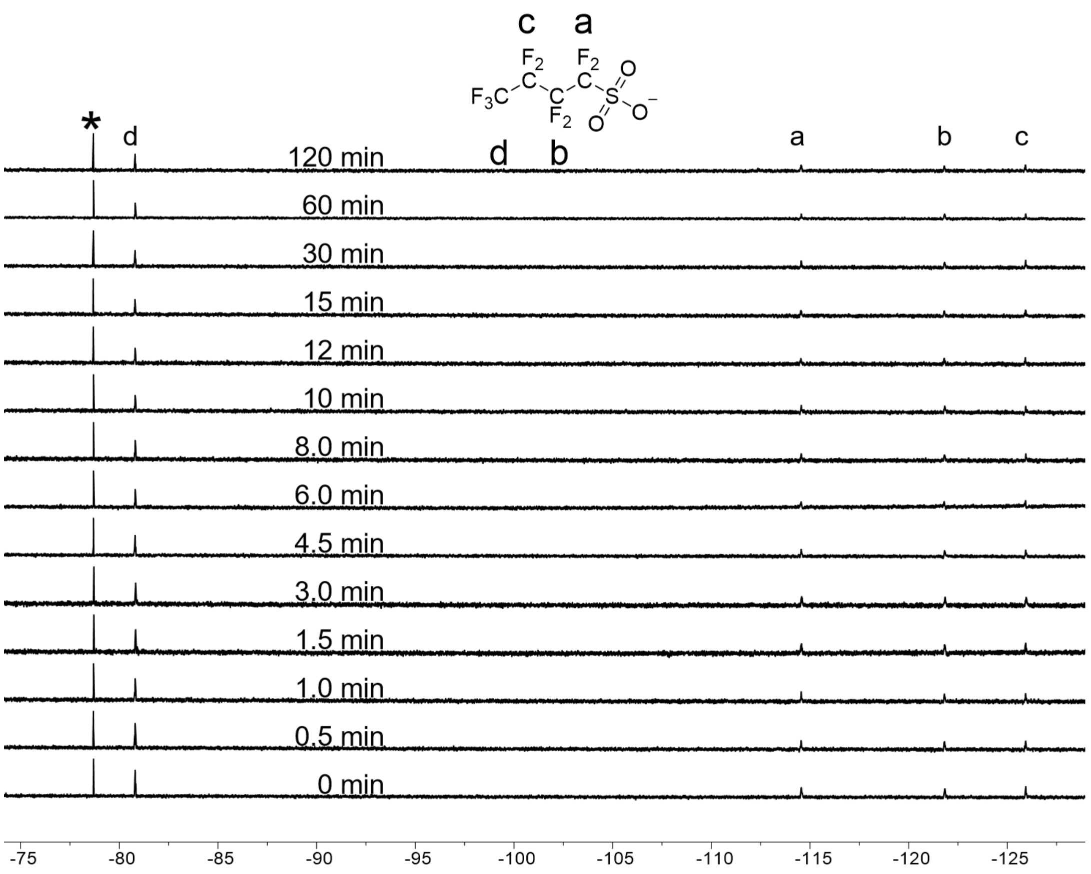

Figure S82. ^{19}F NMR (D_2O, 376 MHz, 298 K) spectra during the kinetic experiments of PFBS adsorption with **1**-NO_3. The peak of sodium triflate (post-added internal standard) is marked with an asterisk.

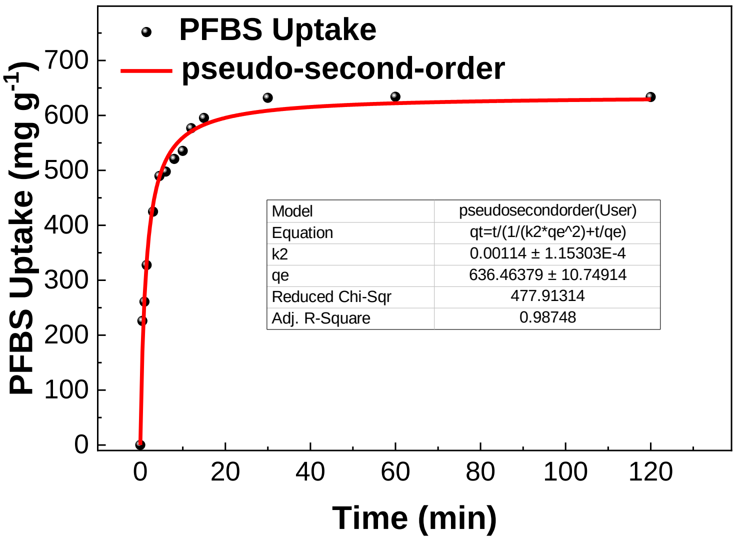

Figure S83. Sorption kinetics of PFBS into **1**-NO_3 with an initial concentration of 400 ppm, fitted with a pseudo-second-order model.

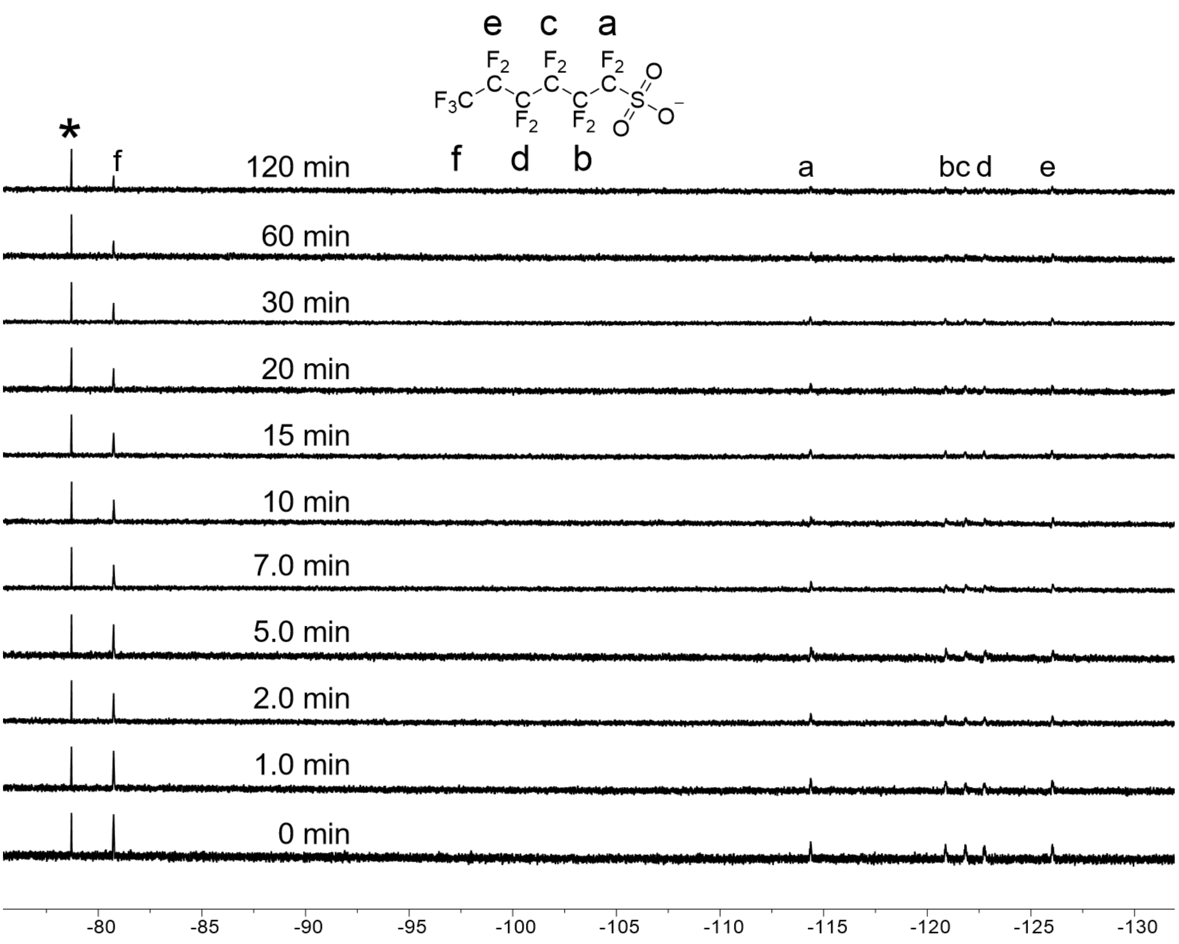

Figure S84. ^{19}F NMR (D_2O, 376 MHz, 298 K) spectra during the kinetic experiments of PFBS adsorption with **1**-NO_3. The peak of sodium triflate (post-added internal standard) is marked with an asterisk.

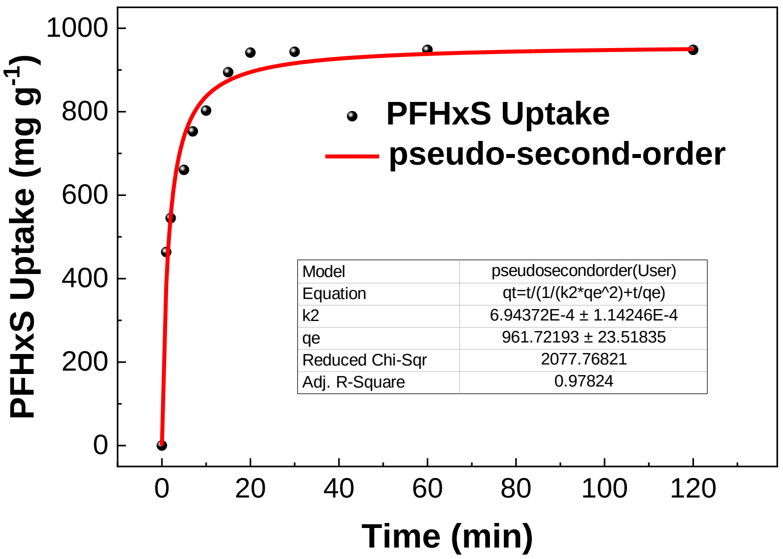

Figure S85. Sorption kinetics of PFHxS into **1**-NO_3 with an initial concentration of 550 ppm, fitted with a pseudo-second-order model.

4.9 Isotherm adsorption experiments for PFBS and PFHxS

The experiments were performed following the procedure described in Section 4.3, using PFBS and PFHxS in the place of PFOS.

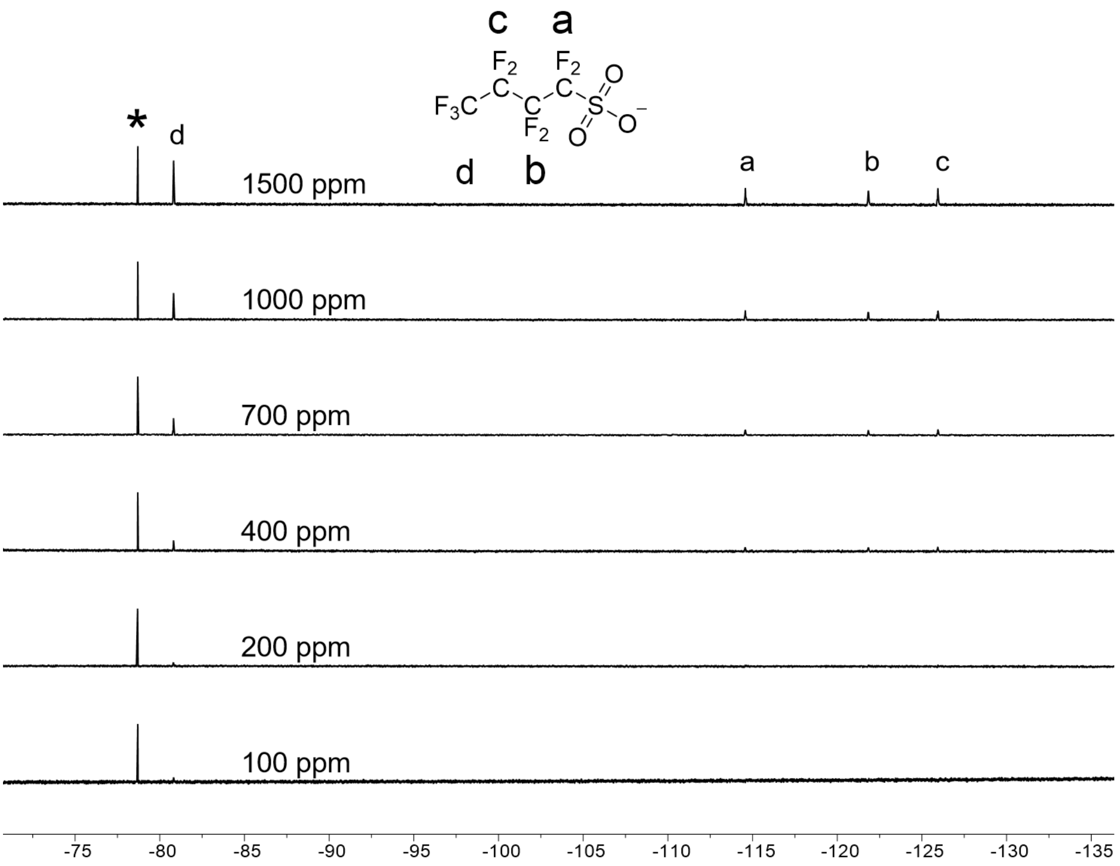

Figure S86. ^{19}F NMR (D_2O, 298 K, 376 MHz) spectra of the filtrates obtained after stirring **1**-NO_3 and PFBS solutions with different initial concentrations for 1 h. The peak of sodium triflate (post-added internal standard) is marked with an asterisk.

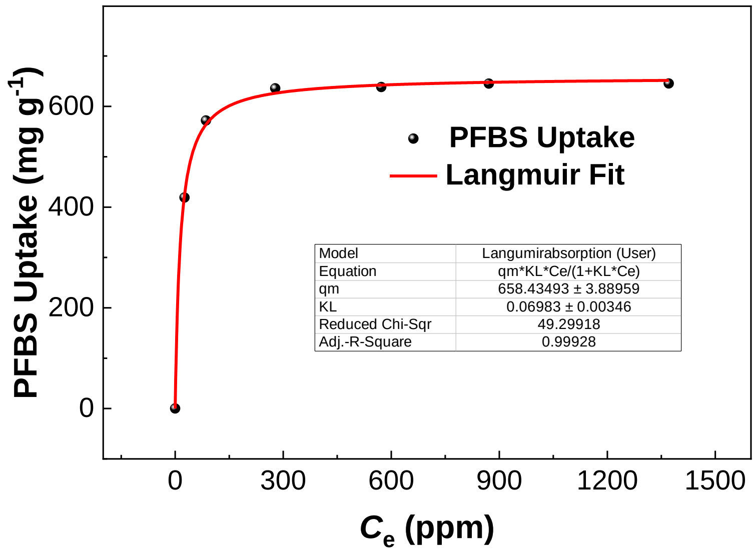

Figure S87. PFBS adsorption isotherm of **1**-NO_3 fitted with the Langmuir model.

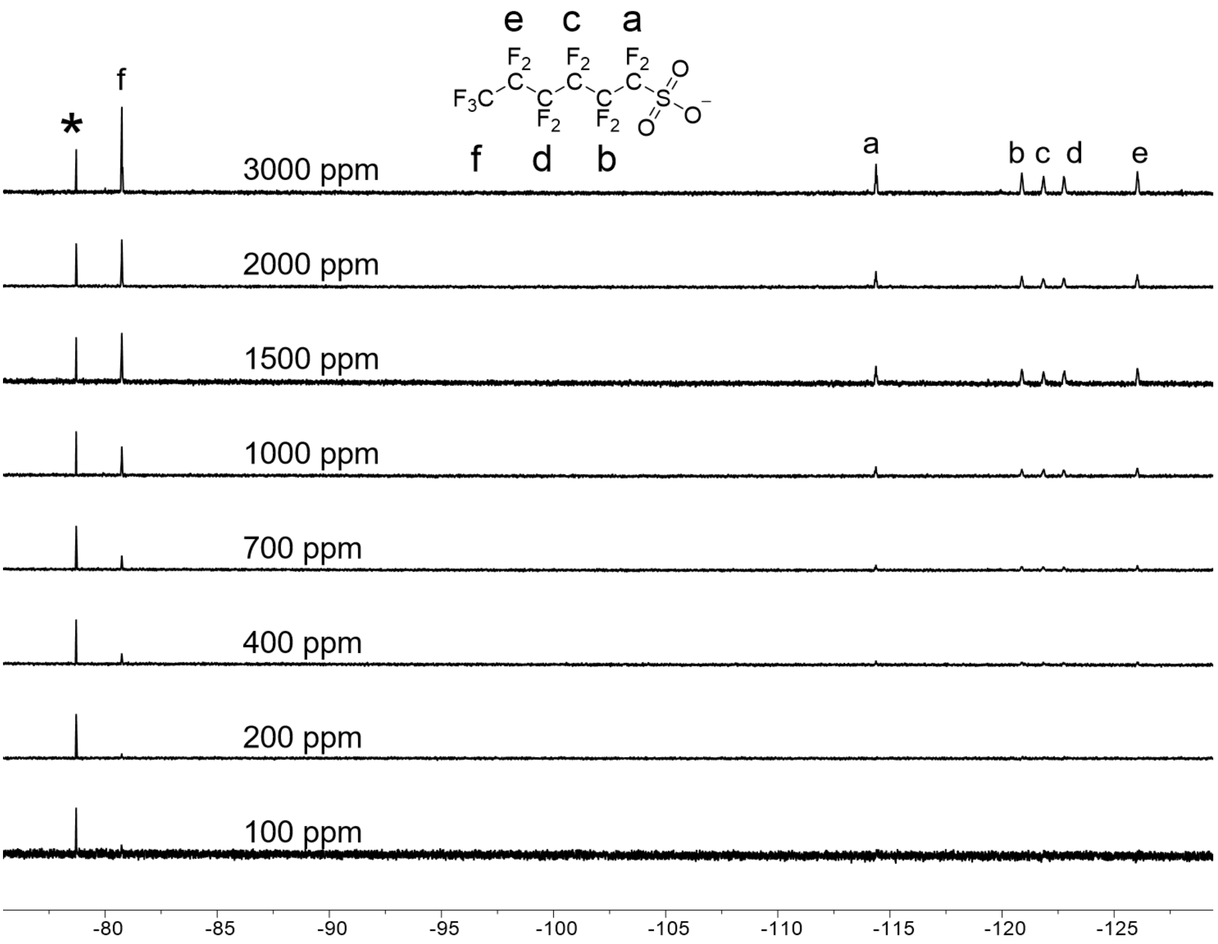

Figure S88. ^{19}F NMR (D_2O, 298 K, 376 MHz) spectra of the filtrates obtained after stirring **1**-NO_3 and PFHxS solutions with different initial concentrations for 1 h. The peak of sodium triflate (post-added internal standard) is marked with an asterisk.

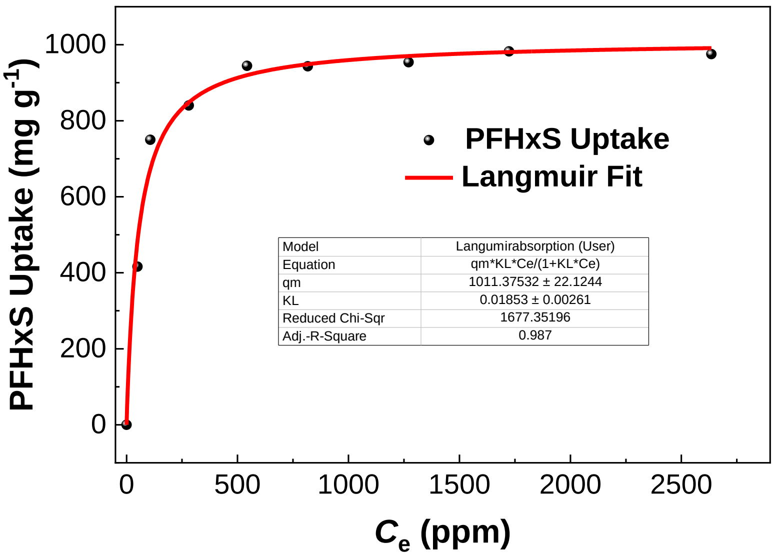

Figure S89. PFHxS adsorption isotherm of **1**-NO_3 fitted with the Langmuir model.

5. Computational calculations

5.1. Structure optimization

Structures of cage **1**, free guests, and host–guest complexes of **1** with **G15** and PFOS were optimized by a semiempirical extended tight-binding method[4] at the GFN2-xTB level of theory[5] with the xtb program version6.5.1.[6]

The strong repulsion of positive charges on faces and vertices led to an excessively bloated cage after optimization in vacuum (dielectric constant or relative permittivity $\varepsilon_r = 1$) (Figure S90, left). Using an implicit solvation based on the analytical linearized Poisson–Boltzmann (ALPB) model[7] to screen charges allowed to mitigate this ionic repulsion. Solvents tested were hexane ($\varepsilon_r = 1.88$), chloroform ($\varepsilon_r = 4.81$), dichloromethane ($\varepsilon_r = 8.93$), acetone ($\varepsilon_r = 20.7$), methanol ($\varepsilon_r = 32.7$), and water ($\varepsilon_r = 80.2$). Apolar solvents with small dielectric constants hexane, chloroform, and dichloromethane still led to significantly bloated structures with bent faces, indicating an insufficient charge screening. Highly polar solvents methanol and water led to the complete collapse of the cage and failure of the optimization process due to overly screened charges, favoring excessive π-stacking between faces (Figure S90, right). Acetone as an intermediate polar solvent led to a tetrahedral cage with close to planar faces matching the optimized structure of isolated ligands (Figure S90, middle), indicating an adequate charge screening for the implicit solvation with acetone.

The high charge density on cage **1** faces also lead to challenging modelling of host–guest complexes. Indeed, the close proximity of anionic sulfonate and carbon atoms with high partial positive charge led to the formation of covalent SO_3–C bonds that are not representative of the real reactivity and interactions observed experimentally. To suppress this behavior, it was necessary to stabilize the cationic charges of the cage faces by adding anions around the cage. Sufficient BF_4^- anions were added close to the external side of the faces to equilibrate all charges on the faces of the cage (i.e. 12 BF_4^- for empty cage, 10 BF_4^- with one **G15**, 8 BF_4^- with two **G15**, 6 BF_4^- with three **G15**, 11 BF_4^- with one PFOS, and 10 BF_4^- with two PFOS guests, see Figures S91 and S92).

For free and bound PFOS guests, the conformational landscape of PFOS was considered using linear or various curled conformations that would shorten the total length while widening PFOS guests. PFOS guests with a linear conformation lead to the most stable complexes, likely due to the steric repulsion caused by gauche conformations in curled PFOS. Similarly to G15 inclusion complexes, the sulfonate head of an included PFOS binds on an open edge of **1**, between cationic faces, forming 2 C–H···O–S hydrogen bonds with a H···O distance of 2.0 Å. While a single PFOS molecule does not significantly fills the cavity of **1** (PFOS V_{vdw} = 261 Å^3 for a cavity volume of 1069 Å^3), it occupies the center of the cavity and does not leave enough space for the inclusion of a second PFOS guest (Figure 92). Accordingly, a second PFOS binds with its sulfonate head on another edge binding site but with the perfluoroalkyl chain protruding outside the cage. In the dynamic solution state, the second protruding PFOS guest may experience secondary interactions between its perfluoroalkyl chain and the cationic cage faces when adopting curled conformations but static optimized structure with different conformations indicated that the host–guest complex with non-interacting linear perfluoroalkyl chain was more stable by >15 kJ/mol.

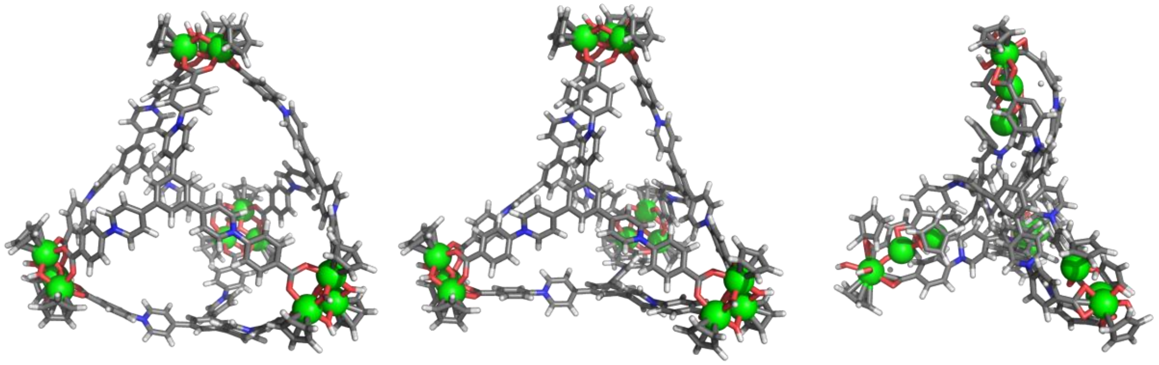

Figure S90. GFN2-xTB-optimized structure of cage **1** in vacuum (left), acetone (middle), methanol (right, optimization failure).

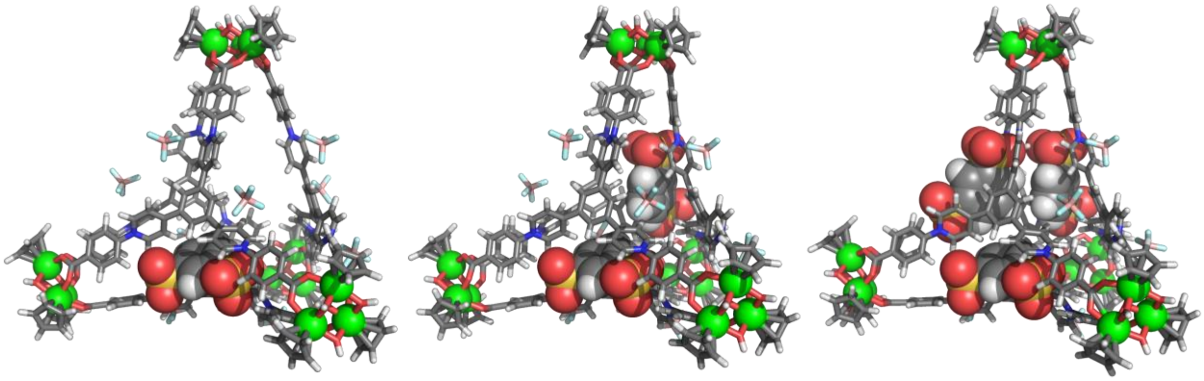

Figure S91. GFN2-xTB-optimized structure of 1:1, 1:2, and 1:3 host–guest complexes of cage **1** with **G14**.

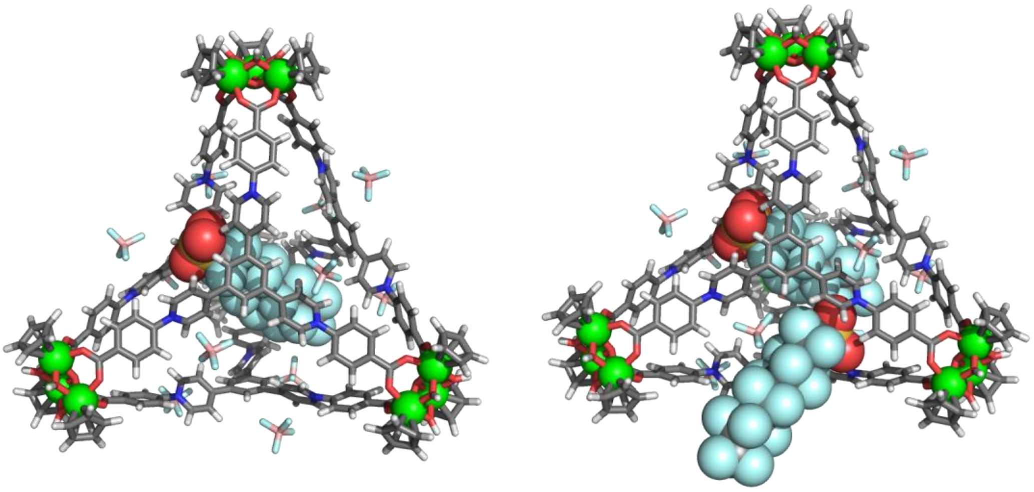

Figure S92. GFN2-xTB-optimized structure of 1:1 (left) and 1:2 (right) host–guest complexes of cage **1** with PFOS.

5.2. Volume calculations

Van der Waals volume of guests and cavity volume of the cage were calculated from the GFN2-xTB-optimized structures with MoloVol 1.2.0.[8] Guest structures used correspond to their gas phase models. The cage structure used corresponds to the model with acetone implicit solvation and 12 stabilizing external BF_4^- anions. Calculations were run in the single probe mode with a probe radius of 2.7 Å, a grid resolution of 0.1 Å, and default values for other parameters. Smaller probes could exit through the cage apertures. The cavity volume of cage **1** was calculated to be 1069 $Å^3$ (see Figure 1c of the main text).

5.3. Molecular electrostatic potential calculations

To generate surface electrostatic potentials, atomic charges were calculated at the PM7 level of theory[9] using the program MOPAC (version 22.0.5)[10] with a single SCF calculation and GRAPHF keyword on the GFN2-xTB-optimized structures of cage **1** and PFOS with implicit solvation model in acetone. Molecular electrostatic potential (MEP) were displayed at the van der Waals surface using Jmol (version 16.3.1) (see Figure 5c of the main text).[11] The color scheme represents surface potential charges (in atomic units, a.u.) based on atomic net charges.

6. References

1. Wu, G.; Bae, Y. J.; Olesinska, M.; Anton-Garcia, D.; Szabo, I.; Rosta, E.; Wasielewski, M. R.; Scherman, O. A., Controlling the structure and photophysics of fluorophore dimers using multiple cucurbit[8]uril clampings. *Chem. Sci.* **2020,** *11*, 812-825.
2. Camdzic, D.; Welgama, H. K.; Crawley, M. R.; Avasthi, A.; Cook, T. R.; Aga, D. S., Rapid Capture of Per- and Polyfluoroalkyl Substances Using a Self-Assembling Zirconium-Based Metal-Organic Cage. *ACS Appl. Eng. Mater.* **2023,** *2*, 87-95.
3. http://supramolecular.org/.
4. Bannwarth, C.; Caldeweyher, E.; Ehlert, S.; Hansen, A.; Pracht, P.; Seibert, J.; Spicher, S.; Grimme, S., Extended tight-binding quantum chemistry methods. *Wiley Interdiscip. Rev.: Comput. Mol. Sci.* **2021,** *11*, e1493.
5. Bannwarth, C.; Ehlert, S.; Grimme, S., GFN2-xTB—an accurate and broadly parametrized self-consistent tight-binding quantum chemical method with multipole electrostatics and density-dependent dispersion contributions. *J. Chem. Theory Comput.* **2019,** *15*, 1652-1671.
6. Bannwarth, C.; Caldeweyher, E.; Ehlert, S.; Hansen, A.; Pracht, P.; Seibert, J.; Spicher, S.; Grimme, S., Extended tight-binding quantum chemistry methods. *WIREs Comput Mol Sci.* **2021,** *11*, e1493.
7. Ehlert, S.; Stahn, M.; Spicher, S.; Grimme, S., Robust and Efficient Implicit Solvation Model for Fast Semiempirical Methods. *J. Chem. Theory Comput.* **2021,** *17*, 4250-4261.
8. Maglic, J. B.; Lavendomme, R., MoloVol: an easy-to-use program for analyzing cavities, volumes and surface areas of chemical structures. *J. Appl. Crystallogr.* **2022,** *55*, 1033-1044.
9. Stewart, J. J. P., Optimization of parameters for semiempirical methods VI: more modifications to the NDDO approximations and re-optimization of parameters. *J. Mol. Model.* **2013,** *19*, 1-32.
10. MOPAC2016, James J. P. Stewart, Stewart Computational Chemistry, Colorado Springs, CO, USA, http://OpenMOPAC.net (2016).
11. Jmol: an open-source Java viewer for chemical structures in 3D, Jmol development team, http://jmol.sourceforge.net/ (2016).
